# Weekly Fluctuations in Risk Tolerance and Voting Behaviour

**DOI:** 10.1371/journal.pone.0159017

**Published:** 2016-07-08

**Authors:** Jet G. Sanders, Rob Jenkins

**Affiliations:** University of York, Heslington, York, YO10 5DD, United Kingdom; University of Reading, UNITED KINGDOM

## Abstract

Risk tolerance is fundamental to decision-making and behaviour. Here we show that individuals’ tolerance of risk follows a weekly cycle. We observed this cycle directly in a behavioural experiment using the Balloon Analogue Risk Task (Lejuez et al., 2002; Study 1). We also observed it indirectly via voting intentions, gathered from 81,564 responses across 70 opinion polls ahead of the Scottish Independence Referendum of 2014 (Study 2) and 149,064 responses across 77 opinion polls ahead of the United Kingdom European Union membership referendum of 2016 (Study 3). In all three studies, risk-tolerance decreased from Monday to Thursday before returning to a higher level on Friday. This pattern is politically significant because UK elections and referendums are traditionally held on a Thursday—the lowest point for risk tolerance. In particular, it raises the possibility that voting outcomes in the UK could be systematically risk-averse. In line with our analysis, the actual proportion of *Yes* votes in the Scottish Independence Referendum was 4% lower than forecast. Taken together, our findings reveal that the seven-day weekly cycle may have unexpected consequences for human decision-making. They also suggest that the day on which a vote is held could determine its outcome.

## Introduction

Much of human society is organised around the seven-day week [1]. Although we may think of the weekly cycle as a mere backdrop for activities, there is increasing evidence that its procession influences decision-making and behaviour in a wide range of settings [2–7]. For example, economic and public health studies have found effects of weekday on stock returns [2, 3], suicide rate [4, 5], and attendance at medical appointments [7]. To date, however, such effects have only been considered in isolation. There has been little attempt to identify changes in fundamental cognition that could account for differing decision outcomes across contexts. One aspect of cognition that is fundamental to decision-making is risk tolerance [8, 9]. We suggest that risk tolerance may be influenced by the weekly cycle as follows. First, weekday affects mood [10–13]. In particular, mood tends to be more negative near the beginning of the week (especially Mondays [10, 13]) and more positive towards the end of the week (especially Fridays and Saturdays [11, 13]). Second, mood affects decision-making [14]. In particular, people tend to be more risk tolerant in negative states such as sadness [15, 16] and more risk averse in positive states such as happiness [17]. Indeed, even weak manipulations of mood can influence decision making in laboratory studies [14]. We expected that if weekday affects mood, and mood affects risk tolerance, then weekday should affect risk tolerance. Specifically, risk tolerance should decline through the week as mood becomes more positive. Here we tested for weekly fluctuations in risk tolerance using (i) a standard laboratory measure (the Balloon Analogue Risk Task [18]; Study 1), and (ii) voting intention data from opinion polls for the Scottish Independence Referendum of 2014 (SIR [19]; Study 2) and the United Kingdom European Union membership referendum of 2016 (EUR [20]; Study 3). Our main interests were whether risk tolerance varies systematically over the weekly cycle, and whether any such variation is strong enough to impact real-world decision-making.

## Study 1: The Balloon Analogue Risk Task

We measured weekly variation in risk tolerance using the Balloon Analogue Risk Task (BART [18, 21])—a computerised task that predicts real-world risk taking in health [22–24] and economic settings [25, 26]. In this task, participants earn money by inflating a series of 20 onscreen balloons. Pressing the spacebar inflates the current balloon incrementally and increases its value by £0.01. At a variable breaking point, the balloon bursts and its value is lost. Banking an unburst balloon adds its value to the participant’s earnings, but the number of balloons is fixed. Thus each inflation confers greater risk, but also greater potential reward.

## Methods

Twenty-five paid volunteers (9 male; age range 18–27 years, M = 21.5, SD = 2.4) were recruited from the University of York participant pool (sample size based on previous research [24]) and provided written consent before partaking. To allow within-subjects comparisons, we tested each participant (N = 25) on each weekday Monday–Friday (5 sessions per participant, 125 test sessions in total; see S1 Table). To rule out any order effects, we staggered participants’ test sessions as follows: 5 were tested on Mon, Tue, Wed, Thu, Fri (in that order); 5 were tested on Tue, Wed, Thu, Fri, Mon; 5 were tested on Wed, Thu, Fri, Mon, Tue; 5 were tested on Thu, Fri, Mon, Tue, Wed; and 5 were tested on Fri, Mon, Tue, Wed, Thu. In this counterbalanced design, any potential effects of task practice or wealth accumulation are orthogonal to weekday and fall out of the weekday analysis. To rule out possible time of day effects [27], participants were tested at the same time in each of their five test sessions, and mean start time was matched across weekdays (13:00). Data was collected over the three-month period April–June 2014. To avoid phase shifting of weekday effects [13], testing was suspended for weeks that contained a Bank Holiday Monday (a public holiday in the UK). The BART task, administered via PsychoPy [21], consisted of 20 balloons with a mean bursting point of 59 pumps (SD 35 pumps) and an incremental value of £0.01 per pump.

Onscreen task instructions for the BART were as follows: “This is a game where you have to optimise your earnings in a balloon pumping competition. You get prize money for each balloon you pump up, according to its size. But if you pump it too far it will pop and you’ll get nothing for that balloon. Balloons differ in their maximum size–they can occasionally reach to almost the size of the screen–but most will pop before that. Whatever you earn in the task you will be paid as a bonus at the end of the session. Press SPACE to pump the balloon, RETURN to bank the cash for this balloon and move onto the next”.

Participants were paid their earnings on the BART task daily. To incentivise return, base rate payments (£3 per session) were withheld until the end of the final session (5 sessions x £3 = £15). At the end of the final session, participants were asked to guess the aim of the experiment. None of the guesses related to risk tolerance or weekday effects. Data from one participant who withdrew due to illness was excluded from the analysis. Ethics approval was granted by the Department of Psychology Ethics Committee, University of York.

## Results

**Fig 1:**
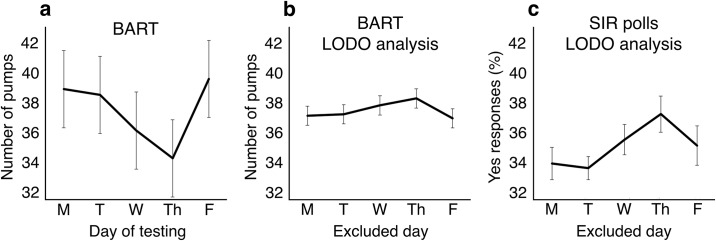
Weekday effects on risk tolerance and voting intention. Plots show mean ± SEM. Mean adjusted BART scores (a) as a function of weekday. Risk tolerance is lowest on Thursdays. Leave One Day Out (LODO) analysis of BART data (b). Excluding data from Thursdays increases risk tolerance. LODO analysis of *Yes* responses (c) in opinion poll data from the SIR. Excluding responses from Thursdays increases the proportion of *Yes* responses.

For each participant in each test session, we quantified behavioural risk taking using the recommended BART adjusted mean score—the average number of pumps for unburst balloons [18]. Overall scores for each weekday were calculated by averaging the scores of all 24 participants on each day (Fig 1a and S2 Table). Pairwise comparisons (Fisher’s LSD) are summarised in S3 Table.

BART adjusted mean scores [18] were lowest on Thursdays M = 34.25, SE = 2.02, CI = 30.07–38.43] and highest on Fridays [M = 39.55, SE = 2.05, CI = 35.31–43.79] (Fig 1a). One-way repeated-measures ANOVA revealed a significant effect of weekday on BART scores [F (4,92) = 2.90, p = .026, d = .263]. Pairwise comparisons (Fisher’s LSD) showed that risk tolerance was significantly lower on Thursdays than on Mondays [M = 38.86, SE = 2.67, CI = 33.34–44.39; p = .022], Tuesdays [M = 38.48, SE = 2.42, CI = 33.48–43.48; p = .044], and Fridays [p = .003], and was significantly higher on Fridays than on Thursdays and Wednesdays [M = 36.10, SE = 2.23, CI = 31.48–40.73; p = .042]. 17 out of 24 participants (70.8%) showed a midweek dip in risk tolerance, with lower BART adjusted mean scores for Tuesday, Wednesday, and Thursday combined than for Monday and Friday combined. Moreover, the proportion of participants for whom Thursday was the most risk-averse day (41.7%) was more than double the proportion expected by chance (20%) [two-tailed binomial test, p = .025].

Although risk tolerance showed a clear monotonic decline from Monday to Thursday [7], one aspect of the results that we did not anticipate is the distinctive uptick in risk tolerance on Friday. In previous studies, Friday and Saturday have been associated with the most positive moods of all weekdays [11, 13]. If we accept that risk tolerance decreases as mood improves [14–17], then we should expect Friday to continue the monotonic decline seen for Monday to Thursday. The Friday recovery suggests that risk tolerance may track *prospective* mood more closely than it tracks *current* mood. On this account, risk tolerance is lowest on Thursday because that is when the most rewarding part of the week is imminent. When gain is expected, change is resisted (risk aversion). When loss is expected, change is welcomed (risk tolerance).

Given the large effect of weekday on behaviour in the BART task (e.g. a difference of 5 key presses between Thursdays and Fridays), we next examined risk-taking behaviour in a real-world setting. Specifically, we tested whether decisions taken on Thursdays are more risk averse than decisions taken on other days.

## Study 2: Scottish Independence Referendum

Political elections in the UK provide a convenient test for weekly fluctuations in risk tolerance because they are traditionally held on Thursdays. Our finding of decreased risk tolerance on Thursdays raises the possibility that voting outcomes in the UK could be systematically risk averse. We note that a common synonym for *risk averse* is *conservative*; and indeed there is a substantial literature associating risk aversion with political conservatism or the political right [28–33]. Risk aversion has also been linked to voters’ majority threshold preferences [34, 35]. In view of these associations, we wondered whether weekly fluctuations in risk tolerance could also be detected as weekly fluctuations in political viewpoint. If so, then sampling political opinion on Thursdays amounts to sampling it in its most conservative phase.

Assigning risk levels to voting decisions in UK General Elections is complex, because each decision involves a wide variety of political issues and a large number of possible response options. To circumvent these complexities, we focused on the Scottish Independence referendum of Thursday 18^th^ September 2014. Unlike General Elections, this referendum vote concerned a single issue (national independence) and offered only two response options (*Yes* or *No*). Consistent with the correlation between risk tolerance and political viewpoint, the *Yes* campaign was driven more by the political left than by the political right [35–37]. Conversely, the *No* campaign drew support more from the political centre and right than from the political left [35–37]. In view of our findings from Study 1, our main interest was whether holding the referendum on a Thursday made a *No* vote more likely.

## Methods

As the referendum occurred only once, we could not compare voting outcomes across different weekdays. However, voting intentions were polled extensively in the months leading up to the election [19]. These opinion polls gave us the opportunity to examine whether weekday influenced voting intention.

To include all of the available high quality data, we downloaded from whatscotlandthinks.org the results of all 70 opinion polls conducted between 1^st^ February 2013 and 8^th^ July 2014 (81,564 respondents in total; median sample size 1006 people, range 549–10,007 people; see [http://bit.ly/1ncTTTe] for survey methodology and raw data).

Because each of these polls collected responses over more than one day of the week (median duration 6 days, range 2–121 days), it was not possible to compare voting intention across individual weekdays directly. Instead we estimated the influence of each weekday Monday–Friday by conducting a Leave One Day Out (LODO) analysis [38], in which poll results from each day in turn were excluded (see Supplemental Materials). We reasoned that if Thursday is the most risk-averse day, then *excluding* Thursdays should disproportionately exclude *No* responses, thereby inflating the proportion of *Yes* responses in the remaining data.

To verify the sensitivity of LODO analysis to weekday effects, we first reanalysed the data from the BART experiment using this approach.

## Results

### BART LODO analysis

We computed separate mean adjusted BART scores for each LODO category by pooling data from all test days excluding Mondays (37.09), Tuesdays (37.19), Wednesdays (37.78), Thursdays (38.25), and Fridays (36.92). Note that because this analysis *removes*, rather than *isolates*, the influence of each day, it reverses the direction of the effect as plotted (Fig 1b and S4 Table). The analysis also reduces the apparent magnitude of the effects because it collapses across data from different days that are associated with different levels of risk tolerance (Fig 1b). To verify that effects of weekday could be reliably detected in this situation, we conducted separate repeated measures t-tests (two-tailed) comparing mean adjusted BART scores for each weekday Monday–Friday against the complement. As expected, risk tolerance in the BART task was higher when Thursdays were excluded than when any other day was excluded (Fig 1b). Paired sample t-tests revealed a significant difference for Thursdays versus all other weekdays combined, and no significant differences for the other weekdays. [Monday Only M = 38.86, Monday Excluded M = 37.09, *t* (23) = 1.12, *p* > .250, d = .23; Tuesday Only M = 38.48, Tuesday Excluded M = 37.19, *t* (23) = 0.85, *p* > .250, d = .17; Wednesday Only M = 36.10, Wednesday Excluded M = 37.78, *t* (23) = -1.30, *p* = .207, d = -.26; Thursday Only M = 34.25, Thursday Excluded M = 38.25, *t* (23) = -2.73, *p* = .012, d = -.56; Friday Only M = 39.55, Friday Excluded M = 36.92, *t* (23) = 1.92, *p* = .067, d = .39].

### Scottish Independence Referendum LODO analysis

For each poll, we determined the days over which data was collected by converting the published start and end dates [19] to days of the week. We then categorised each poll according to whether its data collection period included or excluded each weekday Monday–Friday (S5 Table). Mean percentage *Yes* responses were computed separately for polls that excluded Monday [10 polls, 10,233 respondents, 33.90% *Yes*], Tuesday [18 polls, 18,917 respondents 33.61% *Yes*], Wednesday [14 polls, 14,621 respondents, 35.50% *Yes*], Thursday [14 polls, 14,681 respondents, 37.21% *Yes*], and Friday [10 polls, 10,831 respondents, 35.10% *Yes*]. To test the effect of each weekday on voting intention, we conducted Mann–Whitney tests for independent samples (two-tailed) comparing the percentage of *Yes* votes for polls that *included* versus *excluded* each weekday [Monday Excluded N = 10, M = 33.90, CI = 27.21–40.59, Monday Included N = 60, M = 33.88, CI = 24.53–43.23, *U* = 300, *p* > .250, *r* = 0; Tuesday Excluded N = 18, M = 33.61 CI = 27.09–40.13, Tuesday Included N = 52, M = 33.98, CI = 24.25–43.71, *U* = 495, *p* > .250, r = -.04; Wednesday Excluded N = 14, M = 35.50, CI = 28.10–42.90, Wednesday Included N = 56, M = 33.48, CI = 24.27–42.70, *U* = 282, *p* = .103, r = -.19; Thursday Excluded N = 14, M = 37.21, CI = 28.31–46.12, Thursday Included N = 56, M = 33.05, CI = 24.76–41.35, *U* = 186.5, *p* = .002, r = -.36; Friday Excluded N = 10, M = 35.10, CI = 26.97–43.23, Friday Included N = 60, M = 33.68, CI = 24.57–42.79, *U* = 256, *p* > .250, r = -.09]. Thus, the proportion of *Yes* votes was significantly lower in polls that *included* Thursday than in polls that *excluded* Thursday, consistent with lower risk tolerance on Thursdays. No other day of the week showed this pattern.

An alternative convention for analysing opinion poll data is to remove *Don’t Know* responses from the denominator before calculating the percentage of *Yes* responses [19]. The rationale for this measure is that *Don’t Know* is not an option in the actual vote. Adopting this alternative convention did not alter the overall pattern [Monday Excluded N = 10, M = 40.70, CI = 33.19–48.21, Monday Included N = 60, M = 41.07, CI = 31.98–50.15, *U* = 318, *p* > .250, r = .04; Tuesday Excluded N = 18, M = 39.33, CI = 32.03–46.64, Tuesday Included N = 52, M = 41.60, CI = 32.51–50.68, *U* = 615, *p* = .048, r = .24; Wednesday Excluded N = 14, M = 41.50, CI = 32.74–50.26, Wednesday Included N = 56, M = 40.89, CI = 31.98–49.81, *U* = 352, *p* > .250, r = -.07; Thursday Excluded N = 14, M = 43.57, CI = 33.18–53.97, Thursday Included N = 56, M = 40.38, CI = 32.35–48.40, *U* = 228, *p* = .016, r = -.29; Friday Excluded N = 10, M = 41.70, CI = 31.62–51.78, Friday Included N = 60, M = 40.90, CI = 32.22–49.58, *U* = 264.5, *p* > .250, r = -.07].

### Scottish Independence Referendum purdah period forecast and actual outcome

We predicted that the actual *Yes* vote would be lower than polls forecast because the referendum took place on Thursday, whereas forecasts pooled voting intention across weekdays, thus overestimating risk tolerance. To obtain a stable and up-to-date forecast, we calculated the mean proportion of *Yes* votes for the twenty opinion polls that were conducted in the pre-election purdah period (21^st^ August–18^th^ September 2014). In UK law, purdah refers to a pre-election period of 4–6 weeks that prevents central and local government from announcing new initiatives that could influence the outcome of the vote [http://bit.ly/172KV7W]. Consistent with settling voter opinion, these 20 polls showed *Don’t Know* responses decreasing as voting day approached (S6 Table). The mean proportion of *Yes* votes in these final 20 polls (*Don’t Know* responses removed to allow comparison with the actual outcome) was 48.7% (range = 46–59%, SD = 3.0), reported in the media as too close to call [http://bbc.in/1uqbYBA]. In the event, 3,619,915 people cast a vote. 1,617,989 people voted *Yes* (44.7%) and 2,001,926 people voted *No* (55.3%). Consistent with our analysis, the actual proportion of *Yes* votes was 4% lower than forecast, and lower than predicted by *any* of the 20 polls in the purdah period.

## Study 3: United Kingdom European Union membership referendum

In our analysis of the Scottish Independence Referendum, we identified the *Yes* campaign with risk tolerance because the *Yes* campaign drew support mainly from the political left. Conversely, we identified the *No* campaign with risk aversion because the *No* campaign drew support mainly from the political right. The weekday profile for *Yes* responses in Study 2 certainly resembles that seen for risk tolerance in Study 1. Nevertheless, the pattern in voting intentions could be shaped by other factors besides risk tolerance. For example, the observed decline in *Yes* responses could be driven by a preference for being part of a larger whole (the UK), or a preference for continuity over change. The SIR does not allow us to separate these drivers because they tended to bundle into a *Yes* pole (political left, preference for an independent unit, preference for change) and a *No* pole (political right, preference for a larger whole, preference for continuity). To characterise the weekday effect more precisely, we next sought a situation in which political viewpoint bundled with these other factors in the opposite combination (political *left*, preference for a larger whole, preference for continuity; political *right*, preference for an independent unit, preference for change). The UK referendum on membership of the European Union (EUR) meets these requirements. Unlike the SIR, the *Leave* campaign (cf. *Yes* campaign) is led mainly by the political right, and the *Remain* campaign (cf. *No* campaign) is led mainly by the political left. This opposite bundling provides a critical test of weekly fluctuations in risk tolerance because it generates opposing predictions: if the Thursday effect reflects a preference for *belonging to a larger whole* or for *continuity* rather than conservative risk aversion, then the weekday profile for the EUR data should be the same as for the SIR data. That is, the proportion of *Leave* responses should be lower on Thursday than on other days, corresponding to a Thursday peak in the LODO analysis. Alternatively, if the Thursday effect reflects conservative risk aversion (as seen in Study 1), then the weekday profile for the EUR data should be *opposite* to that seen in the SIR data. That is, the proportion of *Leave* responses should be *higher* on Thursday than on other days, corresponding to a Thursday dip in LODO analysis.

## Methods

Our approach to analysing the EUR data was the same as for the SIR data in Study 2. The results of all 77 opinion polls conducted between 4^th^ September 2015 and 24^th^ March 2016 were downloaded from whatukthinks.org (149,064 respondents in total; median sample size 2000 people, range 513–11,171 people; see [http://bit.ly/1ophEx7] for survey methodology and raw data).

As in Study 2, we estimated the influence of each weekday Monday–Friday by conducting a Leave One Day Out (LODO) analysis, in which poll results from each day in turn were excluded (see Supplemental Materials). Almost all of these polls collected responses over more than one day of the week (median duration 3 days, range 1–12 days). We expected that if Thursday is the most risk-averse day, then *excluding* Thursdays should disproportionately exclude *Leave* responses, thereby reducing the proportion of *Leave* responses in the remaining data.

## Results

### EU referendum polls LODO analysis

**Fig 2:**
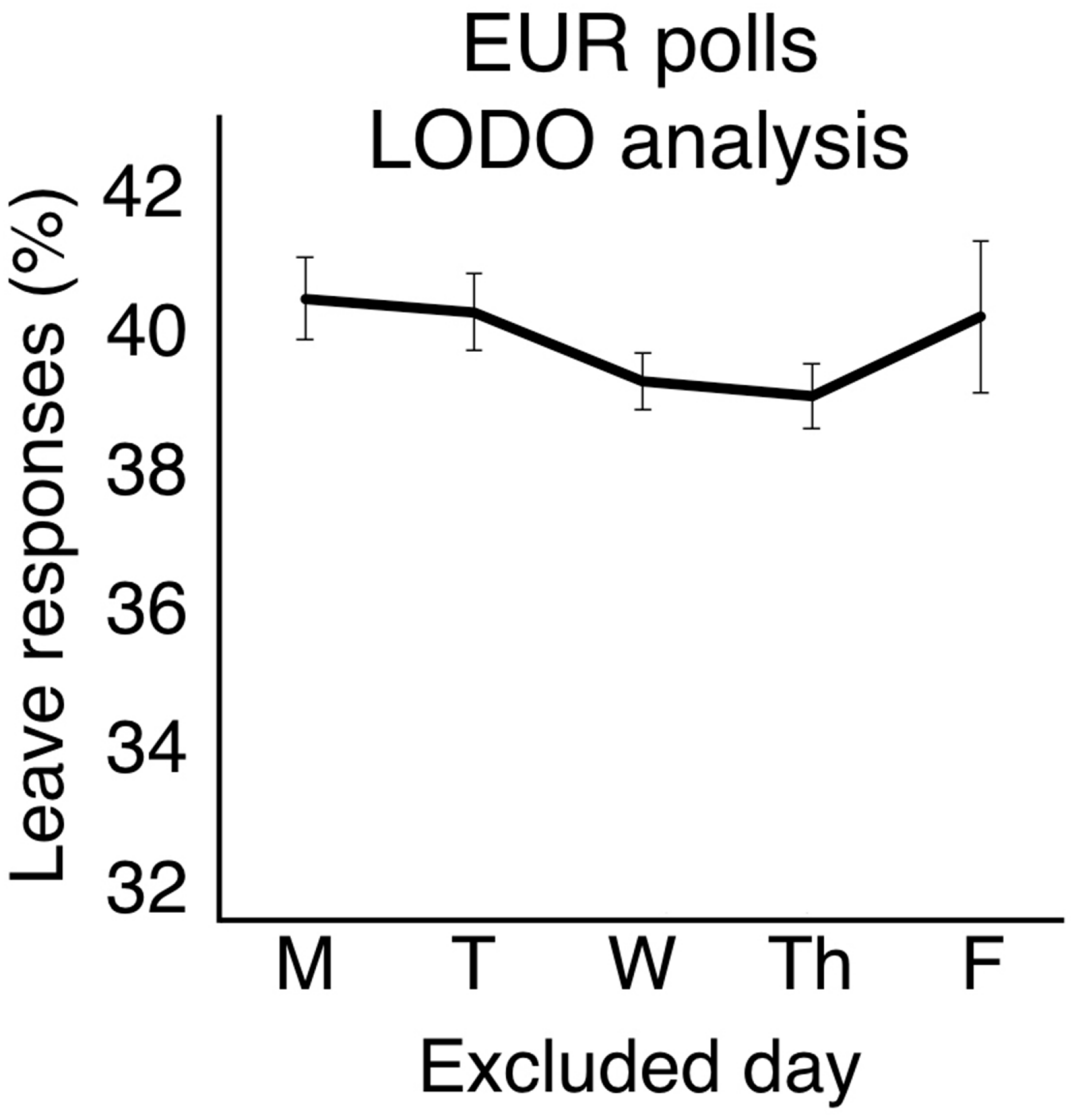
LODO analysis of *Leave* responses from EUR opinion polls (mean ± SEM). Critically, the weekday profile is *opposite* to that seen in the SIR data. Excluding responses from Thursdays *decreases* the proportion of *Leave* responses.

As in Study 2, we determined the days over which data was collected by converting the published start and end dates to days of the week. We then categorised each poll according to whether its data collection period included or excluded each weekday Monday–Friday (S7 Table). Mean percentage *Leave* responses were computed separately for polls that excluded Monday [48 polls, 85,347 respondents, 40.40% *Leave*], Tuesday [57 polls, 95,115 respondents 40.21% *Leave*], Wednesday [56 polls, 95,246 respondents, 39.21% *Leave*], Thursday [48 polls, 82,474 respondents, 30.00% *Leave*], and Friday [22 polls, 44,342 respondents, 40.29% *Leave*]. To test the effect of each weekday on voting intention, we conducted Mann–Whitney tests for independent samples (two-tailed) comparing the percentage of *Leave* votes for polls that *included* versus *excluded* each weekday [Monday Excluded N = 48, M = 40.40, CI = 33.37–46.63, Monday Included N = 29, M = 40.00, CI = 32.40–48.39, *U* = 707, *p* > .250, *r* = -.01; Tuesday Excluded N = 57, M = 40.21, CI = 34.87–51.13, Tuesday Included N = 20, M = 40.35, CI = 37.67–48.33, *U* = 635.5, *p* > .250, *r* = -.09; Wednesday Excluded N = 56, M = 39.21, CI = 36.98–49.02, Wednesday Included N = 21, M = 43.00, CI = 34.62–51.38, *U* = 923.5, *p* < .001, *r* = -.44; Thursday Excluded N = 48, M = 39.00 CI = 36.68–49.32, Thursday Included N = 29, M = 42.31, CI = 35.43–50.57, *U* = 1080, *p* < .001, *r* = -.46; Friday Excluded N = 22, M = 40.14, CI = 33.04–52.96, Friday Included N = 55, M = 40.29, CI = 33.97–46.61, *U* = 649, *p* > .250, *r* = -.06]. Thus, the proportion of *Leave* votes was significantly higher in polls that *included* Thursday than in polls that *excluded* Thursday, consistent with lower risk tolerance on Thursdays (Fig 2). On this occasion, a significant difference was also observed for polls that *included* Wednesday compared to polls that *excluded* Wednesday (the second most risk-averse day in all three studies), presumably due to the much larger sample. No other day of the week showed this pattern.

Once again, removing *Don’t Know* responses from the denominator before calculating the percentage of *Leave* responses did not alter the overall pattern [Monday Excluded N = 48, M = 47.73, CI = 41.31–54.15, Monday Included N = 29, M = 47.72, CI = 38.89–56.56.15, *U* = 826, *p* > .250, r = -.16; Tuesday Excluded N = 57, M = 47.28, CI = 43.40–58.98, Tuesday Included N = 20, M = 49.01, CI = 43.46–54.27, *U* = 753, *p* = .033, r = -.24; Wednesday Excluded N = 56, M = 46.78, CI = 43.81–58.57, Wednesday Included N = 21, M = 50.26, CI = 44.31–53.41, *U* = 925, *p* < .001, r = -.44; Thursday Excluded N = 48, M = 46.51, CI = 43.56–58.83, Thursday Included N = 29, M = 49.74, CI = 44.01–53.72, *U* = 1063, *p* < .001, r = -.44; Friday Excluded N = 22, M = 47.83, CI = 38.95–58.78, Friday Included N = 55, M = 47.83, CI = 41.66–53.99, *U* = 570, *p* > .250, r = -.05].

## General Discussion

A repeated-measures BART study revealed a reliable weekly cycle in risk tolerance, with Thursday being the most risk-averse day. We found similar weekly cycles in two real-world examples of decision-making under uncertainty—voting intentions ahead of the Scottish Independence Referendum, and voting intentions ahead of the EU Referendum. In both sets of opinion polls, responses were more aligned with the political right on Thursdays than on other days of the week, consistent with established links between risk aversion and political conservatism.

Although it is logically possible that the weekly cycle could affect responses to opinion polls but not affect actual voting behaviour, there are several reasons to doubt that. First, previous studies confirm that both attitudes [39, 40] and intentions [41–43] predict behaviour. Second, in the context of the present studies, we found similar effects of the weekly cycle in expressed intentions (Studies 2 & 3) and in actual behaviour (Study 1). Third, the outcome of the Scottish Independence Referendum in 2014 bore out our analyses. As we predicted, the risk-averse *No* vote exceeded pollsters' forecasts, by some 4%. The outcome of the EU referendum is unknown at the time of writing. However, our analysis suggests that holding the referendum on Thursday will inflate the proportion of *Leave* votes. Effects of weekday may be especially pronounced when the proportion of undecided voters is high and implicit attitudes influence their decisions [44, 45].

Whatever the outcome of the EUR, all three of our studies reveal systematic changes in responses through the week. In all three studies, the direction of change from Monday was towards conservative risk aversion, and in all three studies, Thursday was the inflection point. These findings could be explained by assuming that (i) response to risk becomes more conservative as mood improves [14–17], and that (ii) this influence of mood has a prospective component [8, 46]. When we view prospective outcomes as good, we do not want to jeopardise those outcomes, and so we become more risk averse. When we view prospective outcomes as bad, we are motivated to find alternatives, and so we become more risk tolerant. The challenge now is to understand how these factors interact to produce the observed behaviour.

Although the present analyses focus on two recent referendums, we note that every UK general election since 1935 has been held on a Thursday. Our findings suggest that this tradition of Thursday voting may consistently bias UK elections towards risk-averse outcomes. Comparison data from a country that votes on a different day of the week would be interesting in this respect. For example, American elections are traditionally held on Tuesday—a more risk-tolerant day than Thursday in the present studies. The corollary is that the observed risk-averse bias should affect voting in the UK, but not voting in the US.

Given the connections between risk aversion and political conservatism it is worth noting that risk-averse voting need not necessarily mean voting for a politically conservative party. Risk-averse voting could plausibly entail voting the way you have always voted rather than trying something new, or voting for whichever outcome has the most support. However risk aversion manifests itself at the polls, it may be possible to reduce systematic effects of weekday by allowing voting over several days (as with postal voting), or by distributing election days more evenly through the weekly cycle.

Our findings suggest a number of interesting directions for future research. Given that risk evaluation is a core aspect of cognition [8, 9], it seems likely that weekly fluctuations in risk tolerance could influence decision-making and behaviour in other domains too (e.g. personal, economic, or medical decision-making). Analysing data from such domains should help to characterise weekday effects and their scope. A related question is whether other aspects of cognition besides risk evaluation also change systematically over the week [13]. If, say, personality or memory performance follows a weekly cycle, the implications could be profound—not only for everyday behaviour, but also for interpretation of psychological findings.

Our current findings suggest that combining experimental methods with reanalysis of large datasets will be a fruitful approach to the larger project of understanding psychological effects of weekday. They also show that the human invention of the seven-day week has unintended consequences: the outcome of a decision can depend on the day on which it is taken.

## Supporting Information

S1 Table Testing schedule for the BART experiment. Rows show times and columns show dates. Cells contain participant numbers (p1–25) and session numbers (sess1–5).
(PDF)

S2 Table BART adjusted mean scores for participants in the behavioural experiment. Higher numbers indicate higher risk tolerance. Rows denote participant numbers, columns denote weekday. Higher numbers indicate higher risk tolerance.
(PDF)

S3 Table Pairwise comparisons of BART adjusted mean scores for each weekday Monday–Friday. (* p < .05; ** p < .01).
(PDF)

S4 Table Leave One Day Out (LODO) analysis of BART adjusted mean scores. Rows denote participant numbers, columns denote the excluded day. Higher numbers indicate lower risk tolerance.
(PDF)

S5 Table Leave One Day Out (LODO) analysis of voting intentions ahead of the Scottish Independence Referendum. The first block shows all polls coded according to the days on which data was collected. Subsequent blocks show polls that include versus exclude each weekday in turn. Rows refer to separate polls. Columns contain response numbers, start and end dates, start and end days, presence (1) or absence (0) of each weekday, and poll results.
(PDF)

S6 Table Data from the twenty opinion polls that were conducted during the purdah period. *Don’t Know* responses are removed (9) to allow direct comparison with the actual referendum outcome. Rows denote intention polls, columns denote response numbers, start and end date and day, which weekday was included and poll outcomes.
(PDF)

S7 Table Leave One Day Out (LODO) analysis of voting intentions ahead of the United Kingdom European Union membership referendum. The first block shows all polls coded according to the days on which data was collected. Subsequent blocks show polls that include versus exclude each weekday in turn. Rows refer to separate polls. Columns contain response numbers, start and end dates, start and end days, presence (1) or absence (0) of each weekday, and poll results.
(PDF)
