# Supplementary material for: Weekly Fluctuations in Risk Tolerance and Voting Behaviour
Source: PLoS One. 2016 Jul 8;11(7):e0159017. doi: 10.1371/journal.pone.0159017 (PMC4938543; doi:10.1371/journal.pone.0159017)
Supplement: S1 Table — Cells contain participant numbers (p1–25) and session numbers (sess1–5). (PDF) [file pone.0159017.s001.pdf]

S1 Table. Testing schedule for the BART experiment. Rows show times and columns show dates. Cells contain participant numbers (p1–25) and session numbers (sess1–5).

	Monday 7th April	Tuesday 8th April	Wednesday 9th April	Thursday 10th April	Friday 11th April	WEEKEND	Monday 14th April	Tuesday 15th April	Wednesday 16th April	Thursday 17th April	Friday 18th April
09:00											
10:00		p2sess1	p2sess2	p2sess3	p2sess4		p2sess5				
11:00					p5sess1		p5sess2	p5sess3	p5sess4	p5sess5	
12:00			p3sess1	p3sess2	p3sess3		p3sess4	p3sess5			
13:00	p1sess1	p1sess2	p1sess3	p1sess4	p1sess5						
14:00				p4sess1	p4sess2		p4sess3	p4sess4	p4sess5		
15:00											
16:00											
17:00											
18:00											
	12th May	13th May	14th May	15th May	16th May		19th May	20th May	21st May	22nd May	23rd May
09:00	p6sess1	p6sess2	p6sess3	p6sess4	p6sess5						
10:00		p8sess1	p8sess2	p8sess3	p8sess4		p8sess5				
11:00			p10sess1	p10sess2	p10sess3		p10sess4	p10sess5			
12:00	p7sess1	p7sess2	p7sess3	p7sess4	p7sess5						
13:00					p14sess1		p14sess2	p14sess3	p14sess4	p14sess5	
14:00				p12sess1	p12sess2		p12sess3	p12sess4	p12sess5		
15:00					p15sess1		p15sess2	p15sess3	p15sess4	p15sess5	
16:00		p9sess1	p9sess2	p9sess3	p9sess4		p9sess5				
17:00			p11sess1	p11sess2	p11sess3		p11sess4	p11sess5			
18:00				p13sess1	p13sess2		p13sess3	p13sess4	p13sess5		
	2nd June	3rd June	4th June	5th June	6th June		9th June	10th June	11th June	12th June	13th June
09:00					p24sess1		p24sess2	p24sess3	p24sess4	p24sess5	
10:00			p20sess1	p20sess2	p20sess3		p20sess4	p20sess5			
11:00				p22sess1	p22sess2		p22sess3	p22sess4	p22sess5		
12:00		p18sess1	p18sess2	p18sess3	p18sess4		p18sess5				
13:00				p23sess1	p23sess2		p23sess3	p23sess4	p23sess5		
14:00	p16sess1	p16sess2	p16sess3	p16sess4	p16sess5						
15:00			p21sess1	p21sess2	p21sess3		p21sess4	p21sess5			
16:00	p17sess1	p17sess2	p17sess3	p17sess4	p17sess5						
17:00					p25sess1		p25sess2	p25sess3	p25sess4	p25sess5	
18:00		p19sess1	p19sess2	p19sess3	p19sess4		p19sess5				
