# Supplementary material for: Weekly Fluctuations in Risk Tolerance and Voting Behaviour
Source: PLoS One. 2016 Jul 8;11(7):e0159017. doi: 10.1371/journal.pone.0159017 (PMC4938543; doi:10.1371/journal.pone.0159017)
Supplement: S2 Table — Higher numbers indicate higher risk tolerance. Rows denote participant numbers, columns denote weekday. Higher numbers indicate higher risk tolerance. (PDF) [file pone.0159017.s002.pdf]

S2 Table. BART adjusted mean scores for participants in the behavioral experiment. Higher numbers indicate higher risk tolerance. Rows denote participant numbers, columns denote weekday. Higher numbers indicate higher risk tolerance.

Participant	Mon	Tue	Wed	Thu	Fri
1	50.00	52.28	45.00	45.45	45.83
2	21.63	11.28	11.64	13.67	28.67
3	50.00	37.83	33.33	43.85	51.67
4	37.08	38.58	36.15	34.45	41.08
5	64.00	51.10	50.08	50.00	55.45
6	51.11	42.27	38.75	31.91	32.50
7	29.67	36.25	33.07	36.00	37.14
8	29.57	31.46	34.00	29.20	33.85
9	23.44	22.50	20.00	18.75	21.17
10	55.41	47.73	37.69	46.80	46.33
11	31.80	51.00	26.31	25.87	28.06
12	26.88	31.40	33.64	18.93	23.43
13	46.81	45.83	47.33	35.55	47.07
14	31.43	30.00	35.71	38.46	44.23
15	25.69	23.73	28.67	23.85	48.00
16	22.94	33.27	32.50	36.15	36.15
17	59.66	32.64	39.46	38.92	40.15
18	27.13	24.00	25.44	25.44	25.88
19	32.33	45.00	36.15	38.21	34.61
20	41.54	45.00	29.67	35.67	43.07
21	54.55	53.64	46.40	45.80	51.81
22	50.00	61.00	64.44	27.30	55.25
23	25.86	31.43	31.00	33.40	31.29
24	44.18	44.30	50.08	48.33	46.45
Mean	38.86	38.48	36.10	34.25	39.55
SE	2.67	2.42	2.23	2.02	2.05
