# Supplementary material for: Weekly Fluctuations in Risk Tolerance and Voting Behaviour
Source: PLoS One. 2016 Jul 8;11(7):e0159017. doi: 10.1371/journal.pone.0159017 (PMC4938543; doi:10.1371/journal.pone.0159017)
Supplement: S3 Table — (* p < .05; ** p < .01). (PDF) [file pone.0159017.s003.pdf]

S3 Table. Pairwise comparisons of BART adjusted mean scores for each weekday Monday–Friday. (* p < .05; ** p < .01).

Difference (M)	Mon	Tue	Wed	Thu	Fri
Mon		0.382	2.758	*1.874	-0.685
Tue	-0.382		2.376	*4.323	-1.067
Wed	-2.758	-2.376		1.857	*-3.443
Thu	*-1.874	*-4.323	-1.857		**-5.299
Fri	0.685	1.067	*3.443	**5.299	
