# Supplementary material for: Weekly Fluctuations in Risk Tolerance and Voting Behaviour
Source: PLoS One. 2016 Jul 8;11(7):e0159017. doi: 10.1371/journal.pone.0159017 (PMC4938543; doi:10.1371/journal.pone.0159017)
Supplement: S4 Table — Rows denote participant numbers, columns denote the excluded day. Higher numbers indicate lower risk tolerance. (PDF) [file pone.0159017.s004.pdf]

S4 Table. Leave One Day Out (LODO) analysis of BART adjusted mean scores. Rows denote participant numbers, columns denote the excluded day. Higher numbers indicate lower risk tolerance.

Participant	Mon Excl.	Tue Excl.	Wed Excl.	Thu Excl.	Fri Excl.
1	47.14	46.57	48.39	48.28	48.18
2	16.32	18.90	18.81	18.31	14.56
3	41.67	44.71	45.84	43.21	41.25
4	37.57	37.19	37.80	38.22	36.57
5	51.66	54.88	55.14	55.16	53.80
6	36.36	38.57	39.45	41.16	41.01
7	35.62	33.97	34.76	34.03	33.75
8	32.13	31.66	31.02	32.22	31.06
9	20.61	20.84	21.46	21.78	21.17
10	44.64	46.56	49.07	46.79	46.91
11	32.81	28.01	34.18	34.29	33.74
12	26.85	25.72	25.16	28.84	27.71
13	43.94	44.19	43.81	46.76	43.88
14	37.10	37.46	36.03	35.34	33.90
15	31.06	31.55	30.32	31.52	25.48
16	34.52	31.94	32.13	31.22	31.22
17	37.79	44.55	42.84	42.98	42.67
18	25.19	25.97	25.61	25.61	25.50
19	38.49	35.33	37.54	37.02	37.92
20	38.35	37.49	41.32	39.82	37.97
21	49.41	49.64	51.45	51.60	50.10
22	52.00	49.25	48.39	57.67	50.69
23	31.78	30.39	30.50	29.90	30.42
24	47.29	47.26	45.82	46.25	46.72
Mean	37.09	37.19	37.78	38.25	36.92
SE	9.30	9.66	9.76	10.15	10.05
