# Supplementary material for: Weekly Fluctuations in Risk Tolerance and Voting Behaviour
Source: PLoS One. 2016 Jul 8;11(7):e0159017. doi: 10.1371/journal.pone.0159017 (PMC4938543; doi:10.1371/journal.pone.0159017)
Supplement: S5 Table — The first block shows all polls coded according to the days on which data was collected. Subsequent blocks show polls that include versus exclude each weekday in turn. Rows refer to separate polls. Columns contain response numbers, start and end dates, start and end days, presence (1) or absence (0) of each weekday, and poll results. (PDF) [file pone.0159017.s005.pdf]

S5 Table. Leave One Day Out (LODO) analysis of voting intentions ahead of the Scottish Independence Referendum. The first block shows all polls coded according to the days on which data was collected. Subsequent blocks show polls that *include* versus *exclude* each weekday in turn. Rows refer to separate polls. Columns contain response numbers, start and end dates, start and end days, presence (1) or absence (0) of each weekday, and poll results.

ALL POLLS															
Poll details	Number of responses	Start date	Start day	End date	End day	Total days	Monday?	Tuesday?	Wednesday?	Thursday?	Friday?	Yes%	No%	Don't Know%	Yes% (DK removed)
Angus Reid; Mail on Sunday/ 30/1-1/2/13	1003	1/30/13	Wednesday	2/1/13	Friday	3	0	0	1	1	1	32	47	20	41
MORI; Times/ 4-9/2/13	1003	2/4/13	Monday	2/9/13	Saturday	6	1	1	1	1	1	34	55	11	38
TNS-BMRB; CND 20-28/2/13	1001	2/20/13	Wednesday	2/28/13	Thursday	9	1	0	1	1	1	33	52	15	39
Ashcroft (ORB) 22.2.-9.5.13	10007	2/22/13	Friday	5/9/13	Thursday	77	1	1	1	1	1	26	65	10	29
Panelbase/Sunday Times 18-22/3/13	885	3/18/13	Monday	3/22/13	Friday	5	1	1	1	1	1	36	46	18	44
TNS-BMRB; Herald 20/3-2/4.13	1002	3/20/13	Wednesday	4/3/13	Wednesday	15	1	1	1	1	1	30	51	19	37
MORI/Times 29.4-5.5.13	1001	4/29/13	Monday	5/5/13	Sunday	7	1	1	1	1	1	31	59	10	34
Ashcroft (YouGov)/ 29.4-2.5.13	1236	4/29/13	Monday	5/2/13	Thursday	4	1	1	1	1	0	30	56	12	35
Panelbase/Sunday Times 10-16/5/13	1004	5/10/13	Friday	5/16/13	Thursday	7	1	1	1	1	1	36	44	20	45
SSA 25.6-23.10.13	1497	6/25/13	Tuesday	10/23/13	Wednesday	121	1	1	1	1	1	30	54	16	36
ICM/Scotland on Sunday 7-11.7.13	1002	7/7/13	Sunday	7/11/13	Thursday	5	1	1	1	1	0	34	45	21	43
Panelbase/Sunday Times 17-24/7/13	1001	7/17/13	Wednesday	7/24/13	Wednesday	8	1	1	1	1	1	37	46	17	45
Angus Reid /Sunday Express 14-16/8/13	549	8/14/13	Wednesday	8/16/13	Friday	3	0	0	1	1	1	34	47	19	42
YouGov/Devo Plus 19-22/8/13	1171	8/19/13	Monday	8/22/13	Thursday	4	1	1	1	1	0	29	59	12	33
TNS-BMRB : 21-27/8/13	1017	8/21/13	Wednesday	8/27/13	Tuesday	7	1	1	1	1	1	25	47	28	35
Panelbase/SNP 23-28/8/13	1043	8/23/13	Friday	8/28/13	Wednesday	6	1	1	1	0	1	44	43	13	51
Panelbase/Sunday Times 30.8-5.9.13	1002	8/30/13	Friday	9/5/13	Thursday	7	1	1	1	1	1	37	47	16	44
Ipsos MORI/STV 9-15.9.13	1000	9/9/13	Monday	9/15/13	Sunday	7	1	1	1	1	1	31	59	9	34
ICM/SoS/Scotsman 10-13.9.13	1002	9/10/13	Tuesday	9/13/13	Friday	4	0	1	1	1	1	32	49	19	40
YouGov/Times 13-16.9.13	1139	9/13/13	Friday	9/16/13	Monday	4	1	0	0	0	1	32	52	15	38
Progressive/Mail online 13-16.9.13	1051	9/13/13	Friday	9/16/13	Monday	4	1	0	0	0	1	27	59	14	31
TNS/BMRB 25.9-2.10.13	1004	9/25/13	Wednesday	10/2/13	Wednesday	8	1	1	1	1	1	25	44	31	36
Panelbase/Wings 17-24.10.13	866	10/17/13	Thursday	10/24/13	Thursday	8	1	1	1	1	1	37	45	17	45
TNS/BMRB 23-30.10.13	1010	10/23/13	Wednesday	10/30/13	Wednesday	8	1	1	1	1	1	25	43	32	37
Panelbase/Sunday Times 12-20.11.13	1006	11/12/13	Tuesday	11/20/13	Wednesday	9	1	1	1	1	1	38	47	15	45
TNS/BMRB 20-27.11.13	1004	11/20/13	Wednesday	11/27/13	Wednesday	8	1	1	1	1	1	26	42	32	38
YouGov (unpub) 27.11-2.12.13	1118	11/27/13	Wednesday	12/2/13	Monday	6	1	0	1	1	1	31	55	13	36
Progressive/MoS 27-29.11.13	1134	11/27/13	Wednesday	11/29/13	Friday	3	0	0	1	1	1	27	56	17	33
Ipsos MORI 29.11-5.12.13	1006	11/29/13	Friday	12/5/13	Thursday	7	1	1	1	1	1	34	57	10	37
TNS BMRB 3-10.12.13	1055	12/3/13	Tuesday	12/10/13	Tuesday	8	1	1	1	1	1	27	41	33	39
YouGov/Times 6-9.12.13	1074	12/6/13	Friday	12/9/13	Monday	4	1	0	0	0	1	33	52	13	39
TNS BMRB/Open Dem 14-20.1.14	1054	1/14/14	Tuesday	1/20/14	Monday	7	1	1	1	1	1	29	42	29	41
YouGov 21-27.1.14	1192	1/21/14	Tuesday	1/27/14	Monday	7	1	1	1	1	1	33	52	12	39
ICM/SoS 21-24.1.14	1004	1/21/14	Tuesday	1/24/14	Friday	4	0	1	1	1	1	37	44	19	46
TNS BMRB 28.1-6.2.14	996	1/28/14	Tuesday	2/6/14	Thursday	10	1	1	1	1	1	29	42	29	41
Panelbase.Sun Times 29.1-7.2.14	1012	1/29/14	Wednesday	2/7/14	Friday	10	1	1	1	1	1	37	49	14	43
Survation/MoS 29-31.1.14	1010	1/29/14	Wednesday	1/31/14	Friday	3	0	0	1	1	1	32	52	16	38
YouGov/Sun 3-5.2.14	1047	2/3/14	Monday	2/5/14	Wednesday	3	1	1	1	0	0	34	52	12	39
ICM/SoS 17-21.2.14	1004	2/17/14	Monday	2/21/14	Friday	5	1	1	1	1	1	37	49	14	43
Survation/Mail 17-18.2.14	1005	2/17/14	Monday	2/18/14	Tuesday	2	1	1	0	0	0	38	47	16	45
Panelbase/SNP 18-21.2.14	1022	2/18/14	Tuesday	2/21/14	Friday	4	0	1	1	1	1	37	47	16	44
Ipsos MORI/STV 20-24.2.14	1001	2/20/14	Thursday	2/24/14	Monday	5	1	0	0	1	1	32	57	11	36
YouGov/Sun 24-28.2.14	1257	2/24/14	Monday	2/28/14	Friday	5	1	1	1	1	1	35	53	11	40
TNS BMRB 26.2-9.3.14	1019	2/26/14	Wednesday	3/14/14	Friday	17	1	1	1	1	1	28	42	30	40
Survation/Record 6-7.3.14	1002	3/6/14	Thursday	3/7/14	Friday	2	0	0	0	1	1	39	48	13	45
Panelbase/Newsnet 7-14.3.14	1022	3/7/14	Friday	3/14/14	Friday	8	1	1	1	1	1	40	45	15	47
ICM/Sos 17-21.3.14	1010	3/7/14	Friday	3/21/14	Friday	15	1	1	1	1	1	39	46	15	45
Survation/Sunday Post 13-15.4.14	1001	3/13/14	Thursday	4/15/14	Tuesday	34	1	1	1	1	1	38	46	16	45
YouGov/Times 20-24.3.14	1072	3/20/14	Thursday	3/24/14	Monday	5	1	0	0	1	1	37	52	10	42
TNS BMRB 21.3-2.4.14	988	3/21/14	Friday	4/2/14	Wednesday	13	1	1	1	1	1	29	41	30	41
Panelbase/Wings 28.3-4.4.14	1025	3/28/14	Friday	4/4/14	Friday	8	1	1	1	1	1	41	46	14	47
Panelbase/Yes 4-9.4.14	1024	4/4/14	Friday	4/9/14	Wednesday	6	1	1	1	0	1	40	45	15	47
Survation/Record 4-7.4.14	1006	4/4/14	Friday	4/7/14	Monday	4	1	0	0	0	1	37	47	16	44
ICM/SoS 14-16.4.14	1004	4/14/14	Monday	4/16/14	Wednesday	3	1	1	1	0	0	39	42	19	48
TNS BMRB 23.4-2.5.14	996	4/23/14	Wednesday	5/2/14	Friday	10	1	1	1	1	1	30	42	28	41
YouGov/Channel 4 25-28.4.14	1208	4/25/14	Friday	4/28/14	Monday	4	1	0	0	0	1	37	51	10	42
Progressive/Sunday Mail 7-8.5.14	1301	5/7/14	Wednesday	5/8/14	Thursday	2	0	0	1	1	0	34	54	12	39
Panelbase/Sunday Times 8-14.5.14	1046	5/8/14	Thursday	5/14/14	Wednesday	7	1	1	1	1	1	40	47	13	46
Survation/Record 9-12.5.14	1003	5/9/14	Friday	5/12/14	Monday	4	1	0	0	0	1	37	47	17	44
ICM/SoS 12-15.5.14	1003	5/12/14	Monday	5/15/14	Thursday	4	1	1	1	1	0	34	46	20	42
TNS BMRB 21-28.5.14	1011	5/21/14	Wednesday	5/28/14	Wednesday	8	1	1	1	1	1	30	42	28	41
Ipsos MORI/STV 26.5-1.6.14	1003	5/26/14	Monday	6/1/14	Sunday	7	1	1	1	1	1	36	54	10	40
Survation/Record 6-10.6.14	1004	6/6/14	Friday	6/10/14	Tuesday	5	1	1	0	0	1	39	44	17	47
ICM/Sos 9-12.6.14	1002	6/9/14	Monday	6/12/14	Thursday	4	1	1	1	1	0	36	43	21	45
Panelbase/Yes 9-11.6.14	1060	6/9/14	Monday	6/11/14	Wednesday	3	1	1	1	0	0	43	46	12	48
TNS BMRB/Open Dem 10-23.6.14	1004	6/10/14	Tuesday	6/23/14	Monday	14	1	1	0	1	1	32	46	22	41
YouGov/Sun 12-16.6.14	1039	6/12/14	Thursday	6/16/14	Monday	5	1	0	0	1	1	36	53	9	40
TNS BMRB 25.6-9.7.14	995	6/25/14	Wednesday	7/9/14	Wednesday	15	1	1	1	1	1	32	41	27	44
YouGov/Times 25-29.6.14	1206	6/25/14	Wednesday	6/29/14	Sunday	5	0	0	1	1	1	35	54	10	39
Survation/Record 4-8.7.14	1013	7/4/14	Friday	7/8/14	Tuesday	5	1	1	0	0	1	41	46	13	47
						9									
Total	81564										Mean	33.9	48.7	18.4	41.0

MONDAYS INCLUDED

Poll details	Number of responses	Start date	Start day	End date	End day	Total days	Monday?	Tuesday?	Wednesday?	Thursday?	Friday?	Yes%	No%	Don't Know%	Yes% (DK removed)
MORI; Times/ 4-9/2/13	1003	2/4/13	Monday	2/9/13	Saturday	6	1	1	1	1	1	34	55	11	38
TNS-BMRB; CND 20-28/2/13	1001	2/20/13	Wednesday	2/28/13	Thursday	9	1	0	1	1	1	33	52	15	39
Panelbase/Sunday Times 18-22/3/13	885	3/18/13	Monday	3/22/13	Friday	5	1	1	1	1	1	36	46	18	44
TNS-BMRB; Herald 20/3-2/4.13	1002	3/20/13	Wednesday	4/3/13	Wednesday	15	1	1	1	1	1	30	51	19	37
Survation/Sunday Post 13-15.4.14	1001	3/13/14	Thursday	4/15/14	Tuesday	34	1	1	1	1	1	38	46	16	45
ICM/SoS 14-16.4.14	1004	4/14/14	Monday	4/16/14	Wednesday	3	1	1	1	0	0	39	42	19	48
TNS BMRB 23.4-2.5.14	996	4/23/14	Wednesday	5/2/14	Friday	10	1	1	1	1	1	30	42	28	41
Ashcroft (YouGov)/ 29.4-2.5.13	1236	4/29/13	Monday	5/2/13	Thursday	4	1	1	1	1	0	30	56	12	35
MORI/Times 29.4-5.5.13	1001	4/29/13	Monday	5/5/13	Sunday	7	1	1	1	1	1	31	59	10	34
Ashcroft (ORB) 22.2.-9.5.13	10007	2/22/13	Friday	5/9/13	Thursday	77	1	1	1	1	1	26	65	10	29
Panelbase/Sunday Times 10-16/5/13	1004	5/10/13	Friday	5/16/13	Thursday	7	1	1	1	1	1	36	44	20	45
ICM/Scotland on Sunday 7-11.7.13	1002	7/7/13	Sunday	7/11/13	Thursday	5	1	1	1	1	0	34	45	21	43
Panelbase/Sunday Times 17-24/7/13	1001	7/17/13	Wednesday	7/24/13	Wednesday	8	1	1	1	1	1	37	46	17	45
YouGov/Devo Plus 19-22/8/13	1171	8/19/13	Monday	8/22/13	Thursday	4	1	1	1	1	0	29	59	12	33
TNS-BMRB : 21-27/8/13	1017	8/21/13	Wednesday	8/27/13	Tuesday	7	1	1	1	1	1	25	47	28	35
Panelbase/SNP 23-28/8/13	1043	8/23/13	Friday	8/28/13	Wednesday	6	1	1	1	0	1	44	43	13	51
Panelbase/Sunday Times 30.8-5.9.13	1002	8/30/13	Friday	9/5/13	Thursday	7	1	1	1	1	1	37	47	16	44
Ipsos MORI/STV 9-15.9.13	1000	9/9/13	Monday	9/15/13	Sunday	7	1	1	1	1	1	31	59	9	34
YouGov/Times 13-16.9.13	1139	9/13/13	Friday	9/16/13	Monday	4	1	0	0	0	1	32	52	15	38
Progressive/Mail online 13-16.9.13	1051	9/13/13	Friday	9/16/13	Monday	4	1	0	0	0	1	27	59	14	31
TNS/BMRB 25.9-2.10.13	1004	9/25/13	Wednesday	10/2/13	Wednesday	8	1	1	1	1	1	25	44	31	36
SSA 25.6-23.10.13	1497	6/25/13	Tuesday	10/23/13	Wednesday	121	1	1	1	1	1	30	54	16	36
Panelbase/Wings 17-24.10.13	866	10/17/13	Thursday	10/24/13	Thursday	8	1	1	1	1	1	37	45	17	45
TNS/BMRB 23-30.10.13	1010	10/23/13	Wednesday	10/30/13	Wednesday	8	1	1	1	1	1	25	43	32	37
Panelbase/Sunday Times 12-20.11.13	1006	11/12/13	Tuesday	11/20/13	Wednesday	9	1	1	1	1	1	38	47	15	45
TNS/BMRB 20-27.11.13	1004	11/20/13	Wednesday	11/27/13	Wednesday	8	1	1	1	1	1	26	42	32	38
YouGov (unpub) 27.11-2.12.13	1118	11/27/13	Wednesday	12/2/13	Monday	6	1	0	1	1	1	31	55	13	36
Ipsos MORI 29.11-5.12.13	1006	11/29/13	Friday	12/5/13	Thursday	7	1	1	1	1	1	34	57	10	37
YouGov/Times 6-9.12.13	1074	12/6/13	Friday	12/9/13	Monday	4	1	0	0	0	1	33	52	13	39
TNS BMRB 3-10.12.13	1055	12/3/13	Tuesday	12/10/13	Tuesday	8	1	1	1	1	1	27	41	33	39
TNS BMRB/Open Dem 14-20.1.14	1054	1/14/14	Tuesday	1/20/14	Monday	7	1	1	1	1	1	29	42	29	41
YouGov 21-27.1.14	1192	1/21/14	Tuesday	1/27/14	Monday	7	1	1	1	1	1	33	52	12	39
YouGov/Sun 3-5.2.14	1047	2/3/14	Monday	2/5/14	Wednesday	3	1	1	1	0	0	34	52	12	39
TNS BMRB 28.1-6.2.14	996	1/28/14	Tuesday	2/6/14	Thursday	10	1	1	1	1	1	29	42	29	41
Panelbase.Sun Times 29.1-7.2.14	1012	1/29/14	Wednesday	2/7/14	Friday	10	1	1	1	1	1	37	49	14	43
Survation/Mail 17-18.2.14	1005	2/17/14	Monday	2/18/14	Tuesday	2	1	1	0	0	0	38	47	16	45
ICM/SoS 17-21.2.14	1004	2/17/14	Monday	2/21/14	Friday	5	1	1	1	1	1	37	49	14	43
Ipsos MORI/STV 20-24.2.14	1001	2/20/14	Thursday	2/24/14	Monday	5	1	0	0	1	1	32	57	11	36
YouGov/Sun 24-28.2.14	1257	2/24/14	Monday	2/28/14	Friday	5	1	1	1	1	1	35	53	11	40
TNS BMRB 26.2-9.3.14	1019	2/26/14	Wednesday	3/14/14	Friday	17	1	1	1	1	1	28	42	30	40
Panelbase/Newsnet 7-14.3.14	1022	3/7/14	Friday	3/14/14	Friday	8	1	1	1	1	1	40	45	15	47
ICM/Sos 17-21.3.14	1010	3/7/14	Friday	3/21/14	Friday	15	1	1	1	1	1	39	46	15	45
YouGov/Times 20-24.3.14	1072	3/20/14	Thursday	3/24/14	Monday	5	1	0	0	1	1	37	52	10	42
TNS BMRB 21.3-2.4.14	988	3/21/14	Friday	4/2/14	Wednesday	13	1	1	1	1	1	29	41	30	41
Panelbase/Wings 28.3-4.4.14	1025	3/28/14	Friday	4/4/14	Friday	8	1	1	1	1	1	41	46	14	47
Survation/Record 4-7.4.14	1006	4/4/14	Friday	4/7/14	Monday	4	1	0	0	0	1	37	47	16	44
Panelbase/Yes 4-9.4.14	1024	4/4/14	Friday	4/9/14	Wednesday	6	1	1	1	0	1	40	45	15	47
YouGov/Channel 4 25-28.4.14	1208	4/25/14	Friday	4/28/14	Monday	4	1	0	0	0	1	37	51	10	42
Survation/Record 9-12.5.14	1003	5/9/14	Friday	5/12/14	Monday	4	1	0	0	0	1	37	47	17	44
Panelbase/Sunday Times 8-14.5.14	1046	5/8/14	Thursday	5/14/14	Wednesday	7	1	1	1	1	1	40	47	13	46
ICM/SoS 12-15.5.14	1003	5/12/14	Monday	5/15/14	Thursday	4	1	1	1	1	0	34	46	20	42
TNS BMRB 21-28.5.14	1011	5/21/14	Wednesday	5/28/14	Wednesday	8	1	1	1	1	1	30	42	28	41
Ipsos MORI/STV 26.5-1.6.14	1003	5/26/14	Monday	6/1/14	Sunday	7	1	1	1	1	1	36	54	10	40
Survation/Record 6-10.6.14	1004	6/6/14	Friday	6/10/14	Tuesday	5	1	1	0	0	1	39	44	17	47
Panelbase/Yes 9-11.6.14	1060	6/9/14	Monday	6/11/14	Wednesday	3	1	1	1	0	0	43	46	12	48
ICM/Sos 9-12.6.14	1002	6/9/14	Monday	6/12/14	Thursday	4	1	1	1	1	0	36	43	21	45
YouGov/Sun 12-16.6.14	1039	6/12/14	Thursday	6/16/14	Monday	5	1	0	0	1	1	36	53	9	40
TNS BMRB/Open Dem 10-23.6.14	1004	6/10/14	Tuesday	6/23/14	Monday	14	1	1	0	1	1	32	46	22	41
Survation/Record 4-8.7.14	1013	7/4/14	Friday	7/8/14	Tuesday	5	1	1	0	0	1	41	46	13	47
TNS BMRB 25.6-9.7.14	995	6/25/14	Wednesday	7/9/14	Wednesday	15	1	1	1	1	1	32	41	27	44
Total	71331										Mean	33.9	48.5	18.7	41.1

MONDAYS EXCLUDED

Poll details	Number of responses	Start date	Start day	End date	End day	Total days	Monday?	Tuesday?	Wednesday?	Thursday?	Friday?	Yes%	No%	Don't Know%	Yes% (DK removed)
Angus Reid; Mail on Sunday/ 30/1-1/2/13	1003	1/30/13	Wednesday	2/1/13	Friday	3	0	0	1	1	1	32	47	20	41
Angus Reid /Sunday Express 14-16/8/13	549	8/14/13	Wednesday	8/16/13	Friday	3	0	0	1	1	1	34	47	19	42
ICM/SoS/Scotsman 10-13.9.13	1002	9/10/13	Tuesday	9/13/13	Friday	4	0	1	1	1	1	32	49	19	40
Progressive/MoS 27-29.11.13	1134	11/27/13	Wednesday	11/29/13	Friday	3	0	0	1	1	1	27	56	17	33
ICM/SoS 21-24.1.14	1004	1/21/14	Tuesday	1/24/14	Friday	4	0	1	1	1	1	37	44	19	46
Survation/MoS 29-31.1.14	1010	1/29/14	Wednesday	1/31/14	Friday	3	0	0	1	1	1	32	52	16	38
Panelbase/SNP 18-21.2.14	1022	2/18/14	Tuesday	2/21/14	Friday	4	0	1	1	1	1	37	47	16	44
Survation/Record 6-7.3.14	1002	3/6/14	Thursday	3/7/14	Friday	2	0	0	0	1	1	39	48	13	45
Progressive/Sunday Mail 7-8.5.14	1301	5/7/14	Wednesday	5/8/14	Thursday	2	0	0	1	1	0	34	54	12	39
YouGov/Times 25-29.6.14	1206	6/25/14	Wednesday	6/29/14	Sunday	5	0	0	1	1	1	35	54	10	39
Total	10233										Mean	33.9	49.8	16	40.7

TUESDAYS INCLUDED

Poll details	Number of responses	Start date	Start day	End date	End day	Total days	Monday?	Tuesday?	Wednesday?	Thursday?	Friday?	Yes%	No%	Don't Know%	Yes% (DK removed)
MORI; Times/ 4-9/2/13	1003	2/4/13	Monday	2/9/13	Saturday	6	1	1	1	1	1	34	55	11	38
Panelbase/Sunday Times 18-22/3/13	885	3/18/13	Monday	3/22/13	Friday	5	1	1	1	1	1	36	46	18	44
TNS-BMRB; Herald 20/3-2/4.13	1002	3/20/13	Wednesday	4/3/13	Wednesday	15	1	1	1	1	1	30	51	19	37
Survation/Sunday Post 13-15.4.14	1001	3/13/14	Thursday	4/15/14	Tuesday	34	1	1	1	1	1	38	46	16	45
ICM/SoS 14-16.4.14	1004	4/14/14	Monday	4/16/14	Wednesday	3	1	1	1	0	0	39	42	19	48
TNS BMRB 23.4-2.5.14	996	4/23/14	Wednesday	5/2/14	Friday	10	1	1	1	1	1	30	42	28	41
Ashcroft (YouGov)/ 29.4-2.5.13	1236	4/29/13	Monday	5/2/13	Thursday	4	1	1	1	1	0	30	56	12	35
MORI/Times 29.4-5.5.13	1001	4/29/13	Monday	5/5/13	Sunday	7	1	1	1	1	1	31	59	10	34
Ashcroft (ORB) 22.2.-9.5.13	10007	2/22/13	Friday	5/9/13	Thursday	77	1	1	1	1	1	26	65	10	29
Panelbase/Sunday Times 10-16/5/13	1004	5/10/13	Friday	5/16/13	Thursday	7	1	1	1	1	1	36	44	20	45
ICM/Scotland on Sunday 7-11.7.13	1002	7/7/13	Sunday	7/11/13	Thursday	5	1	1	1	1	0	34	45	21	43
Panelbase/Sunday Times 17-24/7/13	1001	7/17/13	Wednesday	7/24/13	Wednesday	8	1	1	1	1	1	37	46	17	45
YouGov/Devo Plus 19-22/8/13	1171	8/19/13	Monday	8/22/13	Thursday	4	1	1	1	1	0	29	59	12	33
TNS-BMRB : 21-27/8/13	1017	8/21/13	Wednesday	8/27/13	Tuesday	7	1	1	1	1	1	25	47	28	35
Panelbase/SNP 23-28/8/13	1043	8/23/13	Friday	8/28/13	Wednesday	6	1	1	1	0	1	44	43	13	51
Panelbase/Sunday Times 30.8-5.9.13	1002	8/30/13	Friday	9/5/13	Thursday	7	1	1	1	1	1	37	47	16	44
Ipsos MORI/STV 9-15.9.13	1000	9/9/13	Monday	9/15/13	Sunday	7	1	1	1	1	1	31	59	9	34
TNS/BMRB 25.9-2.10.13	1004	9/25/13	Wednesday	10/2/13	Wednesday	8	1	1	1	1	1	25	44	31	36
SSA 25.6-23.10.13	1497	6/25/13	Tuesday	10/23/13	Wednesday	121	1	1	1	1	1	30	54	16	36
Panelbase/Wings 17-24.10.13	866	10/17/13	Thursday	10/24/13	Thursday	8	1	1	1	1	1	37	45	17	45
TNS/BMRB 23-30.10.13	1010	10/23/13	Wednesday	10/30/13	Wednesday	8	1	1	1	1	1	25	43	32	37
Panelbase/Sunday Times 12-20.11.13	1006	11/12/13	Tuesday	11/20/13	Wednesday	9	1	1	1	1	1	38	47	15	45
TNS/BMRB 20-27.11.13	1004	11/20/13	Wednesday	11/27/13	Wednesday	8	1	1	1	1	1	26	42	32	38
Ipsos MORI 29.11-5.12.13	1006	11/29/13	Friday	12/5/13	Thursday	7	1	1	1	1	1	34	57	10	37
TNS BMRB 3-10.12.13	1055	12/3/13	Tuesday	12/10/13	Tuesday	8	1	1	1	1	1	27	41	33	39
TNS BMRB/Open Dem 14-20.1.14	1054	1/14/14	Tuesday	1/20/14	Monday	7	1	1	1	1	1	29	42	29	41
YouGov 21-27.1.14	1192	1/21/14	Tuesday	1/27/14	Monday	7	1	1	1	1	1	33	52	12	39
YouGov/Sun 3-5.2.14	1047	2/3/14	Monday	2/5/14	Wednesday	3	1	1	1	0	0	34	52	12	39
TNS BMRB 28.1-6.2.14	996	1/28/14	Tuesday	2/6/14	Thursday	10	1	1	1	1	1	29	42	29	41
Panelbase.Sun Times 29.1-7.2.14	1012	1/29/14	Wednesday	2/7/14	Friday	10	1	1	1	1	1	37	49	14	43
Survation/Mail 17-18.2.14	1005	2/17/14	Monday	2/18/14	Tuesday	2	1	1	0	0	0	38	47	16	45
ICM/SoS 17-21.2.14	1004	2/17/14	Monday	2/21/14	Friday	5	1	1	1	1	1	37	49	14	43
YouGov/Sun 24-28.2.14	1257	2/24/14	Monday	2/28/14	Friday	5	1	1	1	1	1	35	53	11	40
TNS BMRB 26.2-9.3.14	1019	2/26/14	Wednesday	3/14/14	Friday	17	1	1	1	1	1	28	42	30	40
Panelbase/Newsnet 7-14.3.14	1022	3/7/14	Friday	3/14/14	Friday	8	1	1	1	1	1	40	45	15	47
ICM/Sos 17-21.3.14	1010	3/7/14	Friday	3/21/14	Friday	15	1	1	1	1	1	39	46	15	45
TNS BMRB 21.3-2.4.14	988	3/21/14	Friday	4/2/14	Wednesday	13	1	1	1	1	1	29	41	30	41
Panelbase/Wings 28.3-4.4.14	1025	3/28/14	Friday	4/4/14	Friday	8	1	1	1	1	1	41	46	14	47
Panelbase/Yes 4-9.4.14	1024	4/4/14	Friday	4/9/14	Wednesday	6	1	1	1	0	1	40	45	15	47
Panelbase/Sunday Times 8-14.5.14	1046	5/8/14	Thursday	5/14/14	Wednesday	7	1	1	1	1	1	40	47	13	46
ICM/SoS 12-15.5.14	1003	5/12/14	Monday	5/15/14	Thursday	4	1	1	1	1	0	34	46	20	42
TNS BMRB 21-28.5.14	1011	5/21/14	Wednesday	5/28/14	Wednesday	8	1	1	1	1	1	30	42	28	41
Ipsos MORI/STV 26.5-1.6.14	1003	5/26/14	Monday	6/1/14	Sunday	7	1	1	1	1	1	36	54	10	40
Survation/Record 6-10.6.14	1004	6/6/14	Friday	6/10/14	Tuesday	5	1	1	0	0	1	39	44	17	47
Panelbase/Yes 9-11.6.14	1060	6/9/14	Monday	6/11/14	Wednesday	3	1	1	1	0	0	43	46	12	48
ICM/Sos 9-12.6.14	1002	6/9/14	Monday	6/12/14	Thursday	4	1	1	1	1	0	36	43	21	45
TNS BMRB/Open Dem 10-23.6.14	1004	6/10/14	Tuesday	6/23/14	Monday	14	1	1	0	1	1	32	46	22	41
Survation/Record 4-8.7.14	1013	7/4/14	Friday	7/8/14	Tuesday	5	1	1	0	0	1	41	46	13	47
TNS BMRB 25.6-9.7.14	995	6/25/14	Wednesday	7/9/14	Wednesday	15	1	1	1	1	1	32	41	27	44
ICM/SoS/Scotsman 10-13.9.13	1002	9/10/13	Tuesday	9/13/13	Friday	4	0	1	1	1	1	32	49	19	40
ICM/SoS 21-24.1.14	1004	1/21/14	Tuesday	1/24/14	Friday	4	0	1	1	1	1	37	44	19	46
Panelbase/SNP 18-21.2.14	1022	2/18/14	Tuesday	2/21/14	Friday	4	0	1	1	1	1	37	47	16	44
Total	62647										Mean	34.0	47.5	19.1	41.6

TUESDAYS EXCLUDED

Poll details	Number of responses	Start date	Start day	End date	End day	Total days	Monday?	Tuesday?	Wednesday?	Thursday?	Friday?	Yes%	No%	Don't Know%	Yes% (DK removed)
Angus Reid; Mail on Sunday/ 30/1-1/2/13	1003	1/30/13	Wednesday	2/1/13	Friday	3	0	0	1	1	1	32	47	20	41
TNS-BMRB; CND 20-28/2/13	1001	2/20/13	Wednesday	2/28/13	Thursday	9	1	0	1	1	1	33	52	15	39
Angus Reid /Sunday Express 14-16/8/13	549	8/14/13	Wednesday	8/16/13	Friday	3	0	0	1	1	1	34	47	19	42
YouGov/Times 13-16.9.13	1139	9/13/13	Friday	9/16/13	Monday	4	1	0	0	0	1	32	52	15	38
Progressive/Mail online 13-16.9.13	1051	9/13/13	Friday	9/16/13	Monday	4	1	0	0	0	1	27	59	14	31
Progressive/MoS 27-29.11.13	1134	11/27/13	Wednesday	11/29/13	Friday	3	0	0	1	1	1	27	56	17	33
YouGov (unpub) 27.11-2.12.13	1118	11/27/13	Wednesday	12/2/13	Monday	6	1	0	1	1	1	31	55	13	36
YouGov/Times 6-9.12.13	1074	12/6/13	Friday	12/9/13	Monday	4	1	0	0	0	1	33	52	13	39
Survation/MoS 29-31.1.14	1010	1/29/14	Wednesday	1/31/14	Friday	3	0	0	1	1	1	32	52	16	38
Ipsos MORI/STV 20-24.2.14	1001	2/20/14	Thursday	2/24/14	Monday	5	1	0	0	1	1	32	57	11	36
Survation/Record 6-7.3.14	1002	3/6/14	Thursday	3/7/14	Friday	2	0	0	0	1	1	39	48	13	45
YouGov/Times 20-24.3.14	1072	3/20/14	Thursday	3/24/14	Monday	5	1	0	0	1	1	37	52	10	42
Survation/Record 4-7.4.14	1006	4/4/14	Friday	4/7/14	Monday	4	1	0	0	0	1	37	47	16	44
YouGov/Channel 4 25-28.4.14	1208	4/25/14	Friday	4/28/14	Monday	4	1	0	0	0	1	37	51	10	42
Progressive/Sunday Mail 7-8.5.14	1301	5/7/14	Wednesday	5/8/14	Thursday	2	0	0	1	1	0	34	54	12	39
Survation/Record 9-12.5.14	1003	5/9/14	Friday	5/12/14	Monday	4	1	0	0	0	1	37	47	17	44
YouGov/Sun 12-16.6.14	1039	6/12/14	Thursday	6/16/14	Monday	5	1	0	0	1	1	36	53	9	40
YouGov/Times 25-29.6.14	1206	6/25/14	Wednesday	6/29/14	Sunday	5	0	0	1	1	1	35	54	10	39
Total	18917										Mean	33.6	51.9	14.6	39.3

WEDNESDAYS INCLUDED

Poll details	Number of responses	Start date	Start day	End date	End day	Total days	Monday?	Tuesday?	Wednesday?	Thursday?	Friday?	Yes%	No%	Don't Know%	Yes% (DK removed)
MORI; Times/ 4-9/2/13	1003	2/4/13	Monday	2/9/13	Saturday	6	1	1	1	1	1	34	55	11	38
Panelbase/Sunday Times 18-22/3/13	885	3/18/13	Monday	3/22/13	Friday	5	1	1	1	1	1	36	46	18	44
TNS-BMRB; Herald 20/3-2/4.13	1002	3/20/13	Wednesday	4/3/13	Wednesday	15	1	1	1	1	1	30	51	19	37
Survation/Sunday Post 13-15.4.14	1001	3/13/14	Thursday	4/15/14	Tuesday	34	1	1	1	1	1	38	46	16	45
ICM/SoS 14-16.4.14	1004	4/14/14	Monday	4/16/14	Wednesday	3	1	1	1	0	0	39	42	19	48
TNS BMRB 23.4-2.5.14	996	4/23/14	Wednesday	5/2/14	Friday	10	1	1	1	1	1	30	42	28	41
Ashcroft (YouGov)/ 29.4-2.5.13	1236	4/29/13	Monday	5/2/13	Thursday	4	1	1	1	1	0	30	56	12	35
MORI/Times 29.4-5.5.13	1001	4/29/13	Monday	5/5/13	Sunday	7	1	1	1	1	1	31	59	10	34
Ashcroft (ORB) 22.2.-9.5.13	10007	2/22/13	Friday	5/9/13	Thursday	77	1	1	1	1	1	26	65	10	29
Panelbase/Sunday Times 10-16/5/13	1004	5/10/13	Friday	5/16/13	Thursday	7	1	1	1	1	1	36	44	20	45
ICM/Scotland on Sunday 7-11.7.13	1002	7/7/13	Sunday	7/11/13	Thursday	5	1	1	1	1	0	34	45	21	43
Panelbase/Sunday Times 17-24/7/13	1001	7/17/13	Wednesday	7/24/13	Wednesday	8	1	1	1	1	1	37	46	17	45
YouGov/Devo Plus 19-22/8/13	1171	8/19/13	Monday	8/22/13	Thursday	4	1	1	1	1	0	29	59	12	33
TNS-BMRB : 21-27/8/13	1017	8/21/13	Wednesday	8/27/13	Tuesday	7	1	1	1	1	1	25	47	28	35
Panelbase/SNP 23-28/8/13	1043	8/23/13	Friday	8/28/13	Wednesday	6	1	1	1	0	1	44	43	13	51
Panelbase/Sunday Times 30.8-5.9.13	1002	8/30/13	Friday	9/5/13	Thursday	7	1	1	1	1	1	37	47	16	44
Ipsos MORI/STV 9-15.9.13	1000	9/9/13	Monday	9/15/13	Sunday	7	1	1	1	1	1	31	59	9	34
TNS/BMRB 25.9-2.10.13	1004	9/25/13	Wednesday	10/2/13	Wednesday	8	1	1	1	1	1	25	44	31	36
SSA 25.6-23.10.13	1497	6/25/13	Tuesday	10/23/13	Wednesday	121	1	1	1	1	1	30	54	16	36
Panelbase/Wings 17-24.10.13	866	10/17/13	Thursday	10/24/13	Thursday	8	1	1	1	1	1	37	45	17	45
TNS/BMRB 23-30.10.13	1010	10/23/13	Wednesday	10/30/13	Wednesday	8	1	1	1	1	1	25	43	32	37
Panelbase/Sunday Times 12-20.11.13	1006	11/12/13	Tuesday	11/20/13	Wednesday	9	1	1	1	1	1	38	47	15	45
TNS/BMRB 20-27.11.13	1004	11/20/13	Wednesday	11/27/13	Wednesday	8	1	1	1	1	1	26	42	32	38
Ipsos MORI 29.11-5.12.13	1006	11/29/13	Friday	12/5/13	Thursday	7	1	1	1	1	1	34	57	10	37
TNS BMRB 3-10.12.13	1055	12/3/13	Tuesday	12/10/13	Tuesday	8	1	1	1	1	1	27	41	33	39
TNS BMRB/Open Dem 14-20.1.14	1054	1/14/14	Tuesday	1/20/14	Monday	7	1	1	1	1	1	29	42	29	41
YouGov 21-27.1.14	1192	1/21/14	Tuesday	1/27/14	Monday	7	1	1	1	1	1	33	52	12	39
YouGov/Sun 3-5.2.14	1047	2/3/14	Monday	2/5/14	Wednesday	3	1	1	1	0	0	34	52	12	39
TNS BMRB 28.1-6.2.14	996	1/28/14	Tuesday	2/6/14	Thursday	10	1	1	1	1	1	29	42	29	41
Panelbase.Sun Times 29.1-7.2.14	1012	1/29/14	Wednesday	2/7/14	Friday	10	1	1	1	1	1	37	49	14	43
ICM/SoS 17-21.2.14	1004	2/17/14	Monday	2/21/14	Friday	5	1	1	1	1	1	37	49	14	43
YouGov/Sun 24-28.2.14	1257	2/24/14	Monday	2/28/14	Friday	5	1	1	1	1	1	35	53	11	40
TNS BMRB 26.2-9.3.14	1019	2/26/14	Wednesday	3/14/14	Friday	17	1	1	1	1	1	28	42	30	40
Panelbase/Newsnet 7-14.3.14	1022	3/7/14	Friday	3/14/14	Friday	8	1	1	1	1	1	40	45	15	47
ICM/Sos 17-21.3.14	1010	3/7/14	Friday	3/21/14	Friday	15	1	1	1	1	1	39	46	15	45
TNS BMRB 21.3-2.4.14	988	3/21/14	Friday	4/2/14	Wednesday	13	1	1	1	1	1	29	41	30	41
Panelbase/Wings 28.3-4.4.14	1025	3/28/14	Friday	4/4/14	Friday	8	1	1	1	1	1	41	46	14	47
Panelbase/Yes 4-9.4.14	1024	4/4/14	Friday	4/9/14	Wednesday	6	1	1	1	0	1	40	45	15	47
Panelbase/Sunday Times 8-14.5.14	1046	5/8/14	Thursday	5/14/14	Wednesday	7	1	1	1	1	1	40	47	13	46
ICM/SoS 12-15.5.14	1003	5/12/14	Monday	5/15/14	Thursday	4	1	1	1	1	0	34	46	20	42
TNS BMRB 21-28.5.14	1011	5/21/14	Wednesday	5/28/14	Wednesday	8	1	1	1	1	1	30	42	28	41
Ipsos MORI/STV 26.5-1.6.14	1003	5/26/14	Monday	6/1/14	Sunday	7	1	1	1	1	1	36	54	10	40
Panelbase/Yes 9-11.6.14	1060	6/9/14	Monday	6/11/14	Wednesday	3	1	1	1	0	0	43	46	12	48
ICM/Sos 9-12.6.14	1002	6/9/14	Monday	6/12/14	Thursday	4	1	1	1	1	0	36	43	21	45
TNS BMRB 25.6-9.7.14	995	6/25/14	Wednesday	7/9/14	Wednesday	15	1	1	1	1	1	32	41	27	44
ICM/SoS/Scotsman 10-13.9.13	1002	9/10/13	Tuesday	9/13/13	Friday	4	0	1	1	1	1	32	49	19	40
ICM/SoS 21-24.1.14	1004	1/21/14	Tuesday	1/24/14	Friday	4	0	1	1	1	1	37	44	19	46
Panelbase/SNP 18-21.2.14	1022	2/18/14	Tuesday	2/21/14	Friday	4	0	1	1	1	1	37	47	16	44
TNS-BMRB; CND 20-28/2/13	1001	2/20/13	Wednesday	2/28/13	Thursday	9	1	0	1	1	1	33	52	15	39
YouGov (unpub) 27.11-2.12.13	1118	11/27/13	Wednesday	12/2/13	Monday	6	1	0	1	1	1	31	55	13	36
Angus Reid; Mail on Sunday/ 30/1-1/2/13	1003	1/30/13	Wednesday	2/1/13	Friday	3	0	0	1	1	1	32	47	20	41
Angus Reid /Sunday Express 14-16/8/13	549	8/14/13	Wednesday	8/16/13	Friday	3	0	0	1	1	1	34	47	19	42
Progressive/MoS 27-29.11.13	1134	11/27/13	Wednesday	11/29/13	Friday	3	0	0	1	1	1	27	56	17	33
Survation/MoS 29-31.1.14	1010	1/29/14	Wednesday	1/31/14	Friday	3	0	0	1	1	1	32	52	16	38
Progressive/Sunday Mail 7-8.5.14	1301	5/7/14	Wednesday	5/8/14	Thursday	2	0	0	1	1	0	34	54	12	39
YouGov/Times 25-29.6.14	1206	6/25/14	Wednesday	6/29/14	Sunday	5	0	0	1	1	1	35	54	10	39
Total	66943										Mean	33.5	48.3	19.0	40.9

WEDNESDAYS EXCLUDED

Poll details	Number of responses	Start date	Start day	End date	End day	Total days	Monday?	Tuesday?	Wednesday?	Thursday?	Friday?	Yes%	No%	Don't Know%	Yes% (DK removed)
YouGov/Times 13-16.9.13	1139	9/13/13	Friday	9/16/13	Monday	4	1	0	0	0	1	32	52	15	38
Progressive/Mail online 13-16.9.13	1051	9/13/13	Friday	9/16/13	Monday	4	1	0	0	0	1	27	59	14	31
YouGov/Times 6-9.12.13	1074	12/6/13	Friday	12/9/13	Monday	4	1	0	0	0	1	33	52	13	39
Survation/Mail 17-18.2.14	1005	2/17/14	Monday	2/18/14	Tuesday	2	1	1	0	0	0	38	47	16	45
Ipsos MORI/STV 20-24.2.14	1001	2/20/14	Thursday	2/24/14	Monday	5	1	0	0	1	1	32	57	11	36
Survation/Record 6-7.3.14	1002	3/6/14	Thursday	3/7/14	Friday	2	0	0	0	1	1	39	48	13	45
YouGov/Times 20-24.3.14	1072	3/20/14	Thursday	3/24/14	Monday	5	1	0	0	1	1	37	52	10	42
Survation/Record 4-7.4.14	1006	4/4/14	Friday	4/7/14	Monday	4	1	0	0	0	1	37	47	16	44
YouGov/Channel 4 25-28.4.14	1208	4/25/14	Friday	4/28/14	Monday	4	1	0	0	0	1	37	51	10	42
Survation/Record 9-12.5.14	1003	5/9/14	Friday	5/12/14	Monday	4	1	0	0	0	1	37	47	17	44
Survation/Record 6-10.6.14	1004	6/6/14	Friday	6/10/14	Tuesday	5	1	1	0	0	1	39	44	17	47
YouGov/Sun 12-16.6.14	1039	6/12/14	Thursday	6/16/14	Monday	5	1	0	0	1	1	36	53	9	40
TNS BMRB/Open Dem 10-23.6.14	1004	6/10/14	Tuesday	6/23/14	Monday	14	1	1	0	1	1	32	46	22	41
Survation/Record 4-8.7.14	1013	7/4/14	Friday	7/8/14	Tuesday	5	1	1	0	0	1	41	46	13	47
Total	14621										Mean	35.5	50.1	15.4	41.5

THURSDAYS INCLUDED

Poll details	Number of responses	Start date	Start day	End date	End day	Total days	Monday?	Tuesday?	Wednesday?	Thursday?	Friday?	Yes%	No%	Don't Know%	Yes% (DK removed)
MORI; Times/ 4-9/2/13	1003	2/4/13	Monday	2/9/13	Saturday	6	1	1	1	1	1	34	55	11	38
Panelbase/Sunday Times 18-22/3/13	885	3/18/13	Monday	3/22/13	Friday	5	1	1	1	1	1	36	46	18	44
TNS-BMRB; Herald 20/3-2/4.13	1002	3/20/13	Wednesday	4/3/13	Wednesday	15	1	1	1	1	1	30	51	19	37
Survation/Sunday Post 13-15.4.14	1001	3/13/14	Thursday	4/15/14	Tuesday	34	1	1	1	1	1	38	46	16	45
TNS BMRB 23.4-2.5.14	996	4/23/14	Wednesday	5/2/14	Friday	10	1	1	1	1	1	30	42	28	41
Ashcroft (YouGov)/ 29.4-2.5.13	1236	4/29/13	Monday	5/2/13	Thursday	4	1	1	1	1	0	30	56	12	35
MORI/Times 29.4-5.5.13	1001	4/29/13	Monday	5/5/13	Sunday	7	1	1	1	1	1	31	59	10	34
Ashcroft (ORB) 22.2.-9.5.13	10007	2/22/13	Friday	5/9/13	Thursday	77	1	1	1	1	1	26	65	10	29
Panelbase/Sunday Times 10-16/5/13	1004	5/10/13	Friday	5/16/13	Thursday	7	1	1	1	1	1	36	44	20	45
ICM/Scotland on Sunday 7-11.7.13	1002	7/7/13	Sunday	7/11/13	Thursday	5	1	1	1	1	0	34	45	21	43
Panelbase/Sunday Times 17-24/7/13	1001	7/17/13	Wednesday	7/24/13	Wednesday	8	1	1	1	1	1	37	46	17	45
YouGov/Devo Plus 19-22/8/13	1171	8/19/13	Monday	8/22/13	Thursday	4	1	1	1	1	0	29	59	12	33
TNS-BMRB : 21-27/8/13	1017	8/21/13	Wednesday	8/27/13	Tuesday	7	1	1	1	1	1	25	47	28	35
Panelbase/Sunday Times 30.8-5.9.13	1002	8/30/13	Friday	9/5/13	Thursday	7	1	1	1	1	1	37	47	16	44
Ipsos MORI/STV 9-15.9.13	1000	9/9/13	Monday	9/15/13	Sunday	7	1	1	1	1	1	31	59	9	34
TNS/BMRB 25.9-2.10.13	1004	9/25/13	Wednesday	10/2/13	Wednesday	8	1	1	1	1	1	25	44	31	36
SSA 25.6-23.10.13	1497	6/25/13	Tuesday	10/23/13	Wednesday	121	1	1	1	1	1	30	54	16	36
Panelbase/Wings 17-24.10.13	866	10/17/13	Thursday	10/24/13	Thursday	8	1	1	1	1	1	37	45	17	45
TNS/BMRB 23-30.10.13	1010	10/23/13	Wednesday	10/30/13	Wednesday	8	1	1	1	1	1	25	43	32	37
Panelbase/Sunday Times 12-20.11.13	1006	11/12/13	Tuesday	11/20/13	Wednesday	9	1	1	1	1	1	38	47	15	45
TNS/BMRB 20-27.11.13	1004	11/20/13	Wednesday	11/27/13	Wednesday	8	1	1	1	1	1	26	42	32	38
Ipsos MORI 29.11-5.12.13	1006	11/29/13	Friday	12/5/13	Thursday	7	1	1	1	1	1	34	57	10	37
TNS BMRB 3-10.12.13	1055	12/3/13	Tuesday	12/10/13	Tuesday	8	1	1	1	1	1	27	41	33	39
TNS BMRB/Open Dem 14-20.1.14	1054	1/14/14	Tuesday	1/20/14	Monday	7	1	1	1	1	1	29	42	29	41
YouGov 21-27.1.14	1192	1/21/14	Tuesday	1/27/14	Monday	7	1	1	1	1	1	33	52	12	39
TNS BMRB 28.1-6.2.14	996	1/28/14	Tuesday	2/6/14	Thursday	10	1	1	1	1	1	29	42	29	41
Panelbase.Sun Times 29.1-7.2.14	1012	1/29/14	Wednesday	2/7/14	Friday	10	1	1	1	1	1	37	49	14	43
ICM/SoS 17-21.2.14	1004	2/17/14	Monday	2/21/14	Friday	5	1	1	1	1	1	37	49	14	43
YouGov/Sun 24-28.2.14	1257	2/24/14	Monday	2/28/14	Friday	5	1	1	1	1	1	35	53	11	40
TNS BMRB 26.2-9.3.14	1019	2/26/14	Wednesday	3/14/14	Friday	17	1	1	1	1	1	28	42	30	40
Panelbase/Newsnet 7-14.3.14	1022	3/7/14	Friday	3/14/14	Friday	8	1	1	1	1	1	40	45	15	47
ICM/Sos 17-21.3.14	1010	3/7/14	Friday	3/21/14	Friday	15	1	1	1	1	1	39	46	15	45
TNS BMRB 21.3-2.4.14	988	3/21/14	Friday	4/2/14	Wednesday	13	1	1	1	1	1	29	41	30	41
Panelbase/Wings 28.3-4.4.14	1025	3/28/14	Friday	4/4/14	Friday	8	1	1	1	1	1	41	46	14	47
Panelbase/Sunday Times 8-14.5.14	1046	5/8/14	Thursday	5/14/14	Wednesday	7	1	1	1	1	1	40	47	13	46
ICM/SoS 12-15.5.14	1003	5/12/14	Monday	5/15/14	Thursday	4	1	1	1	1	0	34	46	20	42
TNS BMRB 21-28.5.14	1011	5/21/14	Wednesday	5/28/14	Wednesday	8	1	1	1	1	1	30	42	28	41
Ipsos MORI/STV 26.5-1.6.14	1003	5/26/14	Monday	6/1/14	Sunday	7	1	1	1	1	1	36	54	10	40
ICM/Sos 9-12.6.14	1002	6/9/14	Monday	6/12/14	Thursday	4	1	1	1	1	0	36	43	21	45
TNS BMRB 25.6-9.7.14	995	6/25/14	Wednesday	7/9/14	Wednesday	15	1	1	1	1	1	32	41	27	44
ICM/SoS/Scotsman 10-13.9.13	1002	9/10/13	Tuesday	9/13/13	Friday	4	0	1	1	1	1	32	49	19	40
ICM/SoS 21-24.1.14	1004	1/21/14	Tuesday	1/24/14	Friday	4	0	1	1	1	1	37	44	19	46
Panelbase/SNP 18-21.2.14	1022	2/18/14	Tuesday	2/21/14	Friday	4	0	1	1	1	1	37	47	16	44
TNS-BMRB; CND 20-28/2/13	1001	2/20/13	Wednesday	2/28/13	Thursday	9	1	0	1	1	1	33	52	15	39
YouGov (unpub) 27.11-2.12.13	1118	11/27/13	Wednesday	12/2/13	Monday	6	1	0	1	1	1	31	55	13	36
Angus Reid; Mail on Sunday/ 30/1-1/2/13	1003	1/30/13	Wednesday	2/1/13	Friday	3	0	0	1	1	1	32	47	20	41
Angus Reid /Sunday Express 14-16/8/13	549	8/14/13	Wednesday	8/16/13	Friday	3	0	0	1	1	1	34	47	19	42
Progressive/MoS 27-29.11.13	1134	11/27/13	Wednesday	11/29/13	Friday	3	0	0	1	1	1	27	56	17	33
Survation/MoS 29-31.1.14	1010	1/29/14	Wednesday	1/31/14	Friday	3	0	0	1	1	1	32	52	16	38
Progressive/Sunday Mail 7-8.5.14	1301	5/7/14	Wednesday	5/8/14	Thursday	2	0	0	1	1	0	34	54	12	39
YouGov/Times 25-29.6.14	1206	6/25/14	Wednesday	6/29/14	Sunday	5	0	0	1	1	1	35	54	10	39
TNS BMRB/Open Dem 10-23.6.14	1004	6/10/14	Tuesday	6/23/14	Monday	14	1	1	0	1	1	32	46	22	41
Ipsos MORI/STV 20-24.2.14	1001	2/20/14	Thursday	2/24/14	Monday	5	1	0	0	1	1	32	57	11	36
YouGov/Times 20-24.3.14	1072	3/20/14	Thursday	3/24/14	Monday	5	1	0	0	1	1	37	52	10	42
YouGov/Sun 12-16.6.14	1039	6/12/14	Thursday	6/16/14	Monday	5	1	0	0	1	1	36	53	9	40
Survation/Record 6-7.3.14	1002	3/6/14	Thursday	3/7/14	Friday	2	0	0	0	1	1	39	48	13	45
Total	66883										Mean	33.1	48.8	19.1	40.4

THURSDAYS EXCLUDED

Poll details	Number of responses	Start date	Start day	End date	End day	Total days	Monday?	Tuesday?	Wednesday?	Thursday?	Friday?	Yes%	No%	Don't Know%	Yes% (DK removed)
ICM/SoS 14-16.4.14	1004	4/14/14	Monday	4/16/14	Wednesday	3	1	1	1	0	0	39	42	19	48
Panelbase/SNP 23-28/8/13	1043	8/23/13	Friday	8/28/13	Wednesday	6	1	1	1	0	1	44	43	13	51
YouGov/Times 13-16.9.13	1139	9/13/13	Friday	9/16/13	Monday	4	1	0	0	0	1	32	52	15	38
Progressive/Mail online 13-16.9.13	1051	9/13/13	Friday	9/16/13	Monday	4	1	0	0	0	1	27	59	14	31
YouGov/Times 6-9.12.13	1074	12/6/13	Friday	12/9/13	Monday	4	1	0	0	0	1	33	52	13	39
YouGov/Sun 3-5.2.14	1047	2/3/14	Monday	2/5/14	Wednesday	3	1	1	1	0	0	34	52	12	39
Survation/Mail 17-18.2.14	1005	2/17/14	Monday	2/18/14	Tuesday	2	1	1	0	0	0	38	47	16	45
Survation/Record 4-7.4.14	1006	4/4/14	Friday	4/7/14	Monday	4	1	0	0	0	1	37	47	16	44
Panelbase/Yes 4-9.4.14	1024	4/4/14	Friday	4/9/14	Wednesday	6	1	1	1	0	1	40	45	15	47
YouGov/Channel 4 25-28.4.14	1208	4/25/14	Friday	4/28/14	Monday	4	1	0	0	0	1	37	51	10	42
Survation/Record 9-12.5.14	1003	5/9/14	Friday	5/12/14	Monday	4	1	0	0	0	1	37	47	17	44
Survation/Record 6-10.6.14	1004	6/6/14	Friday	6/10/14	Tuesday	5	1	1	0	0	1	39	44	17	47
Panelbase/Yes 9-11.6.14	1060	6/9/14	Monday	6/11/14	Wednesday	3	1	1	1	0	0	43	46	12	48
Survation/Record 4-8.7.14	1013	7/4/14	Friday	7/8/14	Tuesday	5	1	1	0	0	1	41	46	13	47
Total	14681										Mean	37.2	48.1	15.2	43.6

FRIDAYS INCLUDED

Poll details	Number of responses	Start date	Start day	End date	End day	Total days	Monday?	Tuesday?	Wednesday?	Thursday?	Friday?	Yes%	No%	Don't Know%	Yes% (DK removed)
MORI; Times/ 4-9/2/13	1003	2/4/13	Monday	2/9/13	Saturday	6	1	1	1	1	1	34	55	11	38
Panelbase/Sunday Times 18-22/3/13	885	3/18/13	Monday	3/22/13	Friday	5	1	1	1	1	1	36	46	18	44
TNS-BMRB; Herald 20/3-2/4.13	1002	3/20/13	Wednesday	4/3/13	Wednesday	15	1	1	1	1	1	30	51	19	37
Survation/Sunday Post 13-15.4.14	1001	3/13/14	Thursday	4/15/14	Tuesday	34	1	1	1	1	1	38	46	16	45
TNS BMRB 23.4-2.5.14	996	4/23/14	Wednesday	5/2/14	Friday	10	1	1	1	1	1	30	42	28	41
MORI/Times 29.4-5.5.13	1001	4/29/13	Monday	5/5/13	Sunday	7	1	1	1	1	1	31	59	10	34
Ashcroft (ORB) 22.2.-9.5.13	10007	2/22/13	Friday	5/9/13	Thursday	77	1	1	1	1	1	26	65	10	29
Panelbase/Sunday Times 10-16/5/13	1004	5/10/13	Friday	5/16/13	Thursday	7	1	1	1	1	1	36	44	20	45
Panelbase/Sunday Times 17-24/7/13	1001	7/17/13	Wednesday	7/24/13	Wednesday	8	1	1	1	1	1	37	46	17	45
TNS-BMRB : 21-27/8/13	1017	8/21/13	Wednesday	8/27/13	Tuesday	7	1	1	1	1	1	25	47	28	35
Panelbase/Sunday Times 30.8-5.9.13	1002	8/30/13	Friday	9/5/13	Thursday	7	1	1	1	1	1	37	47	16	44
Ipsos MORI/STV 9-15.9.13	1000	9/9/13	Monday	9/15/13	Sunday	7	1	1	1	1	1	31	59	9	34
TNS/BMRB 25.9-2.10.13	1004	9/25/13	Wednesday	10/2/13	Wednesday	8	1	1	1	1	1	25	44	31	36
SSA 25.6-23.10.13	1497	6/25/13	Tuesday	10/23/13	Wednesday	121	1	1	1	1	1	30	54	16	36
Panelbase/Wings 17-24.10.13	866	10/17/13	Thursday	10/24/13	Thursday	8	1	1	1	1	1	37	45	17	45
TNS/BMRB 23-30.10.13	1010	10/23/13	Wednesday	10/30/13	Wednesday	8	1	1	1	1	1	25	43	32	37
Panelbase/Sunday Times 12-20.11.13	1006	11/12/13	Tuesday	11/20/13	Wednesday	9	1	1	1	1	1	38	47	15	45
TNS/BMRB 20-27.11.13	1004	11/20/13	Wednesday	11/27/13	Wednesday	8	1	1	1	1	1	26	42	32	38
Ipsos MORI 29.11-5.12.13	1006	11/29/13	Friday	12/5/13	Thursday	7	1	1	1	1	1	34	57	10	37
TNS BMRB 3-10.12.13	1055	12/3/13	Tuesday	12/10/13	Tuesday	8	1	1	1	1	1	27	41	33	39
TNS BMRB/Open Dem 14-20.1.14	1054	1/14/14	Tuesday	1/20/14	Monday	7	1	1	1	1	1	29	42	29	41
YouGov 21-27.1.14	1192	1/21/14	Tuesday	1/27/14	Monday	7	1	1	1	1	1	33	52	12	39
TNS BMRB 28.1-6.2.14	996	1/28/14	Tuesday	2/6/14	Thursday	10	1	1	1	1	1	29	42	29	41
Panelbase.Sun Times 29.1-7.2.14	1012	1/29/14	Wednesday	2/7/14	Friday	10	1	1	1	1	1	37	49	14	43
ICM/SoS 17-21.2.14	1004	2/17/14	Monday	2/21/14	Friday	5	1	1	1	1	1	37	49	14	43
YouGov/Sun 24-28.2.14	1257	2/24/14	Monday	2/28/14	Friday	5	1	1	1	1	1	35	53	11	40
TNS BMRB 26.2-9.3.14	1019	2/26/14	Wednesday	3/14/14	Friday	17	1	1	1	1	1	28	42	30	40
Panelbase/Newsnet 7-14.3.14	1022	3/7/14	Friday	3/14/14	Friday	8	1	1	1	1	1	40	45	15	47
ICM/Sos 17-21.3.14	1010	3/7/14	Friday	3/21/14	Friday	15	1	1	1	1	1	39	46	15	45
TNS BMRB 21.3-2.4.14	988	3/21/14	Friday	4/2/14	Wednesday	13	1	1	1	1	1	29	41	30	41
Panelbase/Wings 28.3-4.4.14	1025	3/28/14	Friday	4/4/14	Friday	8	1	1	1	1	1	41	46	14	47
Panelbase/Sunday Times 8-14.5.14	1046	5/8/14	Thursday	5/14/14	Wednesday	7	1	1	1	1	1	40	47	13	46
TNS BMRB 21-28.5.14	1011	5/21/14	Wednesday	5/28/14	Wednesday	8	1	1	1	1	1	30	42	28	41
Ipsos MORI/STV 26.5-1.6.14	1003	5/26/14	Monday	6/1/14	Sunday	7	1	1	1	1	1	36	54	10	40
TNS BMRB 25.6-9.7.14	995	6/25/14	Wednesday	7/9/14	Wednesday	15	1	1	1	1	1	32	41	27	44
ICM/SoS/Scotsman 10-13.9.13	1002	9/10/13	Tuesday	9/13/13	Friday	4	0	1	1	1	1	32	49	19	40
ICM/SoS 21-24.1.14	1004	1/21/14	Tuesday	1/24/14	Friday	4	0	1	1	1	1	37	44	19	46
Panelbase/SNP 18-21.2.14	1022	2/18/14	Tuesday	2/21/14	Friday	4	0	1	1	1	1	37	47	16	44
TNS-BMRB; CND 20-28/2/13	1001	2/20/13	Wednesday	2/28/13	Thursday	9	1	0	1	1	1	33	52	15	39
YouGov (unpub) 27.11-2.12.13	1118	11/27/13	Wednesday	12/2/13	Monday	6	1	0	1	1	1	31	55	13	36
Angus Reid; Mail on Sunday/ 30/1-1/2/13	1003	1/30/13	Wednesday	2/1/13	Friday	3	0	0	1	1	1	32	47	20	41
Angus Reid /Sunday Express 14-16/8/13	549	8/14/13	Wednesday	8/16/13	Friday	3	0	0	1	1	1	34	47	19	42
Progressive/MoS 27-29.11.13	1134	11/27/13	Wednesday	11/29/13	Friday	3	0	0	1	1	1	27	56	17	33
Survation/MoS 29-31.1.14	1010	1/29/14	Wednesday	1/31/14	Friday	3	0	0	1	1	1	32	52	16	38
YouGov/Times 25-29.6.14	1206	6/25/14	Wednesday	6/29/14	Sunday	5	0	0	1	1	1	35	54	10	39
TNS BMRB/Open Dem 10-23.6.14	1004	6/10/14	Tuesday	6/23/14	Monday	14	1	1	0	1	1	32	46	22	41
Ipsos MORI/STV 20-24.2.14	1001	2/20/14	Thursday	2/24/14	Monday	5	1	0	0	1	1	32	57	11	36
YouGov/Times 20-24.3.14	1072	3/20/14	Thursday	3/24/14	Monday	5	1	0	0	1	1	37	52	10	42
YouGov/Sun 12-16.6.14	1039	6/12/14	Thursday	6/16/14	Monday	5	1	0	0	1	1	36	53	9	40
Survation/Record 6-7.3.14	1002	3/6/14	Thursday	3/7/14	Friday	2	0	0	0	1	1	39	48	13	45
Panelbase/SNP 23-28/8/13	1043	8/23/13	Friday	8/28/13	Wednesday	6	1	1	1	0	1	44	43	13	51
Panelbase/Yes 4-9.4.14	1024	4/4/14	Friday	4/9/14	Wednesday	6	1	1	1	0	1	40	45	15	47
Survation/Record 6-10.6.14	1004	6/6/14	Friday	6/10/14	Tuesday	5	1	1	0	0	1	39	44	17	47
Survation/Record 4-8.7.14	1013	7/4/14	Friday	7/8/14	Tuesday	5	1	1	0	0	1	41	46	13	47
YouGov/Times 13-16.9.13	1139	9/13/13	Friday	9/16/13	Monday	4	1	0	0	0	1	32	52	15	38
Progressive/Mail online 13-16.9.13	1051	9/13/13	Friday	9/16/13	Monday	4	1	0	0	0	1	27	59	14	31
YouGov/Times 6-9.12.13	1074	12/6/13	Friday	12/9/13	Monday	4	1	0	0	0	1	33	52	13	39
Survation/Record 4-7.4.14	1006	4/4/14	Friday	4/7/14	Monday	4	1	0	0	0	1	37	47	16	44
YouGov/Channel 4 25-28.4.14	1208	4/25/14	Friday	4/28/14	Monday	4	1	0	0	0	1	37	51	10	42
Survation/Record 9-12.5.14	1003	5/9/14	Friday	5/12/14	Monday	4	1	0	0	0	1	37	47	17	44
Total	70733										Mean	33.7	48.6	18.6	40.9

FRIDAYS EXCLUDED

Poll details	Number of responses	Start date	Start day	End date	End day	Total days	Monday?	Tuesday?	Wednesday?	Thursday?	Friday?	Yes%	No%	Don't Know%	Yes% (DK removed)
ICM/SoS 14-16.4.14	1004	4/14/14	Monday	4/16/14	Wednesday	3	1	1	1	0	0	39	42	19	48
Ashcroft (YouGov)/ 29.4-2.5.13	1236	4/29/13	Monday	5/2/13	Thursday	4	1	1	1	1	0	30	56	12	35
ICM/Scotland on Sunday 7-11.7.13	1002	7/7/13	Sunday	7/11/13	Thursday	5	1	1	1	1	0	34	45	21	43
YouGov/Devo Plus 19-22/8/13	1171	8/19/13	Monday	8/22/13	Thursday	4	1	1	1	1	0	29	59	12	33
YouGov/Sun 3-5.2.14	1047	2/3/14	Monday	2/5/14	Wednesday	3	1	1	1	0	0	34	52	12	39
Survation/Mail 17-18.2.14	1005	2/17/14	Monday	2/18/14	Tuesday	2	1	1	0	0	0	38	47	16	45
Progressive/Sunday Mail 7-8.5.14	1301	5/7/14	Wednesday	5/8/14	Thursday	2	0	0	1	1	0	34	54	12	39
ICM/SoS 12-15.5.14	1003	5/12/14	Monday	5/15/14	Thursday	4	1	1	1	1	0	34	46	20	42
Panelbase/Yes 9-11.6.14	1060	6/9/14	Monday	6/11/14	Wednesday	3	1	1	1	0	0	43	46	12	48
ICM/Sos 9-12.6.14	1002	6/9/14	Monday	6/12/14	Thursday	4	1	1	1	1	0	36	43	21	45
Total	10831										Mean	35.1	49.0	17.3	41.7
