# Supplementary material for: Weekly Fluctuations in Risk Tolerance and Voting Behaviour
Source: PLoS One. 2016 Jul 8;11(7):e0159017. doi: 10.1371/journal.pone.0159017 (PMC4938543; doi:10.1371/journal.pone.0159017)
Supplement: S6 Table — Don’t Know responses are removed (9) to allow direct comparison with the actual referendum outcome. Rows denote intention polls, columns denote response numbers, start and end date and day, which weekday was included and poll outcomes. (PDF) [file pone.0159017.s006.pdf]

S6 Table. Data from the twenty opinion polls that were conducted during the purdah period. *Don't Know* responses are removed (9) to allow direct comparison with the actual referendum outcome. Rows denote intention polls, columns denote response numbers, start and end date and day, which weekday was included and poll outcomes.

Poll details	Number of responses	End date	Yes%	No%	Don't Know	Yes% (DK removed)
Survation/Daily Mail	1001	28/08/14	42	48	11	47
YouGov/The Times; The Sun	1063	01/09/14	42	48	10	47
Panelbase/Yes Scotland	1042	04/09/14	44	48	8	48
TNS-BMRB/Self-Funded or Unknown	990	04/09/14	38	39	23	59
YouGov/The Sunday Times	1084	05/09/14	47	45	7	51
Survation/Daily Record	1000	09/09/14	42	48	10	47
YouGov/The Times; The Sun	1268	11/09/14	45	50	6	47
ICM/The Guardian	1000	11/09/14	40	42	17	49
Opinium/The Observer	1055	11/09/14	45	49	6	48
Survation/Better Together	927	12/09/14	42	49	9	46
ICM/The Sunday Telegraph	705	12/09/14	49	42	9	54
Panelbase/The Sunday Times	1014	14/09/14	46	47	7	49
Opinium/Daily Telegraph	1156	15/09/14	45	49	6	48
ICM/Scotsman	1175	16/09/14	41	45	14	48
Survation/Daily Mail	1000	16/09/14	44	48	8	48
Ipsos Mori/STV	1405	16/09/14	47	49	5	49
Panelbase/Self-Funded or Unknown	1004	17/09/14	45	50	5	47
Survation/Daily Record	1266	17/09/14	43	48	9	47
YouGov/The Times; The Sun	3237	17/09/14	45	50	6	47
Ipsos Mori/London Evening Standard	991	17/09/14	45	50	4	47
Average (Purdah)	23383		43.9	47.2	9.0	48.7
Actual						44.7
Difference						-4.0
