# Supplementary material for: Weekly Fluctuations in Risk Tolerance and Voting Behaviour
Source: PLoS One. 2016 Jul 8;11(7):e0159017. doi: 10.1371/journal.pone.0159017 (PMC4938543; doi:10.1371/journal.pone.0159017)
Supplement: S7 Table — The first block shows all polls coded according to the days on which data was collected. Subsequent blocks show polls that include versus exclude each weekday in turn. Rows refer to separate polls. Columns contain response numbers, start and end dates, start and end days, presence (1) or absence (0) of each weekday, and poll results. (PDF) [file pone.0159017.s007.pdf]

S7 Table. Leave One Day Out (LODO) analysis of voting intentions ahead of the United Kingdom European Union membership referendum. The first block shows all polls coded according to the days on which data was collected. Subsequent blocks show polls that *include* versus *exclude* each weekday in turn. Rows refer to separate polls. Columns contain response numbers, start and end dates, start and end days, presence (1) or absence (0) of each weekday, and poll results.

ALL POLLS

Fieldwork End Date / Pollster	StartDate	EndDate	Participants	StartDay	EndDay	Length	Startday	EndDay	Monday?	Tuesday?	Wednesday?	Thursday?	Friday?	Remain	Leave	Don't know	All-DK	Remain-DK	Leave-DK
4 September 2015 / Survation	03/09/15	04/09/15	1004	4	5	2	Thursday	Friday	0	0	0	1	1	40.00%	43.00%	17.00%	83.00%	48.19%	51.81%
6 September 2015 / ORB	04/09/15	06/09/15	2044	5	7	3	Friday	Sunday	0	0	0	0	1	55.00%	45.00%	0.00%	100.00%	55.00%	45.00%
13 September 2015 / ICM	11/09/15	13/09/15	2006	5	7	3	Friday	Sunday	0	0	0	0	1	43.00%	40.00%	17.00%	83.00%	51.81%	48.19%
17 September 2015 / YouGov	10/09/15	17/09/15	11171	4	4	8	Thursday	Thursday	1	1	1	1	1	38.00%	40.00%	22.00%	78.00%	48.72%	51.28%
20 September 2015 / ICM	18/09/15	20/09/15	2005	5	7	3	Friday	Sunday	0	0	0	0	1	44.00%	37.00%	19.00%	81.00%	54.32%	45.68%
22 September 2015 / Survation	21/09/15	22/09/15	1008	1	2	2	Monday	Tuesday	1	1	0	0	0	43.00%	40.00%	17.00%	83.00%	51.81%	48.19%
27 September 2015 / ICM	25/09/15	27/09/15	2005	5	7	3	Friday	Sunday	0	0	0	0	1	45.00%	38.00%	17.00%	83.00%	54.22%	45.78%
28 September 2015 / ComRes	25/09/15	28/09/15	1009	5	1	4	Friday	Monday	1	0	0	0	1	55.00%	36.00%	8.00%	91.00%	60.44%	39.56%
1 October 2015 / YouGov	24/09/15	01/10/15	533	4	4	8	Thursday	Thursday	1	1	1	1	1	38.00%	42.00%	20.00%	80.00%	47.50%	52.50%
4 October 2015 / ICM	02/10/15	04/10/15	2034	5	7	3	Friday	Sunday	0	0	0	0	1	42.00%	38.00%	20.00%	80.00%	52.50%	47.50%
7 October 2015 / ICM	07/10/15	07/10/15	1947	3	3	1	Wednesday	Wednesday	1	1	1	1	1	44.00%	39.00%	17.00%	83.00%	53.01%	46.99%
11 October 2015 / ICM	09/10/15	11/10/15	2036	5	7	3	Friday	Sunday	0	0	0	0	1	45.00%	36.00%	18.00%	81.00%	55.56%	44.44%
12 October 2015 / YouGov	01/10/15	12/10/15	1030	4	1	12	Thursday	Monday	1	1	1	1	1	43.00%	41.00%	16.00%	84.00%	51.19%	48.81%
18 October 2015 / ICM	16/10/15	18/10/15	2023	5	7	3	Friday	Sunday	0	0	0	0	1	44.00%	38.00%	18.00%	82.00%	53.66%	46.34%
19 October 2015 / Greenberg Quinlan Rosner Research	14/10/15	19/10/15	2372	3	1	6	Wednesday	Monday	1	0	1	1	1	45.00%	42.00%	12.00%	87.00%	51.72%	48.28%
19 October 2015 / Ipsos MORI	17/10/15	19/10/15	1021	6	1	3	Saturday	Monday	1	0	0	0	0	52.00%	36.00%	12.00%	88.00%	59.09%	40.91%
20 October 2015 / YouGov	19/10/15	20/10/15	1690	1	2	2	Monday	Tuesday	1	1	0	0	0	42.00%	40.00%	17.00%	82.00%	51.22%	48.78%
23 October 2015 / YouGov	22/10/15	23/10/15	1625	4	5	2	Thursday	Friday	0	0	0	1	1	42.00%	39.00%	19.00%	81.00%	51.85%	48.15%
25 October 2015 / ORB	23/10/15	25/10/15	2015	5	7	3	Friday	Sunday	0	0	0	0	1	53.00%	47.00%	0.00%	100.00%	53.00%	47.00%
25 October 2015 / ICM	23/10/15	25/10/15	2049	5	7	3	Friday	Sunday	0	0	0	0	1	45.00%	38.00%	17.00%	83.00%	54.22%	45.78%
27 October 2015 / BMG Research	22/10/15	27/10/15	1467	4	2	6	Thursday	Tuesday	1	1	0	1	1	46.00%	43.00%	12.00%	89.00%	51.69%	48.31%
29 October 2015 / YouGov	28/10/15	29/10/15	1664	3	4	2	Wednesday	Thursday	0	0	1	1	0	39.00%	41.00%	19.00%	80.00%	48.75%	51.25%
1 November 2015 / ICM	30/10/15	01/11/15	2060	5	7	3	Friday	Sunday	0	0	0	0	1	44.00%	38.00%	18.00%	82.00%	53.66%	46.34%
8 November 2015 / ICM	06/11/15	08/11/15	2024	5	7	3	Friday	Sunday	0	0	0	0	1	46.00%	38.00%	16.00%	84.00%	54.76%	45.24%
11 November 2015 / Survation	09/11/15	11/11/15	2007	1	3	3	Monday	Wednesday	1	1	1	0	0	39.00%	44.00%	19.00%	83.00%	46.99%	53.01%
15 November 2015 / ICM	13/11/15	15/11/15	2000	5	7	3	Friday	Sunday	0	0	0	0	1	43.00%	38.00%	19.00%	81.00%	53.09%	46.91%
17 November 2015 / BMG Research	11/11/15	17/11/15	1528	3	2	7	Wednesday	Tuesday	1	1	1	1	1	43.00%	43.00%	15.00%	86.00%	50.00%	50.00%
17 November 2015 / Survation	16/11/15	17/11/15	1546	1	2	2	Monday	Tuesday	1	1	0	0	0	43.00%	40.00%	18.00%	83.00%	51.81%	48.19%
19 November 2015 / ORB	18/11/15	19/11/15	2067	3	4	2	Wednesday	Thursday	0	0	1	1	0	48.00%	52.00%	0.00%	100.00%	48.00%	52.00%
22 November 2015 / ICM	20/11/15	22/11/15	2002	5	7	3	Friday	Sunday	0	0	0	0	1	45.00%	38.00%	17.00%	83.00%	54.22%	45.78%
24 November 2015 / YouGov	20/11/15	24/11/15	4317	5	2	5	Friday	Tuesday	1	1	0	1	1	41.00%	41.00%	18.00%	82.00%	50.00%	50.00%
29 November 2015 / ICM	27/11/15	29/11/15	2053	5	7	3	Friday	Sunday	0	0	0	0	1	43.00%	39.00%	17.00%	82.00%	52.44%	47.56%
3 December 2015 / Survation	30/11/15	03/12/15	10015	1	4	4	Monday	Thursday	1	1	1	1	0	40.00%	42.00%	18.00%	82.00%	48.78%	51.22%
6 December 2015 / ICM	04/12/15	06/12/15	2022	5	7	3	Friday	Sunday	0	0	0	0	1	43.00%	39.00%	17.00%	82.00%	52.44%	47.56%
13 December 2015 / ComRes	11/12/15	13/12/15	1001	5	7	3	Friday	Sunday	0	0	0	0	1	56.00%	35.00%	8.00%	91.00%	61.54%	38.46%
13 December 2015 / ICM	11/12/15	13/12/15	2053	5	7	3	Friday	Sunday	0	0	0	0	1	42.00%	41.00%	17.00%	83.00%	50.60%	49.40%
14 December 2015 / Ipsos MORI	12/12/15	14/12/15	529	6	1	3	Saturday	Monday	1	0	0	0	0	58.00%	32.00%	10.00%	90.00%	64.44%	35.56%
17 December 2015 / ORB	16/12/15	17/12/15	2038	3	4	2	Wednesday	Thursday	0	0	1	1	0	52.00%	48.00%	0.00%	100.00%	52.00%	48.00%
18 December 2015 / YouGov	17/12/15	18/12/15	1598	4	5	2	Thursday	Friday	0	0	0	1	1	41.00%	42.00%	17.00%	83.00%	49.40%	50.60%
20 December 2015 / ICM	18/12/15	20/12/15	2017	5	7	3	Friday	Sunday	0	0	0	0	1	45.00%	38.00%	17.00%	83.00%	54.22%	45.78%
10 January 2016 / ICM	08/01/16	10/01/16	2055	5	7	3	Friday	Sunday	0	0	0	0	1	44.00%	38.00%	18.00%	82.00%	53.66%	46.34%
14 January 2016 / Panelbase	08/01/16	14/01/16	2087	5	4	7	Friday	Thursday	1	1	1	1	1	42.00%	45.00%	12.00%	87.00%	48.28%	51.72%
16 January 2016 / Survation	15/01/16	16/01/16	1017	5	6	2	Friday	Saturday	0	0	0	0	1	38.00%	42.00%	21.00%	80.00%	47.50%	52.50%
17 January 2016 / ICM	15/01/16	17/01/16	2023	5	7	3	Friday	Sunday	0	0	0	0	1	42.00%	40.00%	17.00%	82.00%	51.22%	48.78%
21 January 2016 / ORB	20/01/16	21/01/16	2015	3	4	2	Wednesday	Thursday	0	0	1	1	0	52.00%	48.00%	0.00%	100.00%	52.00%	48.00%
24 January 2016 / Opinium	20/01/16	24/01/16	1033	3	7	5	Wednesday	Sunday	0	0	1	1	1	40.00%	41.00%	19.00%	81.00%	49.38%	50.62%
24 January 2016 / ComRes	22/01/16	24/01/16	1006	5	7	3	Friday	Sunday	0	0	0	0	1	54.00%	36.00%	10.00%	90.00%	60.00%	40.00%
24 January 2016 / ICM	22/01/16	24/01/16	2010	5	7	3	Friday	Sunday	0	0	0	0	1	41.00%	41.00%	18.00%	82.00%	50.00%	50.00%
25 January 2016 / Ipsos MORI	23/01/16	25/01/16	513	6	1	3	Saturday	Monday	1	0	0	0	0	55.00%	36.00%	9.00%	91.00%	60.44%	39.56%
25 January 2016 / BMG Research	21/01/16	25/01/16	1511	4	1	5	Thursday	Monday	1	0	0	1	1	44.00%	42.00%	14.00%	86.00%	51.16%	48.84%
28 January 2016 / YouGov	27/01/16	28/01/16	1735	3	4	2	Wednesday	Thursday	0	0	1	1	0	38.00%	42.00%	20.00%	80.00%	47.50%	52.50%
31 January 2016 / ICM	29/01/16	31/01/16	2002	5	7	3	Friday	Sunday	0	0	0	0	1	42.00%	39.00%	19.00%	81.00%	51.85%	48.15%
4 February 2016 / YouGov	03/02/16	04/02/16	1675	3	4	2	Wednesday	Thursday	0	0	1	1	0	36.00%	45.00%	19.00%	81.00%	44.44%	55.56%
7 February 2016 / ICM	05/02/16	07/02/16	2018	5	7	3	Friday	Sunday	0	0	0	0	1	41.00%	42.00%	17.00%	83.00%	49.40%	50.60%
14 February 2016 / ICM	12/02/16	14/02/16	2001	5	7	3	Friday	Sunday	0	0	0	0	1	43.00%	39.00%	18.00%	82.00%	52.44%	47.56%
14 February 2016 / ComRes	11/02/16	14/02/16	1105	4	7	4	Thursday	Sunday	0	0	0	1	1	49.00%	41.00%	10.00%	90.00%	54.44%	45.56%
15 February 2016 / TNS	11/02/16	15/02/16	1120	4	1	5	Thursday	Monday	1	0	0	1	1	36.00%	39.00%	25.00%	75.00%	48.00%	52.00%
16 February 2016 / Ipsos MORI	13/02/16	16/02/16	1001	6	2	4	Saturday	Tuesday	1	1	0	0	0	54.00%	36.00%	10.00%	90.00%	60.00%	40.00%
20 February 2016 / Survation	20/02/16	20/02/16	1002	6	6	1	Saturday	Saturday	0	0	0	0	0	48.00%	33.00%	19.00%	81.00%	59.26%	40.74%
22 February 2016 / ComRes	19/02/16	22/02/16	1000	5	1	4	Friday	Monday	1	0	0	0	1	52.00%	39.00%	10.00%	91.00%	57.14%	42.86%
22 February 2016 / ICM	19/02/16	22/02/16	2021	5	1	4	Friday	Monday	1	0	0	0	1	42.00%	40.00%	17.00%	82.00%	51.22%	48.78%
23 February 2016 / BMG Research	17/02/16	23/02/16	1517	3	2	7	Wednesday	Tuesday	1	1	1	1	1	44.00%	41.00%	15.00%	85.00%	51.76%	48.24%
23 February 2016 / YouGov	22/02/16	23/02/16	3482	1	2	2	Monday	Tuesday	1	1	0	0	0	37.00%	38.00%	25.00%	75.00%	49.33%	50.67%
25 February 2016 / ORB	24/02/16	25/02/16	2014	3	4	2	Wednesday	Thursday	0	0	1	1	1	48.00%	52.00%	0.00%	100.00%	48.00%	52.00%
25 February 2016 / YouGov	25/02/16	25/02/16	1731	4	4	1	Thursday	Thursday	0	0	0	1	0	41.00%	38.00%	21.00%	79.00%	51.90%	48.10%
29 February 2016 / ICM	26/02/16	29/02/16	2003	5	1	4	Friday	Monday	1	0	0	0	1	41.00%	41.00%	18.00%	82.00%	50.00%	50.00%
1 March 2016 / YouGov	29/02/16	01/03/16	2233	1	2	2	Monday	Tuesday	1	1	0	0	0	39.00%	37.00%	24.00%	76.00%	51.32%	48.68%
2 March 2016 / YouGov	01/03/16	02/03/16	1705	2	3	2	Tuesday	Wednesday	0	1	1	0	0	40.00%	35.00%	24.00%	75.00%	53.33%	46.67%
3 March 2016 / YouGov	02/03/16	03/03/16	1695	3	4	2	Wednesday	Thursday	0	1	1	1	0	40.00%	37.00%	23.00%	77.00%	51.95%	48.05%
6 March 2016 / ICM	04/03/16	06/03/16	2051	5	7	3	Friday	Sunday	0	0	0	0	1	40.00%	41.00%	19.00%	81.00%	49.38%	50.62%
13 March 2016 / ICM	11/03/16	13/03/16	2031	5	7	3	Friday	Sunday	0	0	0	0	1	43.00%	41.00%	16.00%	84.00%	51.19%	48.81%
14 March 2016 / TNS	11/03/16	14/03/16	1216	5	1	4	Friday	Monday	1	0	0	0	1	36.00%	36.00%	28.00%	72.00%	50.00%	50.00%
14 March 2016 / ORB	11/03/16	14/03/16	823	5	1	4	Friday	Monday	1	0	0	0	1	47.00%	49.00%	4.00%	96.00%	48.96%	51.04%
19 March 2016 / Survation	17/03/16	19/03/16	1006	4	6	3	Thursday	Saturday	0	0	0	1	1	46.00%	35.00%	19.00%	81.00%	56.79%	43.21%
20 March 2016 / ComRes	18/03/16	20/03/16	1002	5	7	3	Friday	Sunday	0	0	0	0	1	48.00%	41.00%	11.00%	89.00%	53.93%	46.07%
20 March 2016 / ICM	18/03/16	20/03/16	2000	5	7	3	Friday	Sunday	0	0	0	0	1	41.00%	43.00%	17.00%	84.00%	48.81%	51.19%
24 March 2016 / ICM	22/03/16	24/03/16	1970	2	4	3	Tuesday	Thursday	0	1	1	1	0	45.00%	43.00%	12.00%	88.00%	51.14%	48.86%
Total			149064										Mean	44.21%	40.25%	15.49%	84.45%	52.27%	47.73%

MONDAYS INCLUDED

Fieldwork End Date / Pollster	StartDate	EndDate	Participants	StartDay	EndDay	Length	Startday	EndDay	Monday?	Tuesday?	Wednesday?	Thursday?	Friday?	Remain	Leave	Don't know	All-DK	Remain-DK	Leave-DK
17 September 2015 / YouGov	10/09/15	17/09/15	11171	4	4	8	Thursday	Thursday	1	1	1	1	1	38.00%	40.00%	22.00%	78.00%	48.72%	51.28%
22 September 2015 / Survation	21/09/15	22/09/15	1008	1	2	2	Monday	Tuesday	1	1	0	0	0	43.00%	40.00%	17.00%	83.00%	51.81%	48.19%
28 September 2015 / ComRes	25/09/15	28/09/15	1009	5	1	4	Friday	Monday	1	0	0	0	1	55.00%	36.00%	8.00%	91.00%	60.44%	39.56%
1 October 2015 / YouGov	24/09/15	01/10/15	533	4	4	8	Thursday	Thursday	1	1	1	1	1	38.00%	42.00%	20.00%	80.00%	47.50%	52.50%
7 October 2015 / ICM	07/10/15	07/10/15	1947	3	3	1	Wednesday	Wednesday	1	1	1	1	1	44.00%	39.00%	17.00%	83.00%	53.01%	46.99%
12 October 2015 / YouGov	01/10/15	12/10/15	1030	4	1	12	Thursday	Monday	1	1	1	1	1	43.00%	41.00%	16.00%	84.00%	51.19%	48.81%
19 October 2015 / Greenberg Quinlan Rosner Research	14/10/15	19/10/15	2372	3	1	6	Wednesday	Monday	1	0	1	1	1	45.00%	42.00%	12.00%	87.00%	51.72%	48.28%
19 October 2015 / Ipsos MORI	17/10/15	19/10/15	1021	6	1	3	Saturday	Monday	1	0	0	0	0	52.00%	36.00%	12.00%	88.00%	59.09%	40.91%
20 October 2015 / YouGov	19/10/15	20/10/15	1690	1	2	2	Monday	Tuesday	1	1	0	0	0	42.00%	40.00%	17.00%	82.00%	51.22%	48.78%
27 October 2015 / BMG Research	22/10/15	27/10/15	1467	4	2	6	Thursday	Tuesday	1	1	0	1	1	46.00%	43.00%	12.00%	89.00%	51.69%	48.31%
11 November 2015 / Survation	09/11/15	11/11/15	2007	1	3	3	Monday	Wednesday	1	1	1	0	0	39.00%	44.00%	19.00%	83.00%	46.99%	53.01%
17 November 2015 / BMG Research	11/11/15	17/11/15	1528	3	2	7	Wednesday	Tuesday	1	1	1	1	1	43.00%	43.00%	15.00%	86.00%	50.00%	50.00%
17 November 2015 / Survation	16/11/15	17/11/15	1546	1	2	2	Monday	Tuesday	1	1	0	0	0	43.00%	40.00%	18.00%	83.00%	51.81%	48.19%
24 November 2015 / YouGov	20/11/15	24/11/15	4317	5	2	5	Friday	Tuesday	1	1	0	1	1	41.00%	41.00%	18.00%	82.00%	50.00%	50.00%
3 December 2015 / Survation	30/11/15	03/12/15	10015	1	4	4	Monday	Thursday	1	1	1	1	0	40.00%	42.00%	18.00%	82.00%	48.78%	51.22%
14 December 2015 / Ipsos MORI	12/12/15	14/12/15	529	6	1	3	Saturday	Monday	1	0	0	0	0	58.00%	32.00%	10.00%	90.00%	64.44%	35.56%
14 January 2016 / Panelbase	08/01/16	14/01/16	2087	5	4	7	Friday	Thursday	1	1	1	1	1	42.00%	45.00%	12.00%	87.00%	48.28%	51.72%
25 January 2016 / Ipsos MORI	23/01/16	25/01/16	513	6	1	3	Saturday	Monday	1	0	0	0	0	55.00%	36.00%	9.00%	91.00%	60.44%	39.56%
25 January 2016 / BMG Research	21/01/16	25/01/16	1511	4	1	5	Thursday	Monday	1	0	0	1	1	44.00%	42.00%	14.00%	86.00%	51.16%	48.84%
15 February 2016 / TNS	11/02/16	15/02/16	1120	4	1	5	Thursday	Monday	1	0	0	1	1	36.00%	39.00%	25.00%	75.00%	48.00%	52.00%
16 February 2016 / Ipsos MORI	13/02/16	16/02/16	1001	6	2	4	Saturday	Tuesday	1	1	0	0	0	54.00%	36.00%	10.00%	90.00%	60.00%	40.00%
22 February 2016 / ComRes	19/02/16	22/02/16	1000	5	1	4	Friday	Monday	1	0	0	0	1	52.00%	39.00%	10.00%	91.00%	57.14%	42.86%
22 February 2016 / ICM	19/02/16	22/02/16	2021	5	1	4	Friday	Monday	1	0	0	0	1	42.00%	40.00%	17.00%	82.00%	51.22%	48.78%
23 February 2016 / BMG Research	17/02/16	23/02/16	1517	3	2	7	Wednesday	Tuesday	1	1	1	1	1	44.00%	41.00%	15.00%	85.00%	51.76%	48.24%
23 February 2016 / YouGov	22/02/16	23/02/16	3482	1	2	2	Monday	Tuesday	1	1	0	0	0	37.00%	38.00%	25.00%	75.00%	49.33%	50.67%
29 February 2016 / ICM	26/02/16	29/02/16	2003	5	1	4	Friday	Monday	1	0	0	0	1	41.00%	41.00%	18.00%	82.00%	50.00%	50.00%
1 March 2016 / YouGov	29/02/16	01/03/16	2233	1	2	2	Monday	Tuesday	1	1	0	0	0	39.00%	37.00%	24.00%	76.00%	51.32%	48.68%
14 March 2016 / TNS	11/03/16	14/03/16	1216	5	1	4	Friday	Monday	1	0	0	0	1	36.00%	36.00%	28.00%	72.00%	50.00%	50.00%
14 March 2016 / ORB	11/03/16	14/03/16	823	5	1	4	Friday	Monday	1	0	0	0	1	47.00%	49.00%	4.00%	96.00%	48.96%	51.04%
Total			63717										Mean	44.10%	40.00%	15.93%	84.10%	52.28%	47.72%

MONDAYS EXCLUDED

Fieldwork End Date / Pollster	StartDate	EndDate	Participants	StartDay	EndDay	Length	Startday	EndDay	Monday?	Tuesday?	Wednesday?	Thursday?	Friday?	Remain	Leave	Don't know	All-DK	Remain-DK	Leave-DK
4 September 2015 / Survation	03/09/15	04/09/15	1004	4	5	2	Thursday	Friday	0	0	0	1	1	40.00%	43.00%	17.00%	83.00%	48.19%	51.81%
6 September 2015 / ORB	04/09/15	06/09/15	2044	5	7	3	Friday	Sunday	0	0	0	0	1	55.00%	45.00%	0.00%	100.00%	55.00%	45.00%
13 September 2015 / ICM	11/09/15	13/09/15	2006	5	7	3	Friday	Sunday	0	0	0	0	1	43.00%	40.00%	17.00%	83.00%	51.81%	48.19%
20 September 2015 / ICM	18/09/15	20/09/15	2005	5	7	3	Friday	Sunday	0	0	0	0	1	44.00%	37.00%	19.00%	81.00%	54.32%	45.68%
27 September 2015 / ICM	25/09/15	27/09/15	2005	5	7	3	Friday	Sunday	0	0	0	0	1	45.00%	38.00%	17.00%	83.00%	54.22%	45.78%
4 October 2015 / ICM	02/10/15	04/10/15	2034	5	7	3	Friday	Sunday	0	0	0	0	1	42.00%	38.00%	20.00%	80.00%	52.50%	47.50%
11 October 2015 / ICM	09/10/15	11/10/15	2036	5	7	3	Friday	Sunday	0	0	0	0	1	45.00%	36.00%	18.00%	81.00%	55.56%	44.44%
18 October 2015 / ICM	16/10/15	18/10/15	2023	5	7	3	Friday	Sunday	0	0	0	0	1	44.00%	38.00%	18.00%	82.00%	53.66%	46.34%
23 October 2015 / YouGov	22/10/15	23/10/15	1625	4	5	2	Thursday	Friday	0	0	0	1	1	42.00%	39.00%	19.00%	81.00%	51.85%	48.15%
25 October 2015 / ORB	23/10/15	25/10/15	2015	5	7	3	Friday	Sunday	0	0	0	0	1	53.00%	47.00%	0.00%	100.00%	53.00%	47.00%
25 October 2015 / ICM	23/10/15	25/10/15	2049	5	7	3	Friday	Sunday	0	0	0	0	1	45.00%	38.00%	17.00%	83.00%	54.22%	45.78%
29 October 2015 / YouGov	28/10/15	29/10/15	1664	3	4	2	Wednesday	Thursday	0	0	1	1	0	39.00%	41.00%	19.00%	80.00%	48.75%	51.25%
1 November 2015 / ICM	30/10/15	01/11/15	2060	5	7	3	Friday	Sunday	0	0	0	0	1	44.00%	38.00%	18.00%	82.00%	53.66%	46.34%
8 November 2015 / ICM	06/11/15	08/11/15	2024	5	7	3	Friday	Sunday	0	0	0	0	1	46.00%	38.00%	16.00%	84.00%	54.76%	45.24%
15 November 2015 / ICM	13/11/15	15/11/15	2000	5	7	3	Friday	Sunday	0	0	0	0	1	43.00%	38.00%	19.00%	81.00%	53.09%	46.91%
19 November 2015 / ORB	18/11/15	19/11/15	2067	3	4	2	Wednesday	Thursday	0	0	1	1	0	48.00%	52.00%	0.00%	100.00%	48.00%	52.00%
22 November 2015 / ICM	20/11/15	22/11/15	2002	5	7	3	Friday	Sunday	0	0	0	0	1	45.00%	38.00%	17.00%	83.00%	54.22%	45.78%
29 November 2015 / ICM	27/11/15	29/11/15	2053	5	7	3	Friday	Sunday	0	0	0	0	1	43.00%	39.00%	17.00%	82.00%	52.44%	47.56%
6 December 2015 / ICM	04/12/15	06/12/15	2022	5	7	3	Friday	Sunday	0	0	0	0	1	43.00%	39.00%	17.00%	82.00%	52.44%	47.56%
13 December 2015 / ComRes	11/12/15	13/12/15	1001	5	7	3	Friday	Sunday	0	0	0	0	1	56.00%	35.00%	8.00%	91.00%	61.54%	38.46%
13 December 2015 / ICM	11/12/15	13/12/15	2053	5	7	3	Friday	Sunday	0	0	0	0	1	42.00%	41.00%	17.00%	83.00%	50.60%	49.40%
17 December 2015 / ORB	16/12/15	17/12/15	2038	3	4	2	Wednesday	Thursday	0	0	1	1	0	52.00%	48.00%	0.00%	100.00%	52.00%	48.00%
18 December 2015 / YouGov	17/12/15	18/12/15	1598	4	5	2	Thursday	Friday	0	0	0	1	1	41.00%	42.00%	17.00%	83.00%	49.40%	50.60%
20 December 2015 / ICM	18/12/15	20/12/15	2017	5	7	3	Friday	Sunday	0	0	0	0	1	45.00%	38.00%	17.00%	83.00%	54.22%	45.78%
10 January 2016 / ICM	08/01/16	10/01/16	2055	5	7	3	Friday	Sunday	0	0	0	0	1	44.00%	38.00%	18.00%	82.00%	53.66%	46.34%
16 January 2016 / Survation	15/01/16	16/01/16	1017	5	6	2	Friday	Saturday	0	0	0	0	1	38.00%	42.00%	21.00%	80.00%	47.50%	52.50%
17 January 2016 / ICM	15/01/16	17/01/16	2023	5	7	3	Friday	Sunday	0	0	0	0	1	42.00%	40.00%	17.00%	82.00%	51.22%	48.78%
21 January 2016 / ORB	20/01/16	21/01/16	2015	3	4	2	Wednesday	Thursday	0	0	1	1	0	52.00%	48.00%	0.00%	100.00%	52.00%	48.00%
24 January 2016 / Opinium	20/01/16	24/01/16	1033	3	7	5	Wednesday	Sunday	0	0	1	1	1	40.00%	41.00%	19.00%	81.00%	49.38%	50.62%
24 January 2016 / ComRes	22/01/16	24/01/16	1006	5	7	3	Friday	Sunday	0	0	0	0	1	54.00%	36.00%	10.00%	90.00%	60.00%	40.00%
24 January 2016 / ICM	22/01/16	24/01/16	2010	5	7	3	Friday	Sunday	0	0	0	0	1	41.00%	41.00%	18.00%	82.00%	50.00%	50.00%
28 January 2016 / YouGov	27/01/16	28/01/16	1735	3	4	2	Wednesday	Thursday	0	0	1	1	0	38.00%	42.00%	20.00%	80.00%	47.50%	52.50%
31 January 2016 / ICM	29/01/16	31/01/16	2002	5	7	3	Friday	Sunday	0	0	0	0	1	42.00%	39.00%	19.00%	81.00%	51.85%	48.15%
4 February 2016 / YouGov	03/02/16	04/02/16	1675	3	4	2	Wednesday	Thursday	0	0	1	1	0	36.00%	45.00%	19.00%	81.00%	44.44%	55.56%
7 February 2016 / ICM	05/02/16	07/02/16	2018	5	7	3	Friday	Sunday	0	0	0	0	1	41.00%	42.00%	17.00%	83.00%	49.40%	50.60%
14 February 2016 / ICM	12/02/16	14/02/16	2001	5	7	3	Friday	Sunday	0	0	0	0	1	43.00%	39.00%	18.00%	82.00%	52.44%	47.56%
14 February 2016 / ComRes	11/02/16	14/02/16	1105	4	7	4	Thursday	Sunday	0	0	0	1	1	49.00%	41.00%	10.00%	90.00%	54.44%	45.56%
20 February 2016 / Survation	20/02/16	20/02/16	1002	6	6	1	Saturday	Saturday	0	0	0	0	0	48.00%	33.00%	19.00%	81.00%	59.26%	40.74%
25 February 2016 / ORB	24/02/16	25/02/16	2014	3	4	2	Wednesday	Thursday	0	0	1	1	1	48.00%	52.00%	0.00%	100.00%	48.00%	52.00%
25 February 2016 / YouGov	25/02/16	25/02/16	1731	4	4	1	Thursday	Thursday	0	0	0	1	0	41.00%	38.00%	21.00%	79.00%	51.90%	48.10%
2 March 2016 / YouGov	01/03/16	02/03/16	1705	2	3	2	Tuesday	Wednesday	0	1	1	0	0	40.00%	35.00%	24.00%	75.00%	53.33%	46.67%
3 March 2016 / YouGov	02/03/16	03/03/16	1695	3	4	2	Wednesday	Thursday	0	1	1	1	0	40.00%	37.00%	23.00%	77.00%	51.95%	48.05%
6 March 2016 / ICM	04/03/16	06/03/16	2051	5	7	3	Friday	Sunday	0	0	0	0	1	40.00%	41.00%	19.00%	81.00%	49.38%	50.62%
13 March 2016 / ICM	11/03/16	13/03/16	2031	5	7	3	Friday	Sunday	0	0	0	0	1	43.00%	41.00%	16.00%	84.00%	51.19%	48.81%
19 March 2016 / Survation	17/03/16	19/03/16	1006	4	6	3	Thursday	Saturday	0	0	0	1	1	46.00%	35.00%	19.00%	81.00%	56.79%	43.21%
20 March 2016 / ComRes	18/03/16	20/03/16	1002	5	7	3	Friday	Sunday	0	0	0	0	1	48.00%	41.00%	11.00%	89.00%	53.93%	46.07%
20 March 2016 / ICM	18/03/16	20/03/16	2000	5	7	3	Friday	Sunday	0	0	0	0	1	41.00%	43.00%	17.00%	84.00%	48.81%	51.19%
24 March 2016 / ICM	22/03/16	24/03/16	1970	2	4	3	Tuesday	Thursday	0	1	1	1	0	45.00%	43.00%	12.00%	88.00%	51.14%	48.86%
Total			85347										Mean	44.27%	40.40%	15.23%	84.67%	52.27%	47.73%

TUESDAYS INCLUDED

Fieldwork End Date / Pollster	StartDate	EndDate	Participants	StartDay	EndDay	Length	Startday	EndDay	Monday?	Tuesday?	Wednesday?	Thursday?	Friday?	Remain a member of	Leave the EU	Don't know	All-DK	Remain-DK	Leave-DK
17 September 2015 / YouGov	10/09/15	17/09/15	11171	4	4	8	Thursday	Thursday	1	1	1	1	1	38.00%	40.00%	22.00%	78.00%	48.72%	51.28%
22 September 2015 / Survation	21/09/15	22/09/15	1008	1	2	2	Monday	Tuesday	1	1	0	0	0	43.00%	40.00%	17.00%	83.00%	51.81%	48.19%
1 October 2015 / YouGov	24/09/15	01/10/15	533	4	4	8	Thursday	Thursday	1	1	1	1	1	38.00%	42.00%	20.00%	80.00%	47.50%	52.50%
7 October 2015 / ICM	07/10/15	07/10/15	1947	3	3	1	Wednesday	Wednesday	1	1	1	1	1	44.00%	39.00%	17.00%	83.00%	53.01%	46.99%
12 October 2015 / YouGov	01/10/15	12/10/15	1030	4	1	12	Thursday	Monday	1	1	1	1	1	43.00%	41.00%	16.00%	84.00%	51.19%	48.81%
20 October 2015 / YouGov	19/10/15	20/10/15	1690	1	2	2	Monday	Tuesday	1	1	0	0	0	42.00%	40.00%	17.00%	82.00%	51.22%	48.78%
27 October 2015 / BMG Research	22/10/15	27/10/15	1467	4	2	6	Thursday	Tuesday	1	1	0	1	1	46.00%	43.00%	12.00%	89.00%	51.69%	48.31%
11 November 2015 / Survation	09/11/15	11/11/15	2007	1	3	3	Monday	Wednesday	1	1	1	0	0	39.00%	44.00%	19.00%	83.00%	46.99%	53.01%
17 November 2015 / BMG Research	11/11/15	17/11/15	1528	3	2	7	Wednesday	Tuesday	1	1	1	1	1	43.00%	43.00%	15.00%	86.00%	50.00%	50.00%
17 November 2015 / Survation	16/11/15	17/11/15	1546	1	2	2	Monday	Tuesday	1	1	0	0	0	43.00%	40.00%	18.00%	83.00%	51.81%	48.19%
24 November 2015 / YouGov	20/11/15	24/11/15	4317	5	2	5	Friday	Tuesday	1	1	0	1	1	41.00%	41.00%	18.00%	82.00%	50.00%	50.00%
3 December 2015 / Survation	30/11/15	03/12/15	10015	1	4	4	Monday	Thursday	1	1	1	1	0	40.00%	42.00%	18.00%	82.00%	48.78%	51.22%
14 January 2016 / Panelbase	08/01/16	14/01/16	2087	5	4	7	Friday	Thursday	1	1	1	1	1	42.00%	45.00%	12.00%	87.00%	48.28%	51.72%
16 February 2016 / Ipsos MORI	13/02/16	16/02/16	1001	6	2	4	Saturday	Tuesday	1	1	0	0	0	54.00%	36.00%	10.00%	90.00%	60.00%	40.00%
23 February 2016 / BMG Research	17/02/16	23/02/16	1517	3	2	7	Wednesday	Tuesday	1	1	1	1	1	44.00%	41.00%	15.00%	85.00%	51.76%	48.24%
23 February 2016 / YouGov	22/02/16	23/02/16	3482	1	2	2	Monday	Tuesday	1	1	0	0	0	37.00%	38.00%	25.00%	75.00%	49.33%	50.67%
1 March 2016 / YouGov	29/02/16	01/03/16	2233	1	2	2	Monday	Tuesday	1	1	0	0	0	39.00%	37.00%	24.00%	76.00%	51.32%	48.68%
2 March 2016 / YouGov	01/03/16	02/03/16	1705	2	3	2	Tuesday	Wednesday	0	1	1	0	0	40.00%	35.00%	24.00%	75.00%	53.33%	46.67%
3 March 2016 / YouGov	02/03/16	03/03/16	1695	3	4	2	Wednesday	Thursday	0	1	1	1	0	40.00%	37.00%	23.00%	77.00%	51.95%	48.05%
24 March 2016 / ICM	22/03/16	24/03/16	1970	2	4	3	Tuesday	Thursday	0	1	1	1	0	45.00%	43.00%	12.00%	88.00%	51.14%	48.86%
Total			53949										Mean	42.05%	40.35%	17.70%	82.40%	50.99%	49.01%

TUESDAYS EXCLUDED

Fieldwork End Date / Pollster	StartDate	EndDate	Participants	StartDay	EndDay	Length	Startday	EndDay	Monday?	Tuesday?	Wednesday?	Thursday?	Friday?	Remain	Leave	Don't know	All-DK	Remain-DK	Leave-DK
4 September 2015 / Survation	03/09/15	04/09/15	1004	4	5	2	Thursday	Friday	0	0	0	1	1	40.00%	43.00%	17.00%	83.00%	48.19%	51.81%
6 September 2015 / ORB	04/09/15	06/09/15	2044	5	7	3	Friday	Sunday	0	0	0	0	1	55.00%	45.00%	0.00%	100.00%	55.00%	45.00%
13 September 2015 / ICM	11/09/15	13/09/15	2006	5	7	3	Friday	Sunday	0	0	0	0	1	43.00%	40.00%	17.00%	83.00%	51.81%	48.19%
20 September 2015 / ICM	18/09/15	20/09/15	2005	5	7	3	Friday	Sunday	0	0	0	0	1	44.00%	37.00%	19.00%	81.00%	54.32%	45.68%
27 September 2015 / ICM	25/09/15	27/09/15	2005	5	7	3	Friday	Sunday	0	0	0	0	1	45.00%	38.00%	17.00%	83.00%	54.22%	45.78%
28 September 2015 / ComRes	25/09/15	28/09/15	1009	5	1	4	Friday	Monday	1	0	0	0	1	55.00%	36.00%	8.00%	91.00%	60.44%	39.56%
4 October 2015 / ICM	02/10/15	04/10/15	2034	5	7	3	Friday	Sunday	0	0	0	0	1	42.00%	38.00%	20.00%	80.00%	52.50%	47.50%
11 October 2015 / ICM	09/10/15	11/10/15	2036	5	7	3	Friday	Sunday	0	0	0	0	1	45.00%	36.00%	18.00%	81.00%	55.56%	44.44%
18 October 2015 / ICM	16/10/15	18/10/15	2023	5	7	3	Friday	Sunday	0	0	0	0	1	44.00%	38.00%	18.00%	82.00%	53.66%	46.34%
19 October 2015 / Greenberg Quinlan Rosner Research	14/10/15	19/10/15	2372	3	1	6	Wednesday	Monday	1	0	1	1	1	45.00%	42.00%	12.00%	87.00%	51.72%	48.28%
19 October 2015 / Ipsos MORI	17/10/15	19/10/15	1021	6	1	3	Saturday	Monday	1	0	0	0	0	52.00%	36.00%	12.00%	88.00%	59.09%	40.91%
23 October 2015 / YouGov	22/10/15	23/10/15	1625	4	5	2	Thursday	Friday	0	0	0	1	1	42.00%	39.00%	19.00%	81.00%	51.85%	48.15%
25 October 2015 / ORB	23/10/15	25/10/15	2015	5	7	3	Friday	Sunday	0	0	0	0	1	53.00%	47.00%	0.00%	100.00%	53.00%	47.00%
25 October 2015 / ICM	23/10/15	25/10/15	2049	5	7	3	Friday	Sunday	0	0	0	0	1	45.00%	38.00%	17.00%	83.00%	54.22%	45.78%
29 October 2015 / YouGov	28/10/15	29/10/15	1664	3	4	2	Wednesday	Thursday	0	0	1	1	0	39.00%	41.00%	19.00%	80.00%	48.75%	51.25%
1 November 2015 / ICM	30/10/15	01/11/15	2060	5	7	3	Friday	Sunday	0	0	0	0	1	44.00%	38.00%	18.00%	82.00%	53.66%	46.34%
8 November 2015 / ICM	06/11/15	08/11/15	2024	5	7	3	Friday	Sunday	0	0	0	0	1	46.00%	38.00%	16.00%	84.00%	54.76%	45.24%
15 November 2015 / ICM	13/11/15	15/11/15	2000	5	7	3	Friday	Sunday	0	0	0	0	1	43.00%	38.00%	19.00%	81.00%	53.09%	46.91%
19 November 2015 / ORB	18/11/15	19/11/15	2067	3	4	2	Wednesday	Thursday	0	0	1	1	0	48.00%	52.00%	0.00%	100.00%	48.00%	52.00%
22 November 2015 / ICM	20/11/15	22/11/15	2002	5	7	3	Friday	Sunday	0	0	0	0	1	45.00%	38.00%	17.00%	83.00%	54.22%	45.78%
29 November 2015 / ICM	27/11/15	29/11/15	2053	5	7	3	Friday	Sunday	0	0	0	0	1	43.00%	39.00%	17.00%	82.00%	52.44%	47.56%
6 December 2015 / ICM	04/12/15	06/12/15	2022	5	7	3	Friday	Sunday	0	0	0	0	1	43.00%	39.00%	17.00%	82.00%	52.44%	47.56%
13 December 2015 / ComRes	11/12/15	13/12/15	1001	5	7	3	Friday	Sunday	0	0	0	0	1	56.00%	35.00%	8.00%	91.00%	61.54%	38.46%
13 December 2015 / ICM	11/12/15	13/12/15	2053	5	7	3	Friday	Sunday	0	0	0	0	1	42.00%	41.00%	17.00%	83.00%	50.60%	49.40%
14 December 2015 / Ipsos MORI	12/12/15	14/12/15	529	6	1	3	Saturday	Monday	1	0	0	0	0	58.00%	32.00%	10.00%	90.00%	64.44%	35.56%
17 December 2015 / ORB	16/12/15	17/12/15	2038	3	4	2	Wednesday	Thursday	0	0	1	1	0	52.00%	48.00%	0.00%	100.00%	52.00%	48.00%
18 December 2015 / YouGov	17/12/15	18/12/15	1598	4	5	2	Thursday	Friday	0	0	0	1	1	41.00%	42.00%	17.00%	83.00%	49.40%	50.60%
20 December 2015 / ICM	18/12/15	20/12/15	2017	5	7	3	Friday	Sunday	0	0	0	0	1	45.00%	38.00%	17.00%	83.00%	54.22%	45.78%
10 January 2016 / ICM	08/01/16	10/01/16	2055	5	7	3	Friday	Sunday	0	0	0	0	1	44.00%	38.00%	18.00%	82.00%	53.66%	46.34%
16 January 2016 / Survation	15/01/16	16/01/16	1017	5	6	2	Friday	Saturday	0	0	0	0	1	38.00%	42.00%	21.00%	80.00%	47.50%	52.50%
17 January 2016 / ICM	15/01/16	17/01/16	2023	5	7	3	Friday	Sunday	0	0	0	0	1	42.00%	40.00%	17.00%	82.00%	51.22%	48.78%
21 January 2016 / ORB	20/01/16	21/01/16	2015	3	4	2	Wednesday	Thursday	0	0	1	1	0	52.00%	48.00%	0.00%	100.00%	52.00%	48.00%
24 January 2016 / Opinium	20/01/16	24/01/16	1033	3	7	5	Wednesday	Sunday	0	0	1	1	1	40.00%	41.00%	19.00%	81.00%	49.38%	50.62%
24 January 2016 / ComRes	22/01/16	24/01/16	1006	5	7	3	Friday	Sunday	0	0	0	0	1	54.00%	36.00%	10.00%	90.00%	60.00%	40.00%
24 January 2016 / ICM	22/01/16	24/01/16	2010	5	7	3	Friday	Sunday	0	0	0	0	1	41.00%	41.00%	18.00%	82.00%	50.00%	50.00%
25 January 2016 / Ipsos MORI	23/01/16	25/01/16	513	6	1	3	Saturday	Monday	1	0	0	0	0	55.00%	36.00%	9.00%	91.00%	60.44%	39.56%
25 January 2016 / BMG Research	21/01/16	25/01/16	1511	4	1	5	Thursday	Monday	1	0	0	1	1	44.00%	42.00%	14.00%	86.00%	51.16%	48.84%
28 January 2016 / YouGov	27/01/16	28/01/16	1735	3	4	2	Wednesday	Thursday	0	0	1	1	0	38.00%	42.00%	20.00%	80.00%	47.50%	52.50%
31 January 2016 / ICM	29/01/16	31/01/16	2002	5	7	3	Friday	Sunday	0	0	0	0	1	42.00%	39.00%	19.00%	81.00%	51.85%	48.15%
4 February 2016 / YouGov	03/02/16	04/02/16	1675	3	4	2	Wednesday	Thursday	0	0	1	1	0	36.00%	45.00%	19.00%	81.00%	44.44%	55.56%
7 February 2016 / ICM	05/02/16	07/02/16	2018	5	7	3	Friday	Sunday	0	0	0	0	1	41.00%	42.00%	17.00%	83.00%	49.40%	50.60%
14 February 2016 / ICM	12/02/16	14/02/16	2001	5	7	3	Friday	Sunday	0	0	0	0	1	43.00%	39.00%	18.00%	82.00%	52.44%	47.56%
14 February 2016 / ComRes	11/02/16	14/02/16	1105	4	7	4	Thursday	Sunday	0	0	0	1	1	49.00%	41.00%	10.00%	90.00%	54.44%	45.56%
15 February 2016 / TNS	11/02/16	15/02/16	1120	4	1	5	Thursday	Monday	1	0	0	1	1	36.00%	39.00%	25.00%	75.00%	48.00%	52.00%
20 February 2016 / Survation	20/02/16	20/02/16	1002	6	6	1	Saturday	Saturday	0	0	0	0	0	48.00%	33.00%	19.00%	81.00%	59.26%	40.74%
22 February 2016 / ComRes	19/02/16	22/02/16	1000	5	1	4	Friday	Monday	1	0	0	0	1	52.00%	39.00%	10.00%	91.00%	57.14%	42.86%
22 February 2016 / ICM	19/02/16	22/02/16	2021	5	1	4	Friday	Monday	1	0	0	0	1	42.00%	40.00%	17.00%	82.00%	51.22%	48.78%
25 February 2016 / ORB	24/02/16	25/02/16	2014	3	4	2	Wednesday	Thursday	0	0	1	1	1	48.00%	52.00%	0.00%	100.00%	48.00%	52.00%
25 February 2016 / YouGov	25/02/16	25/02/16	1731	4	4	1	Thursday	Thursday	0	0	0	1	0	41.00%	38.00%	21.00%	79.00%	51.90%	48.10%
29 February 2016 / ICM	26/02/16	29/02/16	2003	5	1	4	Friday	Monday	1	0	0	0	1	41.00%	41.00%	18.00%	82.00%	50.00%	50.00%
6 March 2016 / ICM	04/03/16	06/03/16	2051	5	7	3	Friday	Sunday	0	0	0	0	1	40.00%	41.00%	19.00%	81.00%	49.38%	50.62%
13 March 2016 / ICM	11/03/16	13/03/16	2031	5	7	3	Friday	Sunday	0	0	0	0	1	43.00%	41.00%	16.00%	84.00%	51.19%	48.81%
14 March 2016 / TNS	11/03/16	14/03/16	1216	5	1	4	Friday	Monday	1	0	0	0	1	36.00%	36.00%	28.00%	72.00%	50.00%	50.00%
14 March 2016 / ORB	11/03/16	14/03/16	823	5	1	4	Friday	Monday	1	0	0	0	1	47.00%	49.00%	4.00%	96.00%	48.96%	51.04%
19 March 2016 / Survation	17/03/16	19/03/16	1006	4	6	3	Thursday	Saturday	0	0	0	1	1	46.00%	35.00%	19.00%	81.00%	56.79%	43.21%
20 March 2016 / ComRes	18/03/16	20/03/16	1002	5	7	3	Friday	Sunday	0	0	0	0	1	48.00%	41.00%	11.00%	89.00%	53.93%	46.07%
20 March 2016 / ICM	18/03/16	20/03/16	2000	5	7	3	Friday	Sunday	0	0	0	0	1	41.00%	43.00%	17.00%	84.00%	48.81%	51.19%
Total			95115										Mean	44.96%	40.21%	14.72%	85.18%	52.72%	47.28%

WEDNESDAYS INCLUDED

Fieldwork End Date / Pollster	StartDate	EndDate	Participants	StartDay	EndDay	Length	Startday	EndDay	Monday?	Tuesday?	Wednesday?	Thursday?	Friday?	Remain	Leave	Don't know	All-DK	Remain-DK	Leave-DK
17 September 2015 / YouGov	10/09/15	17/09/15	11171	4	4	8	Thursday	Thursday	1	1	1	1	1	38.00%	40.00%	22.00%	78.00%	48.72%	51.28%
1 October 2015 / YouGov	24/09/15	01/10/15	533	4	4	8	Thursday	Thursday	1	1	1	1	1	38.00%	42.00%	20.00%	80.00%	47.50%	52.50%
7 October 2015 / ICM	07/10/15	07/10/15	1947	3	3	1	Wednesday	Wednesday	1	1	1	1	1	44.00%	39.00%	17.00%	83.00%	53.01%	46.99%
12 October 2015 / YouGov	01/10/15	12/10/15	1030	4	1	12	Thursday	Monday	1	1	1	1	1	43.00%	41.00%	16.00%	84.00%	51.19%	48.81%
19 October 2015 / Greenberg Quinlan Rosner Research	14/10/15	19/10/15	2372	3	1	6	Wednesday	Monday	1	0	1	1	1	45.00%	42.00%	12.00%	87.00%	51.72%	48.28%
29 October 2015 / YouGov	28/10/15	29/10/15	1664	3	4	2	Wednesday	Thursday	0	0	1	1	0	39.00%	41.00%	19.00%	80.00%	48.75%	51.25%
11 November 2015 / Survation	09/11/15	11/11/15	2007	1	3	3	Monday	Wednesday	1	1	1	0	0	39.00%	44.00%	19.00%	83.00%	46.99%	53.01%
17 November 2015 / BMG Research	11/11/15	17/11/15	1528	3	2	7	Wednesday	Tuesday	1	1	1	1	1	43.00%	43.00%	15.00%	86.00%	50.00%	50.00%
19 November 2015 / ORB	18/11/15	19/11/15	2067	3	4	2	Wednesday	Thursday	0	0	1	1	0	48.00%	52.00%	0.00%	100.00%	48.00%	52.00%
3 December 2015 / Survation	30/11/15	03/12/15	10015	1	4	4	Monday	Thursday	1	1	1	1	0	40.00%	42.00%	18.00%	82.00%	48.78%	51.22%
17 December 2015 / ORB	16/12/15	17/12/15	2038	3	4	2	Wednesday	Thursday	0	0	1	1	0	52.00%	48.00%	0.00%	100.00%	52.00%	48.00%
14 January 2016 / Panelbase	08/01/16	14/01/16	2087	5	4	7	Friday	Thursday	1	1	1	1	1	42.00%	45.00%	12.00%	87.00%	48.28%	51.72%
21 January 2016 / ORB	20/01/16	21/01/16	2015	3	4	2	Wednesday	Thursday	0	0	1	1	0	52.00%	48.00%	0.00%	100.00%	52.00%	48.00%
24 January 2016 / Opinium	20/01/16	24/01/16	1033	3	7	5	Wednesday	Sunday	0	0	1	1	1	40.00%	41.00%	19.00%	81.00%	49.38%	50.62%
28 January 2016 / YouGov	27/01/16	28/01/16	1735	3	4	2	Wednesday	Thursday	0	0	1	1	0	38.00%	42.00%	20.00%	80.00%	47.50%	52.50%
4 February 2016 / YouGov	03/02/16	04/02/16	1675	3	4	2	Wednesday	Thursday	0	0	1	1	0	36.00%	45.00%	19.00%	81.00%	44.44%	55.56%
23 February 2016 / BMG Research	17/02/16	23/02/16	1517	3	2	7	Wednesday	Tuesday	1	1	1	1	1	44.00%	41.00%	15.00%	85.00%	51.76%	48.24%
25 February 2016 / ORB	24/02/16	25/02/16	2014	3	4	2	Wednesday	Thursday	0	0	1	1	1	48.00%	52.00%	0.00%	100.00%	48.00%	52.00%
2 March 2016 / YouGov	01/03/16	02/03/16	1705	2	3	2	Tuesday	Wednesday	0	1	1	0	0	40.00%	35.00%	24.00%	75.00%	53.33%	46.67%
3 March 2016 / YouGov	02/03/16	03/03/16	1695	3	4	2	Wednesday	Thursday	0	1	1	1	0	40.00%	37.00%	23.00%	77.00%	51.95%	48.05%
24 March 2016 / ICM	22/03/16	24/03/16	1970	2	4	3	Tuesday	Thursday	0	1	1	1	0	45.00%	43.00%	12.00%	88.00%	51.14%	48.86%
Total			53818										Mean	42.57%	43.00%	14.38%	85.57%	49.74%	50.26%

WEDNESDAYS EXCLUDED

Fieldwork End Date / Pollster	StartDate	EndDate	Participants	StartDay	EndDay	Length	Startday	EndDay	Monday?	Tuesday?	Wednesday?	Thursday?	Friday?	Remain	Leave	Don't know	All-DK	Remain-DK	Leave-DK
4 September 2015 / Survation	03/09/15	04/09/15	1004	4	5	2	Thursday	Friday	0	0	0	1	1	40.00%	43.00%	17.00%	83.00%	48.19%	51.81%
6 September 2015 / ORB	04/09/15	06/09/15	2044	5	7	3	Friday	Sunday	0	0	0	0	1	55.00%	45.00%	0.00%	100.00%	55.00%	45.00%
13 September 2015 / ICM	11/09/15	13/09/15	2006	5	7	3	Friday	Sunday	0	0	0	0	1	43.00%	40.00%	17.00%	83.00%	51.81%	48.19%
20 September 2015 / ICM	18/09/15	20/09/15	2005	5	7	3	Friday	Sunday	0	0	0	0	1	44.00%	37.00%	19.00%	81.00%	54.32%	45.68%
22 September 2015 / Survation	21/09/15	22/09/15	1008	1	2	2	Monday	Tuesday	1	1	0	0	0	43.00%	40.00%	17.00%	83.00%	51.81%	48.19%
27 September 2015 / ICM	25/09/15	27/09/15	2005	5	7	3	Friday	Sunday	0	0	0	0	1	45.00%	38.00%	17.00%	83.00%	54.22%	45.78%
28 September 2015 / ComRes	25/09/15	28/09/15	1009	5	1	4	Friday	Monday	1	0	0	0	1	55.00%	36.00%	8.00%	91.00%	60.44%	39.56%
4 October 2015 / ICM	02/10/15	04/10/15	2034	5	7	3	Friday	Sunday	0	0	0	0	1	42.00%	38.00%	20.00%	80.00%	52.50%	47.50%
11 October 2015 / ICM	09/10/15	11/10/15	2036	5	7	3	Friday	Sunday	0	0	0	0	1	45.00%	36.00%	18.00%	81.00%	55.56%	44.44%
18 October 2015 / ICM	16/10/15	18/10/15	2023	5	7	3	Friday	Sunday	0	0	0	0	1	44.00%	38.00%	18.00%	82.00%	53.66%	46.34%
19 October 2015 / Ipsos MORI	17/10/15	19/10/15	1021	6	1	3	Saturday	Monday	1	0	0	0	0	52.00%	36.00%	12.00%	88.00%	59.09%	40.91%
20 October 2015 / YouGov	19/10/15	20/10/15	1690	1	2	2	Monday	Tuesday	1	1	0	0	0	42.00%	40.00%	17.00%	82.00%	51.22%	48.78%
23 October 2015 / YouGov	22/10/15	23/10/15	1625	4	5	2	Thursday	Friday	0	0	0	1	1	42.00%	39.00%	19.00%	81.00%	51.85%	48.15%
25 October 2015 / ORB	23/10/15	25/10/15	2015	5	7	3	Friday	Sunday	0	0	0	0	1	53.00%	47.00%	0.00%	100.00%	53.00%	47.00%
25 October 2015 / ICM	23/10/15	25/10/15	2049	5	7	3	Friday	Sunday	0	0	0	0	1	45.00%	38.00%	17.00%	83.00%	54.22%	45.78%
27 October 2015 / BMG Research	22/10/15	27/10/15	1467	4	2	6	Thursday	Tuesday	1	1	0	1	1	46.00%	43.00%	12.00%	89.00%	51.69%	48.31%
1 November 2015 / ICM	30/10/15	01/11/15	2060	5	7	3	Friday	Sunday	0	0	0	0	1	44.00%	38.00%	18.00%	82.00%	53.66%	46.34%
8 November 2015 / ICM	06/11/15	08/11/15	2024	5	7	3	Friday	Sunday	0	0	0	0	1	46.00%	38.00%	16.00%	84.00%	54.76%	45.24%
15 November 2015 / ICM	13/11/15	15/11/15	2000	5	7	3	Friday	Sunday	0	0	0	0	1	43.00%	38.00%	19.00%	81.00%	53.09%	46.91%
17 November 2015 / Survation	16/11/15	17/11/15	1546	1	2	2	Monday	Tuesday	1	1	0	0	0	43.00%	40.00%	18.00%	83.00%	51.81%	48.19%
22 November 2015 / ICM	20/11/15	22/11/15	2002	5	7	3	Friday	Sunday	0	0	0	0	1	45.00%	38.00%	17.00%	83.00%	54.22%	45.78%
24 November 2015 / YouGov	20/11/15	24/11/15	4317	5	2	5	Friday	Tuesday	1	1	0	1	1	41.00%	41.00%	18.00%	82.00%	50.00%	50.00%
29 November 2015 / ICM	27/11/15	29/11/15	2053	5	7	3	Friday	Sunday	0	0	0	0	1	43.00%	39.00%	17.00%	82.00%	52.44%	47.56%
6 December 2015 / ICM	04/12/15	06/12/15	2022	5	7	3	Friday	Sunday	0	0	0	0	1	43.00%	39.00%	17.00%	82.00%	52.44%	47.56%
13 December 2015 / ComRes	11/12/15	13/12/15	1001	5	7	3	Friday	Sunday	0	0	0	0	1	56.00%	35.00%	8.00%	91.00%	61.54%	38.46%
13 December 2015 / ICM	11/12/15	13/12/15	2053	5	7	3	Friday	Sunday	0	0	0	0	1	42.00%	41.00%	17.00%	83.00%	50.60%	49.40%
14 December 2015 / Ipsos MORI	12/12/15	14/12/15	529	6	1	3	Saturday	Monday	1	0	0	0	0	58.00%	32.00%	10.00%	90.00%	64.44%	35.56%
18 December 2015 / YouGov	17/12/15	18/12/15	1598	4	5	2	Thursday	Friday	0	0	0	1	1	41.00%	42.00%	17.00%	83.00%	49.40%	50.60%
20 December 2015 / ICM	18/12/15	20/12/15	2017	5	7	3	Friday	Sunday	0	0	0	0	1	45.00%	38.00%	17.00%	83.00%	54.22%	45.78%
10 January 2016 / ICM	08/01/16	10/01/16	2055	5	7	3	Friday	Sunday	0	0	0	0	1	44.00%	38.00%	18.00%	82.00%	53.66%	46.34%
16 January 2016 / Survation	15/01/16	16/01/16	1017	5	6	2	Friday	Saturday	0	0	0	0	1	38.00%	42.00%	21.00%	80.00%	47.50%	52.50%
17 January 2016 / ICM	15/01/16	17/01/16	2023	5	7	3	Friday	Sunday	0	0	0	0	1	42.00%	40.00%	17.00%	82.00%	51.22%	48.78%
24 January 2016 / ComRes	22/01/16	24/01/16	1006	5	7	3	Friday	Sunday	0	0	0	0	1	54.00%	36.00%	10.00%	90.00%	60.00%	40.00%
24 January 2016 / ICM	22/01/16	24/01/16	2010	5	7	3	Friday	Sunday	0	0	0	0	1	41.00%	41.00%	18.00%	82.00%	50.00%	50.00%
25 January 2016 / Ipsos MORI	23/01/16	25/01/16	513	6	1	3	Saturday	Monday	1	0	0	0	0	55.00%	36.00%	9.00%	91.00%	60.44%	39.56%
25 January 2016 / BMG Research	21/01/16	25/01/16	1511	4	1	5	Thursday	Monday	1	0	0	1	1	44.00%	42.00%	14.00%	86.00%	51.16%	48.84%
31 January 2016 / ICM	29/01/16	31/01/16	2002	5	7	3	Friday	Sunday	0	0	0	0	1	42.00%	39.00%	19.00%	81.00%	51.85%	48.15%
7 February 2016 / ICM	05/02/16	07/02/16	2018	5	7	3	Friday	Sunday	0	0	0	0	1	41.00%	42.00%	17.00%	83.00%	49.40%	50.60%
14 February 2016 / ICM	12/02/16	14/02/16	2001	5	7	3	Friday	Sunday	0	0	0	0	1	43.00%	39.00%	18.00%	82.00%	52.44%	47.56%
14 February 2016 / ComRes	11/02/16	14/02/16	1105	4	7	4	Thursday	Sunday	0	0	0	1	1	49.00%	41.00%	10.00%	90.00%	54.44%	45.56%
15 February 2016 / TNS	11/02/16	15/02/16	1120	4	1	5	Thursday	Monday	1	0	0	1	1	36.00%	39.00%	25.00%	75.00%	48.00%	52.00%
16 February 2016 / Ipsos MORI	13/02/16	16/02/16	1001	6	2	4	Saturday	Tuesday	1	1	0	0	0	54.00%	36.00%	10.00%	90.00%	60.00%	40.00%
20 February 2016 / Survation	20/02/16	20/02/16	1002	6	6	1	Saturday	Saturday	0	0	0	0	0	48.00%	33.00%	19.00%	81.00%	59.26%	40.74%
22 February 2016 / ComRes	19/02/16	22/02/16	1000	5	1	4	Friday	Monday	1	0	0	0	1	52.00%	39.00%	10.00%	91.00%	57.14%	42.86%
22 February 2016 / ICM	19/02/16	22/02/16	2021	5	1	4	Friday	Monday	1	0	0	0	1	42.00%	40.00%	17.00%	82.00%	51.22%	48.78%
23 February 2016 / YouGov	22/02/16	23/02/16	3482	1	2	2	Monday	Tuesday	1	1	0	0	0	37.00%	38.00%	25.00%	75.00%	49.33%	50.67%
25 February 2016 / YouGov	25/02/16	25/02/16	1731	4	4	1	Thursday	Thursday	0	0	0	1	0	41.00%	38.00%	21.00%	79.00%	51.90%	48.10%
29 February 2016 / ICM	26/02/16	29/02/16	2003	5	1	4	Friday	Monday	1	0	0	0	1	41.00%	41.00%	18.00%	82.00%	50.00%	50.00%
1 March 2016 / YouGov	29/02/16	01/03/16	2233	1	2	2	Monday	Tuesday	1	1	0	0	0	39.00%	37.00%	24.00%	76.00%	51.32%	48.68%
6 March 2016 / ICM	04/03/16	06/03/16	2051	5	7	3	Friday	Sunday	0	0	0	0	1	40.00%	41.00%	19.00%	81.00%	49.38%	50.62%
13 March 2016 / ICM	11/03/16	13/03/16	2031	5	7	3	Friday	Sunday	0	0	0	0	1	43.00%	41.00%	16.00%	84.00%	51.19%	48.81%
14 March 2016 / TNS	11/03/16	14/03/16	1216	5	1	4	Friday	Monday	1	0	0	0	1	36.00%	36.00%	28.00%	72.00%	50.00%	50.00%
14 March 2016 / ORB	11/03/16	14/03/16	823	5	1	4	Friday	Monday	1	0	0	0	1	47.00%	49.00%	4.00%	96.00%	48.96%	51.04%
19 March 2016 / Survation	17/03/16	19/03/16	1006	4	6	3	Thursday	Saturday	0	0	0	1	1	46.00%	35.00%	19.00%	81.00%	56.79%	43.21%
20 March 2016 / ComRes	18/03/16	20/03/16	1002	5	7	3	Friday	Sunday	0	0	0	0	1	48.00%	41.00%	11.00%	89.00%	53.93%	46.07%
20 March 2016 / ICM	18/03/16	20/03/16	2000	5	7	3	Friday	Sunday	0	0	0	0	1	41.00%	43.00%	17.00%	84.00%	48.81%	51.19%
Total			95246										Mean	44.82%	39.21%	15.91%	84.04%	53.22%	46.78%

THURSDAYS INCLUDED																		
Fieldwork End Date / Pollster	StartDate	EndDate	Participants	StartDay	EndDay	Length	Startday	EndDay	Monday?	Tuesday?	Wednesday?	Thursday?	Friday?	Remain	Leave	Don't know	All-DK	Remain-DK	Leave-DK
4 September 2015 / Survation	03/09/15	04/09/15	1004	4	5	2	Thursday	Friday	0	0	0	1	1	40.00%	43.00%	17.00%	83.00%	48.19%	51.81%
17 September 2015 / YouGov	10/09/15	17/09/15	11171	4	4	8	Thursday	Thursday	1	1	1	1	1	38.00%	40.00%	22.00%	78.00%	48.72%	51.28%
1 October 2015 / YouGov	24/09/15	01/10/15	533	4	4	8	Thursday	Thursday	1	1	1	1	1	38.00%	42.00%	20.00%	80.00%	47.50%	52.50%
7 October 2015 / ICM	07/10/15	07/10/15	1947	3	3	1	Wednesday	Wednesday	1	1	1	1	1	44.00%	39.00%	17.00%	83.00%	53.01%	46.99%
12 October 2015 / YouGov	01/10/15	12/10/15	1030	4	1	12	Thursday	Monday	1	1	1	1	1	43.00%	41.00%	16.00%	84.00%	51.19%	48.81%
19 October 2015 / Greenberg Quinlan Rosner Research	14/10/15	19/10/15	2372	3	1	6	Wednesday	Monday	1	0	1	1	1	45.00%	42.00%	12.00%	87.00%	51.72%	48.28%
23 October 2015 / YouGov	22/10/15	23/10/15	1625	4	5	2	Thursday	Friday	0	0	0	1	1	42.00%	39.00%	19.00%	81.00%	51.85%	48.15%
27 October 2015 / BMG Research	22/10/15	27/10/15	1467	4	2	6	Thursday	Tuesday	1	1	0	1	1	46.00%	43.00%	12.00%	89.00%	51.69%	48.31%
29 October 2015 / YouGov	28/10/15	29/10/15	1664	3	4	2	Wednesday	Thursday	0	0	1	1	0	39.00%	41.00%	19.00%	80.00%	48.75%	51.25%
17 November 2015 / BMG Research	11/11/15	17/11/15	1528	3	2	7	Wednesday	Tuesday	1	1	1	1	1	43.00%	43.00%	15.00%	86.00%	50.00%	50.00%
19 November 2015 / ORB	18/11/15	19/11/15	2067	3	4	2	Wednesday	Thursday	0	0	1	1	0	48.00%	52.00%	0.00%	100.00%	48.00%	52.00%
24 November 2015 / YouGov	20/11/15	24/11/15	4317	5	2	5	Friday	Tuesday	1	1	0	1	1	41.00%	41.00%	18.00%	82.00%	50.00%	50.00%
3 December 2015 / Survation	30/11/15	03/12/15	10015	1	4	4	Monday	Thursday	1	1	1	1	0	40.00%	42.00%	18.00%	82.00%	48.78%	51.22%
17 December 2015 / ORB	16/12/15	17/12/15	2038	3	4	2	Wednesday	Thursday	0	0	1	1	0	52.00%	48.00%	0.00%	100.00%	52.00%	48.00%
18 December 2015 / YouGov	17/12/15	18/12/15	1598	4	5	2	Thursday	Friday	0	0	0	1	1	41.00%	42.00%	17.00%	83.00%	49.40%	50.60%
14 January 2016 / Panelbase	08/01/16	14/01/16	2087	5	4	7	Friday	Thursday	1	1	1	1	1	42.00%	45.00%	12.00%	87.00%	48.28%	51.72%
21 January 2016 / ORB	20/01/16	21/01/16	2015	3	4	2	Wednesday	Thursday	0	0	1	1	0	52.00%	48.00%	0.00%	100.00%	52.00%	48.00%
24 January 2016 / Opinium	20/01/16	24/01/16	1033	3	7	5	Wednesday	Sunday	0	0	1	1	1	40.00%	41.00%	19.00%	81.00%	49.38%	50.62%
25 January 2016 / BMG Research	21/01/16	25/01/16	1511	4	1	5	Thursday	Monday	1	0	0	1	1	44.00%	42.00%	14.00%	86.00%	51.16%	48.84%
28 January 2016 / YouGov	27/01/16	28/01/16	1735	3	4	2	Wednesday	Thursday	0	0	1	1	0	38.00%	42.00%	20.00%	80.00%	47.50%	52.50%
4 February 2016 / YouGov	03/02/16	04/02/16	1675	3	4	2	Wednesday	Thursday	0	0	1	1	0	36.00%	45.00%	19.00%	81.00%	44.44%	55.56%
14 February 2016 / ComRes	11/02/16	14/02/16	1105	4	7	4	Thursday	Sunday	0	0	0	1	1	49.00%	41.00%	10.00%	90.00%	54.44%	45.56%
15 February 2016 / TNS	11/02/16	15/02/16	1120	4	1	5	Thursday	Monday	1	0	0	1	1	36.00%	39.00%	25.00%	75.00%	48.00%	52.00%
23 February 2016 / BMG Research	17/02/16	23/02/16	1517	3	2	7	Wednesday	Tuesday	1	1	1	1	1	44.00%	41.00%	15.00%	85.00%	51.76%	48.24%
25 February 2016 / ORB	24/02/16	25/02/16	2014	3	4	2	Wednesday	Thursday	0	0	1	1	1	48.00%	52.00%	0.00%	100.00%	48.00%	52.00%
25 February 2016 / YouGov	25/02/16	25/02/16	1731	4	4	1	Thursday	Thursday	0	0	0	1	0	41.00%	38.00%	21.00%	79.00%	51.90%	48.10%
3 March 2016 / YouGov	02/03/16	03/03/16	1695	3	4	2	Wednesday	Thursday	0	1	1	1	0	40.00%	37.00%	23.00%	77.00%	51.95%	48.05%
19 March 2016 / Survation	17/03/16	19/03/16	1006	4	6	3	Thursday	Saturday	0	0	0	1	1	46.00%	35.00%	19.00%	81.00%	56.79%	43.21%
24 March 2016 / ICM	22/03/16	24/03/16	1970	2	4	3	Tuesday	Thursday	0	1	1	1	0	45.00%	43.00%	12.00%	88.00%	51.14%	48.86%
Total			66590										Mean	42.79%	42.31%	14.86%	85.10%	50.26%	49.74%

THURSDAYS EXCLUDED																			
Fieldwork End Date / Pollster	StartDate	EndDate	Participants	StartDay	EndDay	Length	Startday	EndDay	Monday?	Tuesday?	Wednesday?	Thursday?	Friday?	Remain	Leave	Don't know	All-DK	Remain-DK	Leave-DK
6 September 2015 / ORB	04/09/15	06/09/15	2044	5	7	3	Friday	Sunday	0	0	0	0	1	55.00%	45.00%	0.00%	100.00%	55.00%	45.00%
13 September 2015 / ICM	11/09/15	13/09/15	2006	5	7	3	Friday	Sunday	0	0	0	0	1	43.00%	40.00%	17.00%	83.00%	51.81%	48.19%
20 September 2015 / ICM	18/09/15	20/09/15	2005	5	7	3	Friday	Sunday	0	0	0	0	1	44.00%	37.00%	19.00%	81.00%	54.32%	45.68%
22 September 2015 / Survation	21/09/15	22/09/15	1008	1	2	2	Monday	Tuesday	1	1	0	0	0	43.00%	40.00%	17.00%	83.00%	51.81%	48.19%
27 September 2015 / ICM	25/09/15	27/09/15	2005	5	7	3	Friday	Sunday	0	0	0	0	1	45.00%	38.00%	17.00%	83.00%	54.22%	45.78%
28 September 2015 / ComRes	25/09/15	28/09/15	1009	5	1	4	Friday	Monday	1	0	0	0	1	55.00%	36.00%	8.00%	91.00%	60.44%	39.56%
4 October 2015 / ICM	02/10/15	04/10/15	2034	5	7	3	Friday	Sunday	0	0	0	0	1	42.00%	38.00%	20.00%	80.00%	52.50%	47.50%
11 October 2015 / ICM	09/10/15	11/10/15	2036	5	7	3	Friday	Sunday	0	0	0	0	1	45.00%	36.00%	18.00%	81.00%	55.56%	44.44%
18 October 2015 / ICM	16/10/15	18/10/15	2023	5	7	3	Friday	Sunday	0	0	0	0	1	44.00%	38.00%	18.00%	82.00%	53.66%	46.34%
19 October 2015 / Ipsos MORI	17/10/15	19/10/15	1021	6	1	3	Saturday	Monday	1	0	0	0	0	52.00%	36.00%	12.00%	88.00%	59.09%	40.91%
20 October 2015 / YouGov	19/10/15	20/10/15	1690	1	2	2	Monday	Tuesday	1	1	0	0	0	42.00%	40.00%	17.00%	82.00%	51.22%	48.78%
25 October 2015 / ORB	23/10/15	25/10/15	2015	5	7	3	Friday	Sunday	0	0	0	0	1	53.00%	47.00%	0.00%	100.00%	53.00%	47.00%
25 October 2015 / ICM	23/10/15	25/10/15	2049	5	7	3	Friday	Sunday	0	0	0	0	1	45.00%	38.00%	17.00%	83.00%	54.22%	45.78%
1 November 2015 / ICM	30/10/15	01/11/15	2060	5	7	3	Friday	Sunday	0	0	0	0	1	44.00%	38.00%	18.00%	82.00%	53.66%	46.34%
8 November 2015 / ICM	06/11/15	08/11/15	2024	5	7	3	Friday	Sunday	0	0	0	0	1	46.00%	38.00%	16.00%	84.00%	54.76%	45.24%
11 November 2015 / Survation	09/11/15	11/11/15	2007	1	3	3	Monday	Wednesday	1	1	1	0	0	39.00%	44.00%	19.00%	83.00%	46.99%	53.01%
15 November 2015 / ICM	13/11/15	15/11/15	2000	5	7	3	Friday	Sunday	0	0	0	0	1	43.00%	38.00%	19.00%	81.00%	53.09%	46.91%
17 November 2015 / Survation	16/11/15	17/11/15	1546	1	2	2	Monday	Tuesday	1	1	0	0	0	43.00%	40.00%	18.00%	83.00%	51.81%	48.19%
22 November 2015 / ICM	20/11/15	22/11/15	2002	5	7	3	Friday	Sunday	0	0	0	0	1	45.00%	38.00%	17.00%	83.00%	54.22%	45.78%
29 November 2015 / ICM	27/11/15	29/11/15	2053	5	7	3	Friday	Sunday	0	0	0	0	1	43.00%	39.00%	17.00%	82.00%	52.44%	47.56%
6 December 2015 / ICM	04/12/15	06/12/15	2022	5	7	3	Friday	Sunday	0	0	0	0	1	43.00%	39.00%	17.00%	82.00%	52.44%	47.56%
13 December 2015 / ComRes	11/12/15	13/12/15	1001	5	7	3	Friday	Sunday	0	0	0	0	1	56.00%	35.00%	8.00%	91.00%	61.54%	38.46%
13 December 2015 / ICM	11/12/15	13/12/15	2053	5	7	3	Friday	Sunday	0	0	0	0	1	42.00%	41.00%	17.00%	83.00%	50.60%	49.40%
14 December 2015 / Ipsos MORI	12/12/15	14/12/15	529	6	1	3	Saturday	Monday	1	0	0	0	0	58.00%	32.00%	10.00%	90.00%	64.44%	35.56%
20 December 2015 / ICM	18/12/15	20/12/15	2017	5	7	3	Friday	Sunday	0	0	0	0	1	45.00%	38.00%	17.00%	83.00%	54.22%	45.78%
10 January 2016 / ICM	08/01/16	10/01/16	2055	5	7	3	Friday	Sunday	0	0	0	0	1	44.00%	38.00%	18.00%	82.00%	53.66%	46.34%
16 January 2016 / Survation	15/01/16	16/01/16	1017	5	6	2	Friday	Saturday	0	0	0	0	1	38.00%	42.00%	21.00%	80.00%	47.50%	52.50%
17 January 2016 / ICM	15/01/16	17/01/16	2023	5	7	3	Friday	Sunday	0	0	0	0	1	42.00%	40.00%	17.00%	82.00%	51.22%	48.78%
24 January 2016 / ComRes	22/01/16	24/01/16	1006	5	7	3	Friday	Sunday	0	0	0	0	1	54.00%	36.00%	10.00%	90.00%	60.00%	40.00%
24 January 2016 / ICM	22/01/16	24/01/16	2010	5	7	3	Friday	Sunday	0	0	0	0	1	41.00%	41.00%	18.00%	82.00%	50.00%	50.00%
25 January 2016 / Ipsos MORI	23/01/16	25/01/16	513	6	1	3	Saturday	Monday	1	0	0	0	0	55.00%	36.00%	9.00%	91.00%	60.44%	39.56%
31 January 2016 / ICM	29/01/16	31/01/16	2002	5	7	3	Friday	Sunday	0	0	0	0	1	42.00%	39.00%	19.00%	81.00%	51.85%	48.15%
7 February 2016 / ICM	05/02/16	07/02/16	2018	5	7	3	Friday	Sunday	0	0	0	0	1	41.00%	42.00%	17.00%	83.00%	49.40%	50.60%
14 February 2016 / ICM	12/02/16	14/02/16	2001	5	7	3	Friday	Sunday	0	0	0	0	1	43.00%	39.00%	18.00%	82.00%	52.44%	47.56%
16 February 2016 / Ipsos MORI	13/02/16	16/02/16	1001	6	2	4	Saturday	Tuesday	1	1	0	0	0	54.00%	36.00%	10.00%	90.00%	60.00%	40.00%
20 February 2016 / Survation	20/02/16	20/02/16	1002	6	6	1	Saturday	Saturday	0	0	0	0	0	48.00%	33.00%	19.00%	81.00%	59.26%	40.74%
22 February 2016 / ComRes	19/02/16	22/02/16	1000	5	1	4	Friday	Monday	1	0	0	0	1	52.00%	39.00%	10.00%	91.00%	57.14%	42.86%
22 February 2016 / ICM	19/02/16	22/02/16	2021	5	1	4	Friday	Monday	1	0	0	0	1	42.00%	40.00%	17.00%	82.00%	51.22%	48.78%
23 February 2016 / YouGov	22/02/16	23/02/16	3482	1	2	2	Monday	Tuesday	1	1	0	0	0	37.00%	38.00%	25.00%	75.00%	49.33%	50.67%
29 February 2016 / ICM	26/02/16	29/02/16	2003	5	1	4	Friday	Monday	1	0	0	0	1	41.00%	41.00%	18.00%	82.00%	50.00%	50.00%
1 March 2016 / YouGov	29/02/16	01/03/16	2233	1	2	2	Monday	Tuesday	1	1	0	0	0	39.00%	37.00%	24.00%	76.00%	51.32%	48.68%
2 March 2016 / YouGov	01/03/16	02/03/16	1705	2	3	2	Tuesday	Wednesday	0	1	1	0	0	40.00%	35.00%	24.00%	75.00%	53.33%	46.67%
6 March 2016 / ICM	04/03/16	06/03/16	2051	5	7	3	Friday	Sunday	0	0	0	0	1	40.00%	41.00%	19.00%	81.00%	49.38%	50.62%
13 March 2016 / ICM	11/03/16	13/03/16	2031	5	7	3	Friday	Sunday	0	0	0	0	1	43.00%	41.00%	16.00%	84.00%	51.19%	48.81%
14 March 2016 / TNS	11/03/16	14/03/16	1216	5	1	4	Friday	Monday	1	0	0	0	1	36.00%	36.00%	28.00%	72.00%	50.00%	50.00%
14 March 2016 / ORB	11/03/16	14/03/16	823	5	1	4	Friday	Monday	1	0	0	0	1	47.00%	49.00%	4.00%	96.00%	48.96%	51.04%
20 March 2016 / ComRes	18/03/16	20/03/16	1002	5	7	3	Friday	Sunday	0	0	0	0	1	48.00%	41.00%	11.00%	89.00%	53.93%	46.07%
20 March 2016 / ICM	18/03/16	20/03/16	2000	5	7	3	Friday	Sunday	0	0	0	0	1	41.00%	43.00%	17.00%	84.00%	48.81%	51.19%
Total			82474										Mean	45.06%	39.00%	15.88%	84.06%	53.49%	46.51%

FRIDAYS INCLUDED

Fieldwork End Date / Pollster	StartDate	EndDate	Participants	StartDay	EndDay	Length	Startday	EndDay	Monday?	Tuesday?	Wednesday?	Thursday?	Friday?	Remain	Leave	Don't know	All-DK	Remain-DK	Leave-DK
4 September 2015 / Survation	03/09/15	04/09/15	1004	4	5	2	Thursday	Friday	0	0	0	1	1	40.00%	43.00%	17.00%	83.00%	48.19%	51.81%
6 September 2015 / ORB	04/09/15	06/09/15	2044	5	7	3	Friday	Sunday	0	0	0	0	1	55.00%	45.00%	0.00%	100.00%	55.00%	45.00%
13 September 2015 / ICM	11/09/15	13/09/15	2006	5	7	3	Friday	Sunday	0	0	0	0	1	43.00%	40.00%	17.00%	83.00%	51.81%	48.19%
17 September 2015 / YouGov	10/09/15	17/09/15	11171	4	4	8	Thursday	Thursday	1	1	1	1	1	38.00%	40.00%	22.00%	78.00%	48.72%	51.28%
20 September 2015 / ICM	18/09/15	20/09/15	2005	5	7	3	Friday	Sunday	0	0	0	0	1	44.00%	37.00%	19.00%	81.00%	54.32%	45.68%
27 September 2015 / ICM	25/09/15	27/09/15	2005	5	7	3	Friday	Sunday	0	0	0	0	1	45.00%	38.00%	17.00%	83.00%	54.22%	45.78%
28 September 2015 / ComRes	25/09/15	28/09/15	1009	5	1	4	Friday	Monday	1	0	0	0	1	55.00%	36.00%	8.00%	91.00%	60.44%	39.56%
1 October 2015 / YouGov	24/09/15	01/10/15	533	4	4	8	Thursday	Thursday	1	1	1	1	1	38.00%	42.00%	20.00%	80.00%	47.50%	52.50%
4 October 2015 / ICM	02/10/15	04/10/15	2034	5	7	3	Friday	Sunday	0	0	0	0	1	42.00%	38.00%	20.00%	80.00%	52.50%	47.50%
7 October 2015 / ICM	07/10/15	07/10/15	1947	3	3	1	Wednesday	Wednesday	1	1	1	1	1	44.00%	39.00%	17.00%	83.00%	53.01%	46.99%
11 October 2015 / ICM	09/10/15	11/10/15	2036	5	7	3	Friday	Sunday	0	0	0	0	1	45.00%	36.00%	18.00%	81.00%	55.56%	44.44%
12 October 2015 / YouGov	01/10/15	12/10/15	1030	4	1	12	Thursday	Monday	1	1	1	1	1	43.00%	41.00%	16.00%	84.00%	51.19%	48.81%
18 October 2015 / ICM	16/10/15	18/10/15	2023	5	7	3	Friday	Sunday	0	0	0	0	1	44.00%	38.00%	18.00%	82.00%	53.66%	46.34%
19 October 2015 / Greenberg Quinlan Rosner Research	14/10/15	19/10/15	2372	3	1	6	Wednesday	Monday	1	0	1	1	1	45.00%	42.00%	12.00%	87.00%	51.72%	48.28%
23 October 2015 / YouGov	22/10/15	23/10/15	1625	4	5	2	Thursday	Friday	0	0	0	1	1	42.00%	39.00%	19.00%	81.00%	51.85%	48.15%
25 October 2015 / ORB	23/10/15	25/10/15	2015	5	7	3	Friday	Sunday	0	0	0	0	1	53.00%	47.00%	0.00%	100.00%	53.00%	47.00%
25 October 2015 / ICM	23/10/15	25/10/15	2049	5	7	3	Friday	Sunday	0	0	0	0	1	45.00%	38.00%	17.00%	83.00%	54.22%	45.78%
27 October 2015 / BMG Research	22/10/15	27/10/15	1467	4	2	6	Thursday	Tuesday	1	1	0	1	1	46.00%	43.00%	12.00%	89.00%	51.69%	48.31%
1 November 2015 / ICM	30/10/15	01/11/15	2060	5	7	3	Friday	Sunday	0	0	0	0	1	44.00%	38.00%	18.00%	82.00%	53.66%	46.34%
8 November 2015 / ICM	06/11/15	08/11/15	2024	5	7	3	Friday	Sunday	0	0	0	0	1	46.00%	38.00%	16.00%	84.00%	54.76%	45.24%
15 November 2015 / ICM	13/11/15	15/11/15	2000	5	7	3	Friday	Sunday	0	0	0	0	1	43.00%	38.00%	19.00%	81.00%	53.09%	46.91%
17 November 2015 / BMG Research	11/11/15	17/11/15	1528	3	2	7	Wednesday	Tuesday	1	1	1	1	1	43.00%	43.00%	15.00%	86.00%	50.00%	50.00%
22 November 2015 / ICM	20/11/15	22/11/15	2002	5	7	3	Friday	Sunday	0	0	0	0	1	45.00%	38.00%	17.00%	83.00%	54.22%	45.78%
24 November 2015 / YouGov	20/11/15	24/11/15	4317	5	2	5	Friday	Tuesday	1	1	0	1	1	41.00%	41.00%	18.00%	82.00%	50.00%	50.00%
29 November 2015 / ICM	27/11/15	29/11/15	2053	5	7	3	Friday	Sunday	0	0	0	0	1	43.00%	39.00%	17.00%	82.00%	52.44%	47.56%
6 December 2015 / ICM	04/12/15	06/12/15	2022	5	7	3	Friday	Sunday	0	0	0	0	1	43.00%	39.00%	17.00%	82.00%	52.44%	47.56%
13 December 2015 / ComRes	11/12/15	13/12/15	1001	5	7	3	Friday	Sunday	0	0	0	0	1	56.00%	35.00%	8.00%	91.00%	61.54%	38.46%
13 December 2015 / ICM	11/12/15	13/12/15	2053	5	7	3	Friday	Sunday	0	0	0	0	1	42.00%	41.00%	17.00%	83.00%	50.60%	49.40%
18 December 2015 / YouGov	17/12/15	18/12/15	1598	4	5	2	Thursday	Friday	0	0	0	1	1	41.00%	42.00%	17.00%	83.00%	49.40%	50.60%
20 December 2015 / ICM	18/12/15	20/12/15	2017	5	7	3	Friday	Sunday	0	0	0	0	1	45.00%	38.00%	17.00%	83.00%	54.22%	45.78%
10 January 2016 / ICM	08/01/16	10/01/16	2055	5	7	3	Friday	Sunday	0	0	0	0	1	44.00%	38.00%	18.00%	82.00%	53.66%	46.34%
14 January 2016 / Panelbase	08/01/16	14/01/16	2087	5	4	7	Friday	Thursday	1	1	1	1	1	42.00%	45.00%	12.00%	87.00%	48.28%	51.72%
16 January 2016 / Survation	15/01/16	16/01/16	1017	5	6	2	Friday	Saturday	0	0	0	0	1	38.00%	42.00%	21.00%	80.00%	47.50%	52.50%
17 January 2016 / ICM	15/01/16	17/01/16	2023	5	7	3	Friday	Sunday	0	0	0	0	1	42.00%	40.00%	17.00%	82.00%	51.22%	48.78%
24 January 2016 / Opinium	20/01/16	24/01/16	1033	3	7	5	Wednesday	Sunday	0	0	1	1	1	40.00%	41.00%	19.00%	81.00%	49.38%	50.62%
24 January 2016 / ComRes	22/01/16	24/01/16	1006	5	7	3	Friday	Sunday	0	0	0	0	1	54.00%	36.00%	10.00%	90.00%	60.00%	40.00%
24 January 2016 / ICM	22/01/16	24/01/16	2010	5	7	3	Friday	Sunday	0	0	0	0	1	41.00%	41.00%	18.00%	82.00%	50.00%	50.00%
25 January 2016 / BMG Research	21/01/16	25/01/16	1511	4	1	5	Thursday	Monday	1	0	0	1	1	44.00%	42.00%	14.00%	86.00%	51.16%	48.84%
31 January 2016 / ICM	29/01/16	31/01/16	2002	5	7	3	Friday	Sunday	0	0	0	0	1	42.00%	39.00%	19.00%	81.00%	51.85%	48.15%
7 February 2016 / ICM	05/02/16	07/02/16	2018	5	7	3	Friday	Sunday	0	0	0	0	1	41.00%	42.00%	17.00%	83.00%	49.40%	50.60%
14 February 2016 / ICM	12/02/16	14/02/16	2001	5	7	3	Friday	Sunday	0	0	0	0	1	43.00%	39.00%	18.00%	82.00%	52.44%	47.56%
14 February 2016 / ComRes	11/02/16	14/02/16	1105	4	7	4	Thursday	Sunday	0	0	0	1	1	49.00%	41.00%	10.00%	90.00%	54.44%	45.56%
15 February 2016 / TNS	11/02/16	15/02/16	1120	4	1	5	Thursday	Monday	1	0	0	1	1	36.00%	39.00%	25.00%	75.00%	48.00%	52.00%
22 February 2016 / ComRes	19/02/16	22/02/16	1000	5	1	4	Friday	Monday	1	0	0	0	1	52.00%	39.00%	10.00%	91.00%	57.14%	42.86%
22 February 2016 / ICM	19/02/16	22/02/16	2021	5	1	4	Friday	Monday	1	0	0	0	1	42.00%	40.00%	17.00%	82.00%	51.22%	48.78%
23 February 2016 / BMG Research	17/02/16	23/02/16	1517	3	2	7	Wednesday	Tuesday	1	1	1	1	1	44.00%	41.00%	15.00%	85.00%	51.76%	48.24%
25 February 2016 / ORB	24/02/16	25/02/16	2014	3	4	2	Wednesday	Thursday	0	0	1	1	1	48.00%	52.00%	0.00%	100.00%	48.00%	52.00%
29 February 2016 / ICM	26/02/16	29/02/16	2003	5	1	4	Friday	Monday	1	0	0	0	1	41.00%	41.00%	18.00%	82.00%	50.00%	50.00%
6 March 2016 / ICM	04/03/16	06/03/16	2051	5	7	3	Friday	Sunday	0	0	0	0	1	40.00%	41.00%	19.00%	81.00%	49.38%	50.62%
13 March 2016 / ICM	11/03/16	13/03/16	2031	5	7	3	Friday	Sunday	0	0	0	0	1	43.00%	41.00%	16.00%	84.00%	51.19%	48.81%
14 March 2016 / TNS	11/03/16	14/03/16	1216	5	1	4	Friday	Monday	1	0	0	0	1	36.00%	36.00%	28.00%	72.00%	50.00%	50.00%
14 March 2016 / ORB	11/03/16	14/03/16	823	5	1	4	Friday	Monday	1	0	0	0	1	47.00%	49.00%	4.00%	96.00%	48.96%	51.04%
19 March 2016 / Survation	17/03/16	19/03/16	1006	4	6	3	Thursday	Saturday	0	0	0	1	1	46.00%	35.00%	19.00%	81.00%	56.79%	43.21%
20 March 2016 / ComRes	18/03/16	20/03/16	1002	5	7	3	Friday	Sunday	0	0	0	0	1	48.00%	41.00%	11.00%	89.00%	53.93%	46.07%
20 March 2016 / ICM	18/03/16	20/03/16	2000	5	7	3	Friday	Sunday	0	0	0	0	1	41.00%	43.00%	17.00%	84.00%	48.81%	51.19%
Total			104722										Mean	44.05%	40.29%	15.58%	84.35%	52.17%	47.83%

FRIDAYS EXCLUDED

Fieldwork End Date / Pollster	StartDate	EndDate	Participants	StartDay	EndDay	Length	Startday	EndDay	Monday?	Tuesday?	Wednesday?	Thursday?	Friday?	Remain	Leave	Don't know	All-DK	Remain-DK	Leave-DK
22 September 2015 / Survation	21/09/15	22/09/15	1008	1	2	2	Monday	Tuesday	1	1	0	0	0	43.00%	40.00%	17.00%	83.00%	51.81%	48.19%
19 October 2015 / Ipsos MORI	17/10/15	19/10/15	1021	6	1	3	Saturday	Monday	1	0	0	0	0	52.00%	36.00%	12.00%	88.00%	59.09%	40.91%
20 October 2015 / YouGov	19/10/15	20/10/15	1690	1	2	2	Monday	Tuesday	1	1	0	0	0	42.00%	40.00%	17.00%	82.00%	51.22%	48.78%
29 October 2015 / YouGov	28/10/15	29/10/15	1664	3	4	2	Wednesday	Thursday	0	0	1	1	0	39.00%	41.00%	19.00%	80.00%	48.75%	51.25%
11 November 2015 / Survation	09/11/15	11/11/15	2007	1	3	3	Monday	Wednesday	1	1	1	0	0	39.00%	44.00%	19.00%	83.00%	46.99%	53.01%
17 November 2015 / Survation	16/11/15	17/11/15	1546	1	2	2	Monday	Tuesday	1	1	0	0	0	43.00%	40.00%	18.00%	83.00%	51.81%	48.19%
19 November 2015 / ORB	18/11/15	19/11/15	2067	3	4	2	Wednesday	Thursday	0	0	1	1	0	48.00%	52.00%	0.00%	100.00%	48.00%	52.00%
3 December 2015 / Survation	30/11/15	03/12/15	10015	1	4	4	Monday	Thursday	1	1	1	1	0	40.00%	42.00%	18.00%	82.00%	48.78%	51.22%
14 December 2015 / Ipsos MORI	12/12/15	14/12/15	529	6	1	3	Saturday	Monday	1	0	0	0	0	58.00%	32.00%	10.00%	90.00%	64.44%	35.56%
17 December 2015 / ORB	16/12/15	17/12/15	2038	3	4	2	Wednesday	Thursday	0	0	1	1	0	52.00%	48.00%	0.00%	100.00%	52.00%	48.00%
21 January 2016 / ORB	20/01/16	21/01/16	2015	3	4	2	Wednesday	Thursday	0	0	1	1	0	52.00%	48.00%	0.00%	100.00%	52.00%	48.00%
25 January 2016 / Ipsos MORI	23/01/16	25/01/16	513	6	1	3	Saturday	Monday	1	0	0	0	0	55.00%	36.00%	9.00%	91.00%	60.44%	39.56%
28 January 2016 / YouGov	27/01/16	28/01/16	1735	3	4	2	Wednesday	Thursday	0	0	1	1	0	38.00%	42.00%	20.00%	80.00%	47.50%	52.50%
4 February 2016 / YouGov	03/02/16	04/02/16	1675	3	4	2	Wednesday	Thursday	0	0	1	1	0	36.00%	45.00%	19.00%	81.00%	44.44%	55.56%
16 February 2016 / Ipsos MORI	13/02/16	16/02/16	1001	6	2	4	Saturday	Tuesday	1	1	0	0	0	54.00%	36.00%	10.00%	90.00%	60.00%	40.00%
20 February 2016 / Survation	20/02/16	20/02/16	1002	6	6	1	Saturday	Saturday	0	0	0	0	0	48.00%	33.00%	19.00%	81.00%	59.26%	40.74%
23 February 2016 / YouGov	22/02/16	23/02/16	3482	1	2	2	Monday	Tuesday	1	1	0	0	0	37.00%	38.00%	25.00%	75.00%	49.33%	50.67%
25 February 2016 / YouGov	25/02/16	25/02/16	1731	4	4	1	Thursday	Thursday	0	0	0	1	0	41.00%	38.00%	21.00%	79.00%	51.90%	48.10%
1 March 2016 / YouGov	29/02/16	01/03/16	2233	1	2	2	Monday	Tuesday	1	1	0	0	0	39.00%	37.00%	24.00%	76.00%	51.32%	48.68%
2 March 2016 / YouGov	01/03/16	02/03/16	1705	2	3	2	Tuesday	Wednesday	0	1	1	0	0	40.00%	35.00%	24.00%	75.00%	53.33%	46.67%
3 March 2016 / YouGov	02/03/16	03/03/16	1695	3	4	2	Wednesday	Thursday	0	1	1	1	0	40.00%	37.00%	23.00%	77.00%	51.95%	48.05%
24 March 2016 / ICM	22/03/16	24/03/16	1970	2	4	3	Tuesday	Thursday	0	1	1	1	0	45.00%	43.00%	12.00%	88.00%	51.14%	48.86%
Total			44342										Mean	44.59%	40.14%	15.27%	84.73%	52.52%	47.48%
